# Epigenome-wide association study of incident type 2 diabetes: a meta-analysis of five prospective European cohorts

**DOI:** 10.1007/s00125-022-05652-2

**Published:** 2022-02-15

**Authors:** Eliza Fraszczyk, Annemieke M. W. Spijkerman, Yan Zhang, Stefan Brandmaier, Felix R. Day, Li Zhou, Paul Wackers, Martijn E. T. Dollé, Vincent W. Bloks, Xīn Gào, Christian Gieger, Jaspal Kooner, Jennifer Kriebel, H. Susan J. Picavet, Wolfgang Rathmann, Ben Schöttker, Marie Loh, W. M. Monique Verschuren, Jana V. van Vliet-Ostaptchouk, Nicholas J. Wareham, John C. Chambers, Ken K. Ong, Harald Grallert, Hermann Brenner, Mirjam Luijten, Harold Snieder

**Affiliations:** 1 Department of Epidemiology, University of Groningen, University Medical Center Groningen, Groningen, the Netherlands; 2 Centre for Health Protection, National Institute for Public Health and the Environment (RIVM), Bilthoven, the Netherlands; 3 Centre for Nutrition, Prevention and Health services, National Institute for Public Health and the Environment (RIVM), Bilthoven, the Netherlands; 4 Division of Clinical Epidemiology and Aging Research, German Cancer Research Center, Heidelberg, Germany; 5 Research Unit of Molecular Epidemiology, Institute of Epidemiology, Helmholtz Zentrum München, Neuherberg, Germany; 6 German Center for Diabetes Research (DZD), Neuherberg, Germany; 7 MRC Epidemiology Unit, Institute of Metabolic Science, University of Cambridge School of Clinical Medicine, Cambridge, UK; 8 Lee Kong Chian School of Medicine, Nanyang Technological University, Singapore, Singapore; 9 Department of Pediatrics, Section of Molecular Metabolism and Nutrition, University of Groningen, University Medical Center Groningen, Groningen, the Netherlands; 10 Department of Cardiology, Ealing Hospital, Ealing, UK; 11 Imperial College Healthcare NHS Trust, London, UK; 12 MRC-PHE Centre for Environment and Health, Imperial College London, London, UK; 13 National Heart and Lung Institute, Imperial College London, London, UK; 14 Institute for Biometrics and Epidemiology, German Diabetes Center, Auf’m Hennekamp, Duesseldorf, Germany; 15 Network Aging Research, University of Heidelberg, Heidelberg, Germany; 16 Julius Center for Health Sciences and Primary Care, University Medical Center Utrecht, Utrecht University, Utrecht, the Netherlands; 17 Genomics Coordination Center, Department of Genetics, University of Groningen, University Medical Center Groningen, Groningen, the Netherlands; 18 Department of Genetics, University of Groningen, University Medical Center Groningen, Groningen, the Netherlands; 19 Department of Epidemiology and Biostatistics, Imperial College London, London, UK; 20 Department of Paediatrics, University of Cambridge School of Clinical Medicine, Cambridge, UK

**Keywords:** Biomarkers, DNA methylation, Epigenetics, Epigenome-wide association studies, Meta-analysis, Prediction, Prospective studies, Type 2 diabetes

## Abstract

**Aims/hypothesis:**

Type 2 diabetes is a complex metabolic disease with increasing prevalence worldwide. Improving the prediction of incident type 2 diabetes using epigenetic markers could help tailor prevention efforts to those at the highest risk. The aim of this study was to identify predictive methylation markers for incident type 2 diabetes by combining epigenome-wide association study (EWAS) results from five prospective European cohorts.

**Methods:**

We conducted a meta-analysis of EWASs in blood collected 7–10 years prior to type 2 diabetes diagnosis. DNA methylation was measured with Illumina Infinium Methylation arrays. A total of 1250 cases and 1950 controls from five longitudinal cohorts were included: Doetinchem, ESTHER, KORA1, KORA2 and EPIC-Norfolk. Associations between DNA methylation and incident type 2 diabetes were examined using robust linear regression with adjustment for potential confounders. Inverse-variance fixed-effects meta-analysis of cohort-level individual CpG EWAS estimates was performed using METAL. The methylGSA R package was used for gene set enrichment analysis. Confirmation of genome-wide significant CpG sites was performed in a cohort of Indian Asians (LOLIPOP, UK).

**Results:**

The meta-analysis identified 76 CpG sites that were differentially methylated in individuals with incident type 2 diabetes compared with control individuals (*p* values <1.1 × 10^−7^). Sixty-four out of 76 (84.2%) CpG sites were confirmed by directionally consistent effects and *p* values <0.05 in an independent cohort of Indian Asians. However, on adjustment for baseline BMI only four CpG sites remained genome-wide significant, and addition of the 76 CpG methylation risk score to a prediction model including established predictors of type 2 diabetes (age, sex, BMI and HbA_1c_) showed no improvement (AUC 0.757 vs 0.753). Gene set enrichment analysis of the full epigenome-wide results clearly showed enrichment of processes linked to insulin signalling, lipid homeostasis and inflammation.

**Conclusions/interpretation:**

By combining results from five European cohorts, and thus significantly increasing study sample size, we identified 76 CpG sites associated with incident type 2 diabetes. Replication of 64 CpGs in an independent cohort of Indian Asians suggests that the association between DNA methylation levels and incident type 2 diabetes is robust and independent of ethnicity. Our data also indicate that BMI partly explains the association between DNA methylation and incident type 2 diabetes. Further studies are required to elucidate the underlying biological mechanisms and to determine potential causal roles of the differentially methylated CpG sites in type 2 diabetes development.

**Graphical abstract:**

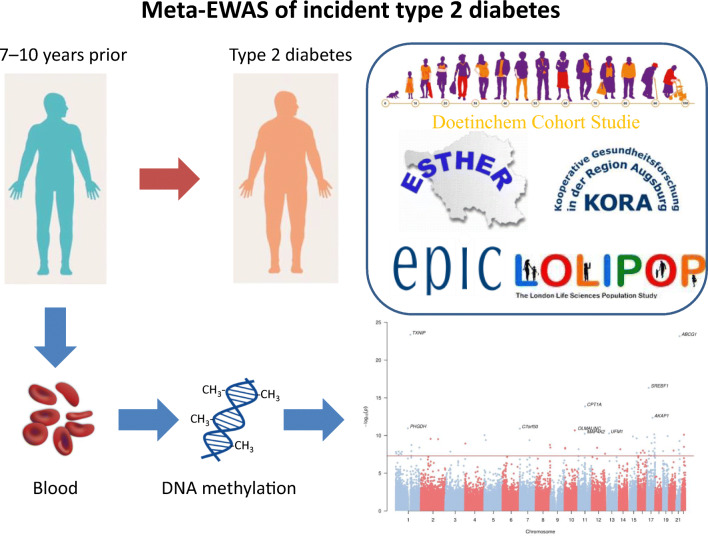

**Supplementary Information:**

The online version contains peer-reviewed but unedited supplementary material available at 10.1007/s00125-022-05652-2.

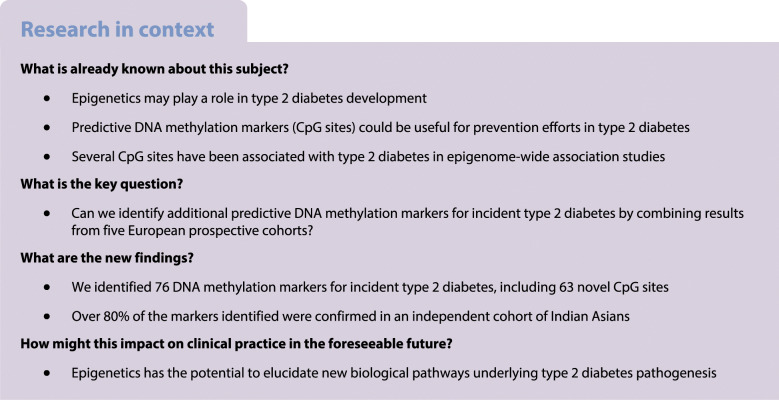



## Introduction

Type 2 diabetes is a complex metabolic disease characterised by chronically elevated blood glucose levels, insulin resistance and beta cell failure and their interaction with obesity and physical inactivity [1–3]. Recent genome-wide association studies identified over 400 genetic variants associated with type 2 diabetes; however, these variants explain only a minor part of the type 2 diabetes heritability [4]. To identify missing components of type 2 diabetes aetiology, researchers started to examine gene–environment interactions and epigenetic mechanisms [5–7]. Improving the prediction of incident type 2 diabetes using epigenetic markers could help tailor prevention efforts focused on those at the highest risk. Moreover, epigenetics could also elucidate new pathophysiological pathways involved in type 2 diabetes development.

Recent epigenome-wide association studies (EWASs) in blood have identified differentially methylated CpG sites (DMS), in individuals with vs without type 2 diabetes, in genes such as *TXNIP*, *ABCG1* and *SREBF1* [8–10]. Further replication in a case–control sample of an independent cohort study confirmed the robustness of those associations with type 2 diabetes [11]. However, most of the EWASs reported so far used a cross-sectional approach, whereas it is well-known that type 2 diabetes develops over a timespan of many years before it is clinically manifest [1]. At present, only two studies examining methylation changes prior to type 2 diabetes onset have been reported: the first in the LOLIPOP cohort including 2664 participants [8]; and the second in EPIC-Norfolk including 1264 participants [12]. In both studies, increased methylation in the *ABCG1* and *SREBF1* genes and decreased methylation in the *TXNIP* gene at baseline were associated with incident type 2 diabetes.

The aim of this study was to identify additional DNA methylation markers for incident type 2 diabetes. For this, we combined results from five European prospective cohorts to increase statistical power with a focus on European ancestry in the discovery stage. The cohorts involved are the Doetinchem Cohort Study [13] from the Netherlands, the ESTHER (Epidemiologische Studie zu Chancen der Verhütung, Früherkennung und optimierten Therapie chronischer Erkrankungen in der älteren Bevölkerung) [14] and KORA (Cooperative Health Research in the Region Augsburg) [15] cohort studies from Germany and the EPIC (European Prospective Investigation into Cancer) Norfolk [16] study from the UK. We conducted a meta-analysis using DNA methylation data from EWASs obtained from blood samples collected 7–10 years prior to type 2 diabetes diagnosis. A total of 1250 cases and 1950 controls were included in this meta-analysis. Furthermore, the significant DMS obtained from the meta-analysis were tested for replication in a longitudinal cohort of Indian Asians (The London Life Sciences Prospective Population Study [LOLIPOP]) to evaluate the robustness of the associations observed [8].

## Methods

### Participating cohorts

In the EWAS meta-analysis we included five European cohorts (one from the Netherlands, three from Germany and one from the UK). The cohorts involved were the Doetinchem Cohort Study [13], ESTHER [14], KORA [15] and EPIC-Norfolk [16]. Two independent subcohorts from the KORA cohort were selected for EWAS analyses, designated as KORA1 (including KORA F4 and FF4 studies) and KORA2 (including KORA S3 and S4 studies). In total, five independent EWASs for incident type 2 diabetes were performed. Replication was performed in a cohort study of Indian Asians (LOLIPOP) from London, UK [8]. A general description of the cohort and characteristics of the individuals included in the current study are presented in Tables 1 and 2 (see electronic supplementary material [ESM] Methods for further details). All participants provided informed consent and the studies were approved by ethics committees.

**Table 1:** Characteristics of the cohorts included in the meta-analysis for incident type 2 diabetes

Characteristic	Doetinchem Cohort Study	ESTHER	KORA1	KORA2	EPIC-Norfolk	LOLIPOP cohort - replication
Cohort design	Population-based cohort study	Population-based cohort study	Population-based cohort study	Population-based cohort study	Population-based cohort study	Population-based cohort study
Ancestry	European	European	European	European	European	Indian Asian
Country	Netherlands	Germany	Germany	Germany	UK	England
Exclusion of prevalent T2D cases at baseline	Based on self-reported prevalent T2D diagnosis/ T2D drug use/random glucose ≥11.1 mmol/l	Based on self- or GP- prevalent T2D diagnosis/T2D drug use and HbA_1c_ ≥48 mmol/mol (6.5%)	Based on OGTT ≥11.1 mmol/l	Based on OGTT ≥11.1 mmol/l and/or self- or GP-reported prevalent T2D diagnosis/T2D drug use	Based on self-report of T2D, doctor-diagnosed T2D, T2D drug use or evidence of T2D after baseline with a date of diagnosis earlier than the baseline recruitment date	Based on T2D drug use/ fasting glucose concentration >7 mmol/l and HbA_1c_ >48 mmol/mol (6.5%)
Definition used for incident T2D during follow-up	Self-reported, confirmed by the GP	Self- or GP-reported usage of glucose-lowering drugs during 14 years of follow-up	GP diagnosis based on OGTT test (plasma glucose ≤11.1 mmol/l)	Self-reported, confirmed by the GP	Self-reported, confirmed by the GP or other sources (general practice diabetes register, local hospital diabetes register, hospital admissions data and Office of National Statistics mortality data with coding for diabetes)	GP diagnosis based on fasting glucose >7 mmol/l, or HbA_1c_ >48 mmol/mol (6.5%)
Control definition	Healthy, no family history of T2D, no gestational diabetes and normogylcaemic throughout cohort follow-up time (random glucose <7.8 mmol/l)	Lack of self- or GP-reported prescriptions of glucose-lowering drugs and HbA_1c_ levels <48 mmol/mol (6.5%) at baseline and follow-up	No T2D at baseline and follow-up based on OGTT test (plasma glucose ≤11.1 mmol/l)	Lack of self- or GP-reported T2D, no glucose-lowering medication	Random sub-cohort of non-cases	Not receiving treatment for T2D and with a fasting glucose <7 mmol/l and HbA_1c_ <48 mmol/mol (6.5%)
Case–control matching	Age (±2 years), sex and measurement round	Age (±2 years), sex and measurement round	Age (±2 years), sex and measurement round	Age (±2 years), sex, measurement round and observation time until diagnosis (years)	Not-matched	Age (groups of 5 years) and sex
DNA methylation array	Illumina Infinium Methylation EPIC (450 K subset used)	Illumina Infinium HumanMethylation450K	Illumina Infinium HumanMethylation450K	Illumina Infinium HumanMethylation450K	Illumina Infinium HumanMethylation450K	Illumina Infinium HumanMethylation450K
Total no. of CpG sites included in meta-analysis	424,750	416,716	450,549	470,870	442,920	466,186

GP, general practitioner; T2D, type 2 diabetes

**Table 2:** Baseline characteristics of incident type 2 diabetes cases and controls per cohort in the meta-analysis

Characteristic	Doetinchem Cohort Study		ESTHER		KORA1		KORA2		EPIC-Norfolk		LOLIPOP - replication	
	Incident T2D	Control	Incident T2D	Control	Incident T2D	Control	Incident T2D	Control	Incident T2D	Non-cases	Incident T2D	Control
*n*	132	133	255	724	103	206	197	186	563	701	1072	1587
Age, years	50.4 ± 9.2	50.3 ± 9.2	62 ± 6.5	62 ± 6.3	62.7 ± 8.6	62.5 ± 8.3	57.7 ± 9.0	57.3 ± 8.9	61.6 ± 8.1	59.1 ± 9.2	52.6 ± 10.2	49.9±9.8
Men, *n* (%)	71 (54)	72 (54)	120 (47.1)	344 (47.5)	62 (60)	124 (60)	107 (54)	100 (54)	326 (58)	294 (42)	721 (67.3)	1081 (68.1)
Follow-up time, years^a^	10.5 ± 2.1	9.8 ± 1.8	7.2 ± 3.5	8.9 ± 5.0	7	7	7	7	6.25 ± 2.4	NA	NA	NA
Fasting glucose, mmol/l	NA	NA	5.5 ± 0.9	5.0 ± 0.8	5.9 ± 0.6	5.2 ± 0.4	NA	NA	6.7 ± 3.6	4.4 ± 1.0	5.5 ± 0.6	5.1 ± 0.5
Random glucose, mmol/l	6.0 ± 1.0	5.0 ± 0.7	NA	NA	NA	NA	NA	NA	NA	NA	NA	NA
HbA_1c_, mmol/mol^b^	NA	NA	38.5	35.8	NA	NA	NA	NA	47.4	36.2	39.9	34.4
HbA_1c_, %	NA	NA	5.67 ± 0.37	5.43 ± 0.38	NA	NA	NA	NA	6.49 ± 1.30	5.46 ± 0.33	5.80 ± 0.50	5.30 ± 0.90
BMI, kg/m^2^	28.4 ± 4	25.5 ± 3.7	29.3 ± 4.5	26.6 ± 4.1	30.4 ± 4.4	28.1 ± 4.4	31.0 ± 4.6	27.5 ± 4.1	29.2 ± 4.5	25.6 ± 3.6	28.9 ± 4.6	26.7 ± 3.9
Current smoking, *n* (%)	48 (36.4)	39 (29.3)	42 (16.4)	153(21.1)	10 (10)	21 (10)	49 (25)	28 (15)	82 (14.6)	104 (14.8)	101 (9.4)	134 (8.4)
HDL-cholesterol, mmol/l	1.14 ± 0.3	1.32 ± 0.4	1.26 ± 0.3	1.39 ± 0.4	NA	NA	1.21 ± 0.34	1.43 ± 0.43	1.21 ± 0.37	1.50 ± 0.46	1.2 ± 0.3	1.3 ± 0.3
SBP, mmHg	132 ± 18	124 ± 17	142 ± 19	139 ± 20	NA	NA	140 ± 20	134 ± 20	144 ± 18	135 ± 19	134.6 ± 19.1	129.6 ± 18.6
DBP, mmHg	84 ± 11	78 ± 9	85 ± 9	83± 10	NA	NA	84 ± 12	82 ± 11	87 ± 13	83 ± 11	82.9 ± 11.1	81.1± 10.4

Data are shown as mean ± SD or *n* (%)

^a^Follow-up time exactly 7 years in KORA1 and KORA2

^b^HbA_1c_ calculated based on equation: (10.93 × HbA_1c_ in %) − 23.5

DBP, diastolic BP; NA, data not available; SBP, systolic BP; T2D: type 2 diabetes

### Type 2 diabetes diagnosis

The EWAS in Doetinchem, ESTHER, KORA1, KORA2 and LOLIPOP were performed as nested case–control studies of incident type 2 diabetes, with controls matched on age, sex and measurement round. In EPIC-Norfolk, EWAS was performed as a nested case-cohort study with random selection of non-cases. In all cohorts, participants with prevalent type 2 diabetes at baseline were excluded (Table 1). Definitions of incident type 2 diabetes cases and controls varied between cohorts (Table 1). Further details are listed in Table 1 and ESM Methods (Phenotype and covariates).

### Methylation measurements and quality control

DNA extracted from whole blood was bisulphite converted and hybridised to Illumina Infinium Methylation arrays (either the 450K array [KORA, ESTHER, EPIC-Norfolk, LOLIPOP] or the EPIC array [Doetinchem]). Quality control and normalisation of methylation data was conducted by each cohort separately using their own pipeline; details for each cohort are given in ESM Methods.

### Cohort-specific statistical analysis

For each cohort, we independently ran EWAS models according to the same standardised analysis plan (ESM Methods), using robust linear regression models. Normalised β values for methylation intensity at each individual CpG site were modelled as the dependent variable and incident type 2 diabetes as a binary predictor variable. Additional covariates included age, sex, estimated cell types using the Houseman algorithm [17] and batches (model 1). Additionally, we adjusted the model for baseline BMI (model 2). In sensitivity analyses, both model 1 (model 1.1) and model 2 (model 2.1) were additionally adjusted for smoking (three categories: current; never; ever smoker) and follow-up time (years between sample collection for DNA methylation measurements and diagnosis of type 2 diabetes [equivalent year for matched controls]). For additional models we calculated percentile reduction/attenuation of effect sizes compared with model 1.

### Meta-analysis and replication

Inverse-variance fixed-effects meta-analyses of cohort-level individual CpG EWAS estimates were performed using METAL [18]. We corrected for multiple testing by applying a stringent genome-wide significant *p* value <1.1 × 10^−7^ (i.e. 0.05/450k). Potential heterogeneity between studies was quantified using the *I*^2^ measure (the percentage of variance explained by study heterogeneity) and CpG sites with *I*^2^ > 60% and heterogeneity *p* value <0.05 were highlighted. We also highlighted all significant DMS listed as polymorphic or cross-hybridising CpG sites [19]. For polymorphic CpG sites, we used Hartigan’s dip test to evaluate the possible binomial distribution of DNA methylation levels in methylation data of the Doetinchem cohort [20]. We used the HumanMethylation450 v1.2 Manifest File (https://support.illumina.com/downloads/infinium_humanmethylation450_product_files.html) and the R package ‘FDb.InfiniumMethylation.hg19’ version 2.2.0 (https://bioconductor.org/packages/FDb.InfiniumMethylation.hg19/) to annotate to the nearest gene for each CpG. Furthermore, we checked for overlap between our significant DMS and previously published EWAS results related to blood-based incident and prevalent type 2 diabetes, blood lipids, BMI and BP [8, 11, 12, 21–30]. All genome-wide significant CpG sites associated with incident type 2 diabetes were used for replication in an independent cohort of Indian Asians (LOLIPOP). CpGs were considered replicated if they had directionally consistent effects and a *p* value <0.05 (nominal significance). Furthermore, we checked the correlation of effect sizes between discovery and replication stages. To test the predictive ability of the 76 markers for incident type 2 diabetes as an outcome, a methylation risk score (MRS) was calculated based on the summation of the 76 CpGs weighted by the effect sizes from an alternative model of the EPIC-Norfolk dataset [12], which used incident type 2 diabetes as the dependent variable (β values represented the OR per 1% methylation change). Then, receiver operating characteristic curve analyses were performed to provide estimates for AUC in the independent LOLIPOP cohort. We tested models predicting incident type 2 diabetes by the MRS only (model M1), by established phenotypic risk factors only, including age, sex, BMI and HbA_1c_ (model M2) and combining both (model M3). We additionally adjusted models M1, M2 and M3 for cell type distributions (models M4, M5, M6, respectively). To investigate the predictive capacity of CpG sites not reaching genome-wide significance (i.e. *p*>1 × 10^−7^), we compared AUC values from MRSs based on four increasingly lenient *p* value thresholds (*p*<1 × 10^−7^, *p*<1 × 10^−6^, *p*<1 × 10^−5^ and *p*<1 × 10^−4^) with increasing numbers of CpG sites. We performed those analyses in the European-ancestry Doetinchem cohort based on results from leave-one-cohort-out EWAS meta-analysis (see ESM Methods for details).

### Gene set enrichment analysis, transcription factor analysis and association with gene expression

Using the full genome-wide results of model 1 from the meta-analysis, we performed gene set enrichment analysis with the methylGSA R package to relate CpG sites to their biological function [31]. We included Kyoto Encyclopedia of Genes and Genomes (KEGG) and Reactome pathways as well as Gene Ontology (GO) terms available in the package. We corrected for multiple testing using false discovery rate (FDR) <5% [32].

Next, we focused on the 76 genome-wide significant DMS and performed a transcription factor (TF) enrichment analysis using the web-based ChIP-X Enrichment Analysis 3 (ChEA3) tool [33]. The enriched TFs were ranked based on Fisher’s exact test (*p* value <0.01).

To additionally look-up previously reported associations of phenotypes/diseases with genetic variants located in or near associated CpG sites, we submitted a list of gene names nearest to the 76 DMS from our EWAS meta-analysis to the NHGRI-EBI GWAS Catalog (https://www.ebi.ac.uk/gwas/, accessed 25 May 2020). Similarly, we queried the list of 76 DMS in the EWAS catalog (http://www.ewascatalog.org/, accessed 15 February 2021). We highlighted associations related to metabolic traits, lipid traits, BP and obesity.

Furthermore, we investigated the association between our 76 genome-wide significant DMS, gene expression levels in blood and SNPs using publicly available expression quantitative trait methylation (eQTM) results from the BIOS consortium (https://www.genenetwork.nl/biosqtlbrowser/, accessed 9 July 2020) and methylation quantitative trait loci (meQTL) from GoDMC (http://mqtldb.godmc.org.uk/, accessed 20 July 2021).

## Results

### Characteristics of the meta-analysis cohorts

Baseline characteristics of the cohorts participating in the discovery meta-analysis and replication are presented in Table 2. The mean age at baseline ranged from 50.3 to 62.7 years across cohorts, and the proportion of men ranged from 42% to 68.1% for both incident type 2 diabetes cases and controls. The mean follow-up time between DNA methylation measurements in blood and type 2 diabetes diagnosis ranged from 6.25 to 10.5 years across cohorts. Already at baseline, we observed a higher mean BMI in incident type 2 diabetes cases compared with controls in all cohorts. Similarly, baseline indicators of hyperglycaemia (i.e. fasting glucose and/or HbA_1c_) were higher in incident type 2 diabetes cases compared with controls in ESTHER, KORA1, EPIC-Norfolk and LOLIPOP. We observed differences in smoking status between incident type 2 diabetes cases across cohorts, with the proportion of current smokers ranging from 9.4% in LOLIPOP to 36.4% in the Doetinchem cohort (Table 2).

### Meta-analysis results of discovery

**Fig. 1:**
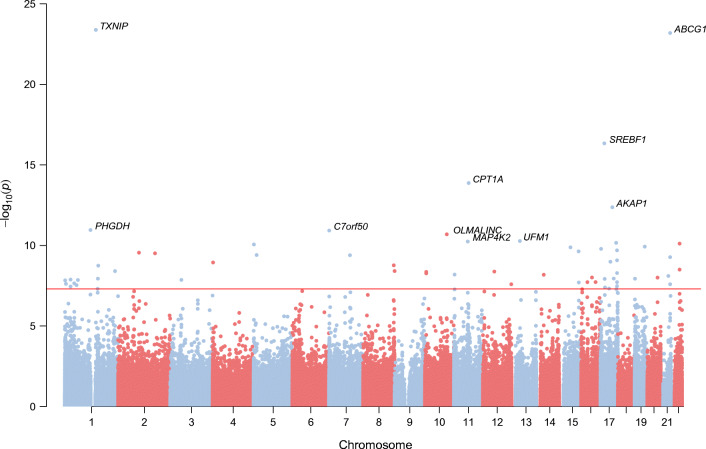
Manhattan plot showing 76 genome-wide significant CpG sites (above red line, *p*<1.1×10^−7^) associated with incident type 2 diabetes in five European cohorts (*N*=1250 cases/1950 controls). Gene annotations for the ten most significant CpG sites are indicated in the plot; *y*-axis shows negative log of associated *p* value

Combining the results of the five discovery EWAS, we identified 76 genome-wide significant DMS using model 1 (λ = 1.189; QQ plots per cohort and for the whole meta-analysis for all models are presented in ESM Fig. 1). Of these, 63 DMS have not been previously reported to be associated with incident type 2 diabetes. The 76 DMS were annotated to 65 genes. Some of these genes had multiple CpG sites annotated to them: *LGALS3BP* (5); *ABCG1* (3); *SYNGR1* (3); *SLC9A1* (2); *PFKFB3* (2); and *NAP1L4* (2) (Table 3). The results are summarised in a Manhattan plot (Fig. 1), showing the distribution of CpG sites across the genome. Based on principal component analysis (PCA) performed in the Doetinchem dataset, 32 out of the 76 CpG sites were considered independent signals (90% of variance explained). CpG site cg11800635 was listed as a probe with potential cross-hybridisation and 11 CpG sites were listed as polymorphic CpGs (Table 3). However, for eight out of those 11 CpG sites available in the Doetinchem dataset, we found no evidence of binomial methylation distributions, suggesting lack of confounding by the underlying SNP (dip-test *p* values 0.5–0.99). Of the 76 DMS identified, 20 DMS (26%) showed *I*^2^ > 60% suggesting considerable heterogeneity between studies (*p*<0.05; Table 3); for each of these 20 CpG sites, we made forest plots (ESM Fig. 2). Despite high, statistically significant heterogeneity estimates, only one site showed a difference in the direction of the association between cohorts (cg19169154 in KORA1; *I*^2^ = 66.2%). Also, KORA1 showed large differences in effect size for cg19693031 (*I*^2^ = 89.2%) and cg11269166 (*I*^2^ = 79.7%). For some sites, two clusters of cohorts with similar effect sizes seemed to be present (e.g. cg24678869 [*I*^2^ = 71.4%]). Otherwise, despite the high heterogeneity estimates, effect estimates were broadly consistent between cohorts.

**Table 3:** The 76 genome-wide significant DMS for incident type 2 diabetes from meta-analysis based on five European discovery cohorts

Illumina ID	Nearest gene	CHR	Position	Gene position	Relation to CpG island	Effect size	SE	*p* value	FDR	Direction across studies^a^	Heterogeneity *I*^2^	Heterogeneity *p* value	GWAS catalog reported metabolic traits	Correlation with gene expression in blood (FDR)
cg19693031	*TXNIP*	1	145441552	3′UTR		−0.0198	0.0020	4.4 × 10^−24^	1.6 × 10^−18^	-----	89.2	1.8 × 10^−7^		9.3 × 10^−6^
cg06500161	*ABCG1*	21	43656587	Body	S_Shore	0.0111	0.0011	6.8 × 10^−24^	1.6 × 10^−18^	+++++	68	0.01		<1 × 10^−7^
cg11024682^b^	*SREBF1*	17	17730094	Body	S_Shelf	0.0094	0.0011	4.8 × 10^−17^	7.6 × 10^−12^	+++++	46	0.12		<1 × 10^−7^
cg00574958	*CPT1A*	11	68607622	5′UTR	N_Shore	−0.0053	0.0007	1.4 × 10^−14^	1.7 × 10^−9^	-----	76.3	0.002	Lipid metabolism phenotypes Lipid traits (pleiotropy) (HIPO component 1)	<1 × 10^−7^
cg05778424	*AKAP1*	17	55169508	5′UTR		0.0080	0.0011	4.4 × 10^−13^	4.2 × 10^−8^	+++++	60.9	0.04		
cg14476101	*PHGDH*	1	120255992	Body	S_Shore	−0.0151	0.0022	1.1 × 10^−11^	8.1 × 10^−7^	-----	49.6	0.09	Metabolic traits Total cholesterol levels	<1 × 10^−7^
cg04816311	*C7orf50*	7	1066650	Body	N_Shore	0.0118	0.0017	1.2 × 10^−11^	8.1 × 10^−7^	+++++	70.8	0.01	Total cholesterol levels LDL-cholesterol	<1 × 10^−7^
cg07504977^c^	*OLMALINC*	10	102131012		N_Shelf	0.0114	0.0017	2.1 × 10^−11^	1.2 × 10^−6^	+++++	0	0.60		
cg19750657^c^	*UFM1*	13	38935967	3′UTR		0.0096	0.0015	5.4 × 10^−11^	2.7 × 10^−6^	+++++	0	0.54		
cg06378491^c^	*MAP4K2*	11	64564012	Body		0.0047	0.0007	5.8 × 10^−11^	2.7 × 10^−6^	+++++	46.8	0.11		
cg14020176^b^	*SLC9A3R1*	17	72764985	3′UTR		0.0087	0.0013	7.0 × 10^−11^	3.0 × 10^−6^	+?+++	34.9	0.20		
cg06397161	*SYNGR1*	22	39760059	Body		0.0095	0.0015	7.8 × 10^−11^	3.1 × 10^−6^	?++++	54.1	0.09		
cg06940720^c^	*LPCAT1*	5	1526929		S_Shelf	0.0072	0.0011	8.8 × 10^−11^	3.2 × 10^−6^	+++++	0	0.64		
cg02711608^b^	*SLC1A5*	19	47287964	1st Exon	N_Shelf	−0.0094	0.0015	1.2 × 10^−10^	4.1 × 10^−6^	??---	13.8	0.31		0.02
cg06192883^c^	*MYO5C*	15	52554171	Body		0.0085	0.0013	1.3 × 10^−10^	4.2 × 10^−6^	+++++	77	0.002		
cg09664445^c^	*CLUH*	17	2612406	5′UTR	N_Shore	0.0059	0.0009	1.6 × 10^−10^	4.9 × 10^−6^	+++++	0	0.41		0.02
cg14870271^b,c^	*LGALS3BP*	17	76976010	1st Exon		0.0084	0.0013	2.0 × 10^−10^	5.7 × 10^−6^	+++++	51.7	0.08		<1 × 10^−7^
cg18568872^c^	*ZNF710*	15	90606494	5′UTR	N_Shelf	0.0060	0.0009	2.4 × 10^−10^	6.2 × 10^–6^	+++++	0	0.60		
cg12257439^c^	*FER1L5*	2	97360893	Body		0.0055	0.0009	2.8 × 10^−10^	7.1 × 10^−6^	+++++	22.2	0.27		
cg11269166^c^	*METTL8*	2	172203847	Body		0.0068	0.0011	3.1 × 10^−10^	7.4 × 10^−6^	+++++	79.7	0.001		
cg14956201^c^	*TRIO*	5	14358153	Body		0.0082	0.0013	4.0 × 10^–10^	8.8 × 10^−6^	+++++	0	0.56		
cg17540192^c^	*TECPR1*	7	97875259	Body		0.0051	0.0008	4.1 × 10^−10^	8.8 × 10^−6^	+++++	72.9	0.01		
cg27243685^c^	*ABCG1*	21	43642366	Body	S_Shelf	0.0060	0.0010	5.4 × 10^−10^	1.1 × 10^−5^	+++++	0	0.89		<1 × 10^−7^
cg11202345^c^	*LGALS3BP*	17	76976057	1stExon		0.0078	0.0013	8.4 × 10^−10^	1.7 × 10^−5^	+++++	0	0.41		<1 × 10^−7^
cg15020801^c^	*PNPO*	17	46022809	Body		0.0073	0.0012	1.0 × 10^−9^	2.0 × 10^−5^	+++++	43.6	0.13		
cg21480264^c^	*POLN*	4	2137264	Body		0.0059	0.0010	1.2 × 10^−9^	2.1 × 10^−5^	+++++	0	0.82	Diastolic BP	
cg08788930^c^	*DENND3*	8	142201685	Body		0.0074	0.0012	1.7 × 10^−9^	3.0 × 10^−5^	+++++	26.5	0.24		0.002
cg25217710^c^	*BCAN*	1	156609523		N_Shelf	0.0054	0.0009	1.8 × 10^−9^	3.1 × 10^−5^	+++++	56.9	0.05		
cg22650271^c^	*SYNGR1*	22	39760165	Body		0.0056	0.0010	3.2 × 10^−9^	5.2 × 10^−5^	+++++	69.9	0.01	Cholesteryl ester levels	3.0 × 10^−4^
cg10639435^b,c^	*ZNF250*	8	146104221	3′UTR		0.0080	0.0014	3.9 × 10^−9^	6.0 × 10^−5^	+++++	0	0.92		
cg01101459^c^	*LINC01132*	1	234871477			0.0070	0.0012	4.0 × 10^−9^	6.0 × 10^−5^	+++++	75.4	0.003	LDL-cholesterol, LDL-cholesterol levels, Total cholesterol levels	
cg03691549^c^	*LOC283335*	12	53443911	5′UTR	S_Shelf	0.0059	0.0010	4.2 × 10^−9^	6.3 × 10^−5^	+++++	0	0.58		
cg26262157^c^	*PFKFB3*	10	6214079	Body		−0.0084	0.0014	4.4 × 10^−9^	6.4 × 10^−5^	-----	61.2	0.04	Latent autoimmune diabetes	
cg04927537^c^	*LGALS3BP*	17	76976091	TSS200		0.0107	0.0018	5.2 × 10^−9^	7.3 × 10^−5^	+++++	0	0.51		<1 × 10^−7^
cg08994060	*PFKFB3*	10	6214026	Body		−0.0101	0.0017	5.4 × 10^−9^	7.4 × 10^−5^	-----	63.7	0.03	Latent autoimmune diabetes	
cg13059136^c^	*NAP1L4*	11	2986541	TSS1500		0.0080	0.0014	6.5 × 10^−9^	8.5 × 10^−5^	+++++	64.8	0.02	T2D HDL-cholesterol levels	
cg21234053^b,c^	*CFL2*	14	35163420			0.0150	0.0026	6.7 × 10^−9^	8.5 × 10^−5^	??+++	51.7	0.13		
cg08309687	*LINC00649*	21	35320596			−0.0112	0.0019	7.9 × 10^−9^	9.9 × 10^−5^	-----	42.8	0.14		4.7 × 10^−4^
cg02879453^b,c^	*ADCY7*	16	50321818	TSS200		0.0080	0.0014	9.8 × 10^−9^	1.2 × 10^−4^	+++++	48.3	0.10		
cg24259291^c^	*ZNFX1*	20	47874072	Body		0.0046	0.0008	1.0 × 10^−8^	1.2 × 10^−4^	+++++	0	0.49		
cg26846781^b,c^	*KCNH6*	17	61620942	Body		0.0045	0.0008	1.1 × 10^−8^	1.3 × 10^−4^	+++++	49.8	0.09		
cg16097041^c^	*FLAD1*	1	154965544	3′UTR		0.0061	0.0011	1.2 × 10^−8^	1.3 × 10^−4^	+++++	0	0.51		
cg01373896^c^	*KLF16*	19	1854724	Body	Island	0.0062	0.0011	1.2 × 10^−8^	1.3 × 10^−4^	+++++	48.9	0.10	BMI	
cg19169154^c^	*MFAP4*	17	19287978	Body		0.0049	0.0009	1.2 × 10^−8^	1.3 × 10^−4^	++-++	66.2	0.02		
cg13300580^c^	*SLC9A1*	1	27440539	Body		0.0047	0.0008	1.3 × 10^−8^	1.4 × 10^−4^	+++++	70.2	0.01		
cg23021329^c^	*TLR9*	3	52256186	Body	S_Shore	0.0051	0.0009	1.4 × 10^−8^	1.4 × 10^−4^	+++++	34.4	0.19		
cg25001190^c^	*NFIA*	1	61668835	Body		−0.0100	0.0018	1.4 × 10^−8^	1.4 × 10^−4^	-----	0	0.80	HDL-cholesterol levels	
cg02050917^c^	*SKI*	1	2173571	Body		0.0069	0.0012	1.5 × 10^−8^	1.5 × 10^−4^	+++++	55.1	0.06	Systolic BP	
cg07719604^c^	*E2F4*	16	67232460	TSS1500	N_Shore	0.0074	0.0013	1.9 × 10^−8^	1.8 × 10^−4^	+++++	0	0.45	HDL-cholesterol	
cg26663590^c^	*NFATC2IP*	16	28959310		S_Shore	0.0083	0.0015	1.9 × 10^−8^	1.8 × 10^−4^	+++++	63.2	0.03	BMI	
cg17836612^c^	*LGALS3BP*	17	76976357	TSS1500		0.0063	0.0011	1.9 × 10^−8^	1.8 × 10^−4^	+++++	10.3	0.35		<1 × 10^−7^
cg20507228^b,c^	*MAN2A2*	15	91460071	Body		0.0126	0.0022	2.0 × 10^−8^	1.8 × 10^−4^	+?+++	0	0.78		
cg04682775^c^	*SLC6A9*	1	44495089	5′UTR	N_Shore	0.0065	0.0012	2.3 × 10^−8^	2.0 × 10^−4^	+++++	0	0.44		
cg24145109^b,c^	*MIR4689*	1	5806951			0.0152	0.0027	2.4 × 10^−8^	2.1 × 10^−4^	??+++	0	0.42		
cg10192877^c^	*ABCG1*	21	43641690	Body	S_Shore	0.0038	0.0007	2.6 × 10^−8^	2.2 × 10^−4^	+++++	55.1	0.06		<1 × 10^−7^
cg21703988^c^	*EP400*	12	132549404	Body		0.0050	0.0009	2.6 × 10^−8^	2.2 × 10^−4^	+++++	6.6	0.37		
cg17901584^c^	*DHCR24*	1	55353706	TSS1500	S_Shore	−0.0093	0.0017	2.9 × 10^−8^	2.4 × 10^−4^	-----	15.7	0.31		
cg25178683^c^	*LGALS3BP*	17	76976267	TSS1500		0.0084	0.0015	3.4 × 10^−8^	2.8 × 10^−4^	+++++	0	0.42		<1 × 10^−7^
cg25130381	*SLC9A1*	1	27440721	Body		0.0056	0.0010	3.6 × 10^−8^	2.9 × 10^−4^	+++++	50.5	0.09		
cg25649826^c^	*USP22*	17	20938740	Body		0.0058	0.0011	4.1 × 10^−8^	3.2 × 10^−4^	+++++	16.8	0.31		
cg20212624^c^	*CNP*	17	40123227	Body	S_Shelf	0.0067	0.0012	4.8 × 10^−8^	3.7 × 10^−4^	+++++	0	0.93		
cg07567724^c^	*GATAD2B*	1	153777721	3′UTR		0.0076	0.0014	5.0 × 10^−8^	3.8 × 10^−4^	?++++	0	0.75		
cg16861241^c^	*RNF157-AS1*	17	74138396	TSS1500	S_Shore	0.0052	0.0010	5.2 × 10^−8^	3.9 × 10^−4^	+++++	0	0.82		
cg03819286^c^	*MGRN1*	16	4673974	TSS1500	N_Shore	0.0060	0.0011	5.3 × 10^−8^	3.9 × 10^−4^	+++++	67.1	0.02		
cg02079413^c^	*NAP1L4*	11	2986505	TSS1500		0.0073	0.0014	5.4 × 10^−8^	3.9 × 10^−4^	+++++	0	0.46	T2D HDL-cholesterol levels	
cg23722778^c^	*ENPP4*	6	46112967	3′UTR		−0.0086	0.0016	6.9 × 10^−8^	5.0 × 10^−4^	--?-?	0	0.60		
cg11800635^b,c^	*DOK1*	2	74783088	Body	S_Shore	0.0088	0.0016	7.1 × 10^−8^	5.0 × 10^−4^	+?+++	0	0.98		<1 × 10^−7^ <1 × 10^−7^ 6.16 × 10^−5^
cg25316512^c^	*ENO2*	12	7032991	TSS1500	N_Shelf	0.0045	0.0008	7.3 × 10^−8^	5.1 × 10^−4^	+++++	0	0.42		
cg09294084^c^	*MCF2L*	13	113646732	Body	N_Shore	0.0107	0.0020	7.6 × 10^−8^	5.3 × 10^−4^	+++++	0	0.68	Systolic BP CVD	
cg20784591^c^	*PILRA*	7	99972461	Body		0.0041	0.0008	8.2 × 10^−8^	5.5 × 10^−4^	+++++	73.3	0.005		
cg03497652^c^	*ANKS3*	16	4751569	Body		0.0085	0.0016	8.3 × 10^−8^	5.6 × 10^−4^	+++++	51.7	0.08	HDL-cholesterol levels	0.02
cg24678869^c^	*DENND4B*	1	153919638	TSS1500	S_Shore	0.0042	0.0008	8.6 × 10^−8^	5.6 × 10^−4^	+++++	71.4	0.01		
cg12322877^c^	*ASPSCR1*	17	79963213	Body	S_Shore	0.0115	0.0022	8.7 × 10^−8^	5.6 × 10^−4^	+++++	66.6	0.02	Waist/hip ratio	
cg09072148^b,c^	*NRXN2*	11	64491639	TSS1500	S_Shore	0.0036	0.0007	8.7 × 10^−8^	5.6 × 10^−4^	+++++	24.9	0.26	BMI	
cg14524754^b,c^	*B3GNTL1*	17	80925103	Body	N_Shelf	0.0069	0.0013	9.1 × 10^−8^	5.8 × 10^−4^	+++++	0	0.81		
cg17194270^c^	*SYNGR1*	22	39759992	Body		0.0092	0.0017	1.0 × 10^−7^	0.0006	+++++	0	0.58	Cholesteryl ester levels	0.01

^a^Order of the studies: Doetinchem, ESTHER, KORA1, KORA2, EPIC-Norfolk

^b^Polymorphic or non-specific probe

^c^Novel findings

CHR, chromosome; T2D, type 2 diabetes

As a sensitivity analysis, we evaluated the impact of smoking and follow-up time from sample collection until type 2 diabetes diagnosis. With this additional adjustment (model 1.1) there was a reduction in the number of significant DMS from 76 to 47 (ESM Table 1; follow-up time not available for EPIC-Norfolk non-cases and LOLIPOP). Adjustment for baseline BMI (model 2) and for BMI, smoking and follow-up time (model 2.1) revealed that the number of significant DMS associated with incident type 2 diabetes decreased from 76 to 4 and 3, respectively (still including the two top CpG sites at the *TXNIP* and *ABCG1* genes; ESM Tables 2 and 3). The attenuation of effect sizes across all models per CpG site is presented in ESM Table 4. Mean attenuation for all 76 CpG sites was 3% in model 1.1, while in models 2 and 2.1 the mean attenuation of effects was 22% and 26%, respectively. The correlation of effect sizes between models for all 76 DMS was very high and varied between 0.98 and 0.99 (ESM Fig. 3).

### Comparison with previous EWASs of incident and prevalent type 2 diabetes, lipids, BMI and BP

Previously, 13 of the 76 DMS had been reported to be associated with incident type 2 diabetes [8, 12] and nine with prevalent type 2 diabetes [11, 24], all with consistent directions of effect (ESM Table 5). Furthermore, 33 of the 76 DMS (43%) overlapped with BMI EWAS results [21, 27–30], with consistent direction of the effects, and 12 DMS (16%) overlapped with blood lipid EWAS results, including triacylglycerols, total cholesterol, LDL-cholesterol and HDL-cholesterol [25, 26]. Additionally, five DMS (7%) had previously been reported in EWASs on BP [22, 23] (ESM Table 5).

### Replication

Out of the 76 genome-wide significant DMS, 64 (84.2%) showed significant, directionally consistent association with incident type 2 diabetes in Indian Asians in model 1 (*p*<0.05; ESM Table 6). Using models 1.1, 2 and 2.1, 40 out of 47 (85%), three out of four (75%) and two out of three (67%) DMS, respectively, were replicated in the LOLIPOP cohort (ESM Tables 1–3). Although we observed a substantial attenuation of effect sizes of 47% in our replication (ESM Table 4), the correlation of effect sizes between discovery and replication stages was high (*r* = 0.91; ESM Fig. 3). Next, we combined the effects from the discovery and replication cohorts for the 76 DMS in a meta-analysis. In model 1, 63 DMS showed genome-wide significant associations with incident type 2 diabetes (*p*<1.1 × 10^−7^), whereas in models 1.1, 2 and 2.1 the number of genome-wide significant DMS increased, respectively, from 47, 4 and 3 in discovery only to 59, 18 and 10 in discovery and replication combined (ESM Table 6). Despite the high replication rate of 84.2%, we did observe considerable heterogeneity between discovery and replication, greater than that seen between discovery cohorts alone (in model 1, 53% of DMS showed significant [*p*<0.05] heterogeneity in combined analysis compared with 26% in discovery cohorts only).

The MRS based on 76 CpG sites showed limited predictive ability for incident type 2 diabetes (model M1, AUC = 0.591) in the LOLIPOP cohort (ESM Fig. 4). Moreover, the addition of the MRS to a prediction model including established predictors of type 2 diabetes (age, sex, BMI and HbA_1c_) showed no improvement (model M2, AUC = 0.753 vs model M3, AUC = 0.757). Additional adjustment for cell type distributions in these models did not change these conclusions (models M4, M5, M6). In the Doetinchem cohort we observed a slight improvement in AUC after adding an MRS based on genome-wide significant CpG sites (model M1 [age, sex, BMI, cell types, batch], AUC = 0.735; model M2 [age, sex, BMI, cell types, batch and MRS], AUC = 0.755; ESM Fig. 5). However, adding additional CpG sites based on less-stringent *p* value thresholds did not improve the AUC, indicating the limited predictive capacity of CpG sites that did not achieve genome-wide significance in the current meta-analysis (ESM Fig. 6).

### Gene set enrichment analysis and associations with gene expression and SNPs

The results of gene set enrichment analyses based on genome-wide DNA methylation results from model 1 are presented in ESM Tables 7–9. The insulin signalling pathway was enriched in KEGG analysis, although the association did not survive the FDR correction (FDR = 0.12). Furthermore, fatty acid and lipid homeostasis appear to be perturbed in future type 2 diabetes cases, since pathways such as phospholipid metabolism and metabolism of steroids were found to be enriched (Reactome analysis, FDR = 0.04; GO terms, FDR < 0.05). As a sensitivity analysis we repeated the gene set enrichment analyses on the fully adjusted model 2.1 (adjusted for BMI, smoking and follow-up time). As expected, similar pathways came up; however, the FDR significance level was not reached due to the higher *p* values of individual CpG sites from model 2 (ESM Tables 7–9).

Analysis of enrichment of TFs for the 65 annotated gene names out of 76 DMS, using the ChEA3 online tool, resulted in 48 TFs (*p*<0.01; ESM Table 10).

Further, we queried the list of 65 annotated gene names in the GWAS catalog to find previously reported associations of phenotypes/diseases with genetic variants at those loci. Seventeen out of 65 (26%) genes harboured genetic variant associations with at least one metabolic trait or disease, such as lipid traits, BP and obesity (Table 3; ESM Table 11).

Next, we queried the list of 76 genome-wide significant CpG sites in the EWAS catalog to find previously reported associations with phenotypes/diseases. Fifty-three out of 76 (70%) CpG sites were identified in EWAS studies of at least one metabolic trait and 24 (31.6%) CpG sites were previously reported to be associated with smoking (ESM Table 12).

We investigated whether DNA methylation levels of the 76 CpG sites were significantly associated with gene expression levels in blood. Of the 76 DMS identified, 21 CpG sites (28%) were associated with expression levels of 23 genes, including top signals at genes such as *TXNIP****,*** *ABCG1*, *SREBF1* and *CPT1A* (Table 3; ESM Table 13). Additionally, we performed a look-up of known meQTL. Of the 76 DMS, DNA methylation at 59 CpG sites (78%) showed significant association with at least one SNP and, in total, 14,813 *cis* associations were found with 13,121 SNPs (*p*<5 × 10^−8^). Of these, 80 mQTL were identified after clumping (ESM Table 14).

## Discussion

To the best of our knowledge, this is the first meta-analysis of methylation markers for incident type 2 diabetes. Previous studies have investigated the association between DNA methylation and incident type 2 diabetes in single cohorts [8, 12]. By combining DNA methylation data from five EWASs from European cohorts we successfully increased the power of the study and identified 76 DMS that were associated with incident type 2 diabetes.

Type 2 diabetes is a complex disease that exhibits metabolic changes many years prior to clinical disease onset. Using a prospective study design, we identified multiple changes in DNA methylation levels preceding the onset of type 2 diabetes. After adjustment for baseline BMI, we observed a large attenuation of significant CpG sites in the discovery phase. The EPIC-Norfolk study also investigated the effects of baseline BMI on their EWAS results and detected a similar reduction in the number of significant DMS [12]. However, a modest mean attenuation of effect sizes after BMI adjustment of 22% and the strong correlation of adjusted effect sizes with those of the primary discovery model (*r* = 0.983) suggested a smaller effect of BMI than might have been expected based only on the large reduction in number of genome-wide significant signals (reduction of 95%). Findings from a recent large EWAS focusing on BMI suggest that changes in DNA methylation profiles are a consequence of adiposity rather than a cause [27]. A look-up in the EWAS catalog revealed that 24 of our 76 top CpG sites were previously reported to be associated with smoking. This result is in line with the observed reduction in the number of significant DMS from 76 to 47 after adjustment for smoking (and follow-up time) and highlights the relevance of smoking, which not only impacts methylation but has also been reported as a risk factor for type 2 diabetes [34]. Our results show the importance of confounders such as smoking and BMI in the association between DNA methylation and type 2 diabetes. Although after adjustment for BMI effect sizes attenuate by about 20% and most CpGs lose genome-wide significance, attenuation is modest compared with the large reduction in the number of genome-wide significant signals, offering promise for future meta-analyses of larger size to significantly detect the DNA methylation signals predictive of incident type 2 diabetes that are independent of BMI.

Gene set enrichment and TF analyses performed to obtain better insight into biological mechanisms revealed perturbation of biological processes linked to insulin signalling, and fatty acid and lipid homeostasis. The results from our meta-analysis included CpG sites at genes that are known to be associated with type 2 diabetes, such as *TXNIP*, *ABCG1*, *SREBF1* and *CPT1A*, showing consistency between cross-sectional and longitudinal studies and also between ethnicities [9, 10, 35]. However, these findings are accompanied by 63 CpG sites novel for incident type 2 diabetes annotated to a number of genes that, at least partly, also seem to be relevant for type 2 diabetes. Examples include *OLMALINC*, *UFM1*, *LGALS3BP*, *TRIO* and *CFL2*. *OLMALINC* (oligodendrocyte maturation-associated long intergenic non-coding RNA) is a long intervening non-coding RNA that was recently reported to function as an epigenetic regulator of lipid metabolism [36]. *UFM1* (encoding ubiquitin-fold modifier 1) may play a crucial role in various cellular processes including endothelial reticulum stress-induced apoptosis of pancreatic beta cells [37]. *LGALS3BP* encodes a glycoprotein belonging to the family of galectins, which are presumed to be involved in regulating processes linked to the immune response and inflammation [38–40]. *TRIO* encodes a guanine exchange factor (trio rho guanine nucleotide exchange factor), which is a component of the Rho GTPase nucleotide cycle. Rho GTPases play a crucial role in metabolic homeostasis [41]. *CFL2* has been reported to be involved in actin remodelling required for recruitment of vesicles containing GLUT4 upon insulin stimulation [42]. Thus, this meta-analysis resulted in the identification of additional DNA methylation markers for incident type 2 diabetes. However, we also observed that a large proportion of those CpG sites have previously been identified in BMI, lipid and BP EWASs, suggesting common or related (epi)genetic mechanisms underlying those associations.

We recognise several limitations of the study presented here. First, although all cohorts excluded prevalent cases of type 2 diabetes at baseline based on a number of criteria (Table 1), this was not cross-validated by glycaemic measures in the EPIC-Norfolk and parts of the KORA2 and Doetinchem cohorts. As such we cannot exclude that some incident cases in these cohorts may have had prediabetes or even undiagnosed type 2 diabetes at baseline. However, forest plots of the 20 CpG sites showing considerable heterogeneity between studies failed to reveal consistent differences due to specific cohorts, suggesting that the high heterogeneity was not primarily driven by these cohorts. Second, we focused on whole-blood DNA methylation, which may not fully represent methylation patterns in other more metabolically relevant tissues such as adipose tissue, liver or muscle. Next, we cannot rule out the possibility of reverse causation, where the DNA methylation changes we identified are a consequence of raised blood glucose levels and adiposity rather than a cause. Gradually rising levels of blood glucose and adiposity in the years prior to clinical diagnosis of type 2 diabetes may elicit compensatory epigenetic changes, reflecting increased levels of metabolic dysregulation. We chose to correct our meta-analysis results for multiple testing using the commonly applied Bonferroni correction; however, we acknowledge that other methods would have yielded other sets of significant CpG sites (e.g. Saffari et al’s [43] cut-off of *p*<3.6 × 10^−8^ would have decreased the number of significant CpGs from 76 to 59). Additionally, if we had corrected our replication analysis either for 76 tests (i.e. Bonferroni) or the number of independent signals identified through PCA (i.e. 32), the set of replicated CpG sites would have decreased from 64 to 39 and 46, respectively. Importantly, this meta-analysis of results from multiple cohorts increased the statistical power of associations between DNA methylation and type 2 diabetes compared with previous single-cohort studies.

Taken together, this large meta-analysis of EWASs resulted in the identification of 76 DMS associated with incident type 2 diabetes. The results from the replication analysis in a cohort of Indian Asians suggest that the association between DNA methylation levels and incident type 2 diabetes is independent of ethnicity. Our data also show that BMI partly explains the association between DNA methylation and incident type 2 diabetes. Functional analyses revealed multiple biological pathways involved in fatty acid and lipid metabolism, immune response and inflammation, which partly underlie impaired glucose metabolism. Further studies are required to evaluate the relevance to other tissues and to determine whether these DMS have a causal role in type 2 diabetes development. In addition, a more detailed analysis of their biological function is warranted. Future work could assess correlations between our poly-epigenetic predictor of incident type 2 diabetes and DNA methylation-based predictors of BMI and related traits, including waist/hip ratio and per cent body fat such as those generated by McCartney et al [44]. It would also be interesting to test whether such DNA methylation-based predictors add information in prediction models over and above available phenotypic analogues.

## Supplementary information

ESM 1 (PDF 4171 kb)

## Data Availability

The datasets generated and/or analysed during the current study are available from the corresponding author on reasonable request.

## Abbreviations

DMS: Differentially methylated CpG sites
eQTM: Expression quantitative trait methylation
EWAS: Epigenome-wide association study
FDR: False discovery rate
GO: Gene Ontology
KEGG: Kyoto Encyclopedia of Genes and Genomes
meQTL: Methylation quantitative trait loci
MRS: Methylation risk score
PCA: Principal component analysis
TF: Transcription factor
