# Supplementary material for: Epigenome-wide association study of incident type 2 diabetes: a meta-analysis of five prospective European cohorts
Source: Diabetologia. 2022 Feb 15;65(5):763–76. doi: 10.1007/s00125-022-05652-2 (PMC8960572; doi:10.1007/s00125-022-05652-2)

Electronic supplementary material (ESM) to Epigenome-Wide Association Study of Incident Type 2 Diabetes: a meta-analysis of five prospective European cohorts

Content

ESM Methods 2

ESM Table 1. 10

ESM Table 2. 12

ESM Table 3 12

ESM Table 4 13

ESM Table 5 16

ESM Table 6 19

ESM Table 7 22

ESM Table 8 23

ESM Table 9 24

ESM Table 10. 26

ESM Table 11. 28

ESM Table 12 33

ESM Table 13 39

ESM Table 14 43

ESM Figure 1 47

ESM Figure 2. 49

ESM Figure 3. 50

ESM Figure 4 51

ESM Figure 5. 52

ESM Figure 6. 52

ESM Methods

General description of the cohorts and definitions of type 2 diabetes cases and controls

The Doetinchem Cohort Study is an ongoing, prospective, population-based study from Doetinchem, the Netherlands[1]. In brief, a random sample from the general population of women and men aged between 20 and 59 were selected for the first measurement round in 1987-1991. Adults who participated in the first round were invited for follow-up examinations in 1993-1997 (round 2, n=6117, mean age: 46 years), 1998-2002 (round 3, n=4918, mean age: 51 years), 2003-2007 (round 4, n=4520, mean age: 56 years), 2008-2012 (round 5, n=4018, mean age: 60 years), and 2013-2017 (round 6, n=3438, mean age: 64 years). Response rates were 75% or higher in all rounds. All participants provided an informed consent and the study was approved by the Medical Ethics Committee of the University Medical Center Utrecht.

The ESTHER study is an ongoing population-based cohort study conducted in the federal state of Saarland, Germany[2]. In brief, 9,949 older adults (50-75 years) were recruited by their general practitioners (GPs) during routine health check-ups (offered every two years to people older than 35 years in the German healthcare system) between 2000 and 2002, and followed up thereafter. During the baseline enrolment, epidemiological data were collected via a standardized self-administered questionnaire completed by participants and via additional reports from participants' GPs, and biological samples were obtained. Three subsets of ESTHER participants were selected for DNA methylation assessment in the baseline blood samples: subset I consists of 1,000 participants consecutively enrolled during the first 3 months of recruitment; subset II consists of 864 participants selected for a case-cohort design for mortality analysis[3]. Subset III was primarily selected to address cancer-related methylation signatures, consisting of 471 participants[4]. All participants provided an informed consent and the study was approved by the ethics committees of the University of Heidelberg and of the Medical Association of Saarland.

KORA (Cooperative Health Research in the Region of Augsburg) is a population-based cohort study conducted in Southern Germany[5]. In brief, the baseline surveys S3 and S4 were conducted in 1994/1995 and 1999-2001, respectively, and comprised independent samples of 4856 and 4261 subjects aged 25 to 74 years. Both cohorts were reinvestigated in the follow-up examinations F3 and F4 in 2004/2005 and 2006-2008, respectively, with 2974 and 3080 participants. Finally, there was a another follow up of F4 named FF4 in 2013/14 with 2279 participants. Two independent sub-cohorts from KORA were selected for EWAS analyses, designated as KORA1 (including KORA F4 and their FF4 follow up study) and KORA2 (including KORA S3 and S4 and their F3 and F4 follow up studies). Anthropometric variables and clinical parameters were determined at all examinations. All participants provided an informed consent and the study was approved by the ethics committee of the Bavarian Medical Association.

EPIC-Norfolk is a prospective cohort study that recruited 25,639 individuals aged between 40 and 79 years at baseline in 1993–1997[5]. The cohort was representative of the general population of England and Wales but differed in that 99.7% of the cohort were of European descent. Follow-up was censored at date of diagnosis of T2D, 31 July 2006, or date of death— whichever came first. All participants provided an informed consent and the study was approved by the local research ethics committee.

Replication of our epigenome-wide significant finding was sought in the London Life Sciences Prospective Population (LOLIPOP) study[6]. LOLIPOP is a prospective population study of Indian Asians and Europeans recruited at age 35-75 years from a list of 58 family doctors in London between 1 May 2002 and 12 September 2008. Indian Asians were selected based on information about all four grandparents born in Indian subcontinent (India, Pakistan, Sri Lanka, Bangladesh). Details of the nested case-control

study of incident T2D have been described previously[6]. The LOLIPOP study is approved by the National Research Ethics Service (07/H0712/150), and all participants gave written informed consent at enrolment.

DNA methylation quality control and normalization

In the Doetinchem Cohort Study, DNA was isolated from whole blood samples with the commonly used salting out method[7]. Next, 500 ng of genomic DNA of each sample was bisulfite converted using the EZ DNA Methylation kit (Zymo Research, Irvine, California, USA) and hybridized to Illumina Infinium Methylation EPIC arrays according to the manufacturer's protocols. The original IDAT files were generated by the Illumina iScan BeadChip scanner. Data was generated by the Genome Analysis Facility of the UMCG, the Netherlands (www.rug.nl/research/genetics/genomeanalysisfacility/). Preprocessing and quality control of the methylation data was done according to the minfi tutorial[8]. Probes containing
a SNP in the sequence, sex probes and probes with detection p-value>0.001 in more than 5% of samples were removed. Subsequently, quantile normalization was applied using limma[9]. Adjustment for batch effects was done using ComBat[10, 11]. The Illumina Infinium Methylation EPIC array from Illumina includes approximately 90% of the probes from the Infinium HumanMethylation450K array. We used the HumanMethylation450 v1.2 Manifest File [12] to select the 450K probes from the EPIC array, which resulted in 424,748 probes to be used for this particular study.

In ESTHER, DNA methylation in whole blood was quantified using the Infinium HumanMethylation450K BeadChip (Illumina.Inc, San Diego, CA, USA). In brief, 1.5 mg DNA (allocated in 96-well format with three random duplicate samples in each format as quality controls) was bisulfite converted, and 200 ng bisulfite-treated DNA was applied to the 450K BeadChips following the manufacturer's instruction. Raw data pre-processing and initial quality control was carried out following the CPACOR pipeline[13]. Probes with detection p-value>0.01 were removed before quantile normalization, which was applied following stratification of the probe type into 6 categories according to probe type and color channel, using the R package limma[9]. Sample call rate threshold and CpG call rate threshold both were 95%. A principal component analysis (PCA) was performed for the positive control probes, and the first 30 control probe PCAs were included in the regression model for batch correction.

In KORA1 and KORA2 studies methylation was quantified in bisulfite converted genomic DNA from whole blood, using the Illumina Infinium HumanMethylation450 array in all samples. Quality control was performed using the minfi package[8] and included removal of the probes if detection *p*-value was higher than 0.01 and the sample and marker call rate thresholds were set at 95%. Quantile normalization of intensity values separated into 6 categories was applied. A principle component analysis (PCA) was performed for the positive control probes, and the first 30 control probe PCAs were included in the regression model for batch correction.

In EPIC-Norfolk, epigenome-wide DNA methylation data were analyzed in R (version 3.2.2). Methylation intensity values were corrected using the Illumina background correction algorithm as implemented in minfi[8], methylation intensities with a detection p-value ≥ 0.01 were set to "missing," and methylation intensity beta values were calculated for each methylation marker per sample. Details of the quality control were previously described[14].

In LOLIPOP, DNA methylation data were analyzed in R (version 2.15) using minfi[8] and other R scripts. Marker intensities were normalized by quantile normalization. Details of the quality control were previously described[6].

Since presence of extreme outlying values in DNA methylation data (< 25th percentile− 3*IQR (interquartile range) or > 75th percentile +3*IQR)) may have a great impact on EWAS results, such probes (i.e., CpGs) were removed prior to analysis if identified in more than 20% of samples. All other extreme outliers identified in this way were set to "missing"[15]. The leukocyte composition (CD4 and CD8 T-cell subtypes, natural killer cells, monocytes, granulocytes, and B cells) of the samples were estimated using Houseman's algorithm[11]. The resulting cell count estimates and cohort-specific DNA methylation batches were then used as covariates in all regression models for incident type 2 diabetes.

Analysis plan for all cohorts

1. DNA methylation Quality control and Normalization

Pre-processing may differ per cohort, and should be done before the EWAS. Specific details regarding pre-processing, excluded samples and probes, method of normalization and a short description about the cohort itself (cohort design, inclusion and exclusion criteria etc) should be included in the Study_Summary Excel file and uploaded together with other results.

Normalized beta-values will be used as outcome variable. Please use trimming method to get rid of outliers as proposed by the Pregnancy And Childhood Epigenetics (PACE) consortium (see code below). Remove probes if outliers were detected in >20% of samples (see code below).

2. Phenotype and covariates

The phenotype of interest is incident type 2 diabetes – modelled as a binary variable (0 – controls, 1- incident type 2 diabetes). Preferably, type 2 diabetes diagnosis should be based on the WHO definition from 2006 report (fasting glucose >7.0 and/or HbA1c >6.5% and/or 2-h plasma glucose>11.1 mmol/l) OR self-reported and verified by the General Practitioner (as in Doetinchem Cohort). Controls should be healthy, with no family history of T2D, no gestational diabetes and should be all normogylcemic at any time of follow-up (defined by WHO, i.e. random blood glucose <7.8 mmol/L and/or fasting plasma glucose <6.1mmol/l and/or HbA1c<5.6%). Controls should be matched to cases based on sex, age (+/- 1.0 years) and measurement wave (if applicable).

Covariates:

- Sex (0-male, 1 –female)
- Age (years) at baseline (i.e., when blood was collected for EWAS)
- BMI (kg/m2) at baseline (i.e., when blood was collected for EWAS)
- Batch: Adjustment for batch is essential in array based methylation studies. Either adjusting the model for plate, chip etc OR for Principal Component Scores OR using batch corrections such as ComBat. Please state clearly which method(s) were used for your cohort in Study_Summary Excel file
- Cell type: Please include cell proportions estimated by the Houseman method in all models; this covariate should be included additively in the model (first Granulocytes, then Nk cells etc).
- Time (in years):

- for T2D cases - the time period between blood sample collection and the year of T2D diagnosis;

- for controls – the time period between blood sample collection and the year of matching of control to case (i.e. in the Doetinchem cohort sample, this variable was calculated based on the year of the follow-up measurements and data collection round when control was matched to case minus the year of blood sample collection)

• Smoking (0- never smoker, 1-current smoker, 2-ex smoker)

3. Models:

linear regression modelling (rlm() option in R)

Model 1:

Methylation values (normalized betas[0:1])~ Diabetes (0,1)+sex +age +Cell types+ Batches

Model 1.1 (sensitivity analysis):

Methylation values (normalized betas[0:1])~ Diabetes (0,1)+sex+age+Cell types+Batches + time+smoking

Model 2 adjusted for BMI:

Methylation values (normalized betas[0:1])~ Diabetes (0,1)+sex +age +BMI +Cell types+ Batches

Model 2.1 adjusted for BMI (sensitivity analysis):

Methylation values (normalized betas[0:1])~ Diabetes (0,1)+sex +age +BMI +Cell types+ Batches+Time+Smoking

Reporting results

Please provide 4 separate files: (see code below)

1. [Cohort_Model1_date.txt] Results of EWAS model 1 as .txt file with columns corresponding to

• CpG (“CpG”), Coefficient (“coef”), SE (“se”), and p-value (“pvalue”).

2. [Cohort_Model2_date.txt] Results of EWAS model 2 as .txt file with columns corresponding to

• CpG (“CpG”), Coefficient (“coef”), SE (“se”), and p-value (“pvalue”).

3. [Cohort_descriptives_methylation_date.txt] The table containing summary statistics should include for each CpG the minimum, 10% quantile, 25% quantile, median, mean, sd, 75% quantile, 90% quantile, maximum, and number of missing samples.

4. [Cohort_Study_Summary_date.xlsx] Excel Study_Summary with 3 Tables:

• Table 1. Baseline characteristics of each cohort.

• Table 2. QC and normalization of methylation data.

• Table 3. Lambdas and number of samples per model.

Example R code (based on code used by the PACE consortium):

EWAS:

```
# libraries
library(data.table)# to process results
library(MASS) # rlm function for robust linear regression
library(sandwich) #Huberís estimation of the standard error
library(lmtest) # to use coeftest
library(parallel) # to use multicore approach - part of base R
# load methylation data as beta values, probe annotation data, phenotype data, batch
data
# make sure that phenotype data is being treated in the correct way by R (e.g. factors
for categorical data, with the first level of the factor being the reference or
baseline category)
# match data, e.g.:
# match methylation beta-value matrix with phenotype data
PHENO <- pheno_data[match(as.numeric(colnames(beta_matrix)),pheno_data[,1]),]
if (mean(as.numeric(pheno_data[,1]) - as.numeric(colnames(beta_matrix)), na.rm = T) ==
0) {
print("matched phenotype data")
}
#transpose betas so that rows are samples and columns are probes
beta_matrix<-t(beta_matrix)
#Add function for running the model (in this example, there are 4 covariates in
addition to batch - any amendments to this must be made in the function and also when
it is called below)
RLMtest = function(meth_matrix,methcol,exposure, X1, X2, X3, X4,batch) {
mod = try(rlm(meth_matrix[, methcol]~exposure+X1+X2+X3+X4+batch,maxit=200))
if(class(mod) == "try-error"){
print(paste("error thrown by column", methcol))
invisible(rep(NA, 3))
}else cf = coeftest(mod, vcov=vcovHC(mod, type="HC0"))
cf[2, c("Estimate", "Std. Error", "Pr(>|z|)")]
}
#Run adjusted EWAS
system.time(ind.res <- mclapply(setNames(seq_len(ncol(beta_matrix)),
dimnames(beta_matrix)[[2]]), RLMtest, meth_matrix=beta_matrix, exposure=PHENO[,2],
X1=PHENO[,3], X2=PHENO[,4], X3=PHENO[,5], X4=PHENO[,6], batch=PHENO$batch))
#Process results
setattr(ind.res, 'class', 'data.frame')
setattr(ind.res, "row.names", c(NA_integer_,4))
setattr(ind.res, "names", make.names(names(ind.res), unique=TRUE))
probelistnames <- names(ind.res)
all.results <- t(data.table(ind.res))
all.results<-data.table(all.results)
all.results[, probeID := probelistnames]
setnames(all.results, c("BETA","SE", "P_VAL", "probeID")) # rename columns
setcolorder(all.results, c("probeID","BETA","SE", "P_VAL"))
rm(probelistnames, ind.res)
# export table of results
write.table(all.results, " GA_TYPE_CELL_STUDY_DATE.txt.",na="NA")
gzip("GA_TYPE_CELL_STUDY_DATE.txt")
##### Calculate lambda
lambda <- qchisq(median(all.results$P_VAL,na.rm=T), df = 1, lower.tail = F)/
qchisq(0.5, 1)
## Save analysis sample sizes & lambda:
N<-dim(tdat)[1]
N_Preterm<-sum(Preterm==1)
save(N,N_Preterm,lambda,file="BAMSE_model1_SampleSizeLambda.RData")
```

Summary_probes

```
# SUMMARIZE PROBES
descriptives<-function(x){
```

```
tmp<-c(min(x,na.rm=T),quantile(x,probs=c(.1,.25,.5),na.rm=T),mean(x,na.rm=T),
median(x,na.rm=T),sd(x,na.rm=T),quantile(x,probs=c(.75,.90),na.rm=T),max(x,na.rm=T),sum
(is.na(x)))
names(tmp)[c(1,5:7,10:11)]<-c("Min.","Mean","Median","SD","Max.","NA")
return(tmp)
}
desc<-t(apply(beta_matrix,2,descriptives))
write.table(desc, file = paste(outdir,"/BAMSE_Descriptives.txt", sep = ""),
sep = "\t", col.names = T, row.names = T, append = F, quote=FALSE)
```

Trimming outliers

```
#################### Code for trimming###############################
Code for trimming methylation beta values to remove potential outliers
Trimming scheme is as follows – trim values beyond the lower and upper outer fences.
These are defined by:
Values < 25th percentile minus 3*IQR AND Values > 75th percentile plus 3*IQR
(IQR = interquartile range)
#Function
removeOutliers<-function(probes){
require(matrixStats)
if(nrow(probes) < ncol(probes)) warning("expecting probes are rows (long dataset)")
rowIQR <- rowIQRs(probes, na.rm = T)
row2575 <- rowQuantiles(probes, probs = c(0.25, 0.75), na.rm = T)
maskL <- probes < row2575[,1] - 3 * rowIQR
maskU <- probes > row2575[,2] + 3 * rowIQR
initial_NAs<-rowSums(is.na(probes))
probes[maskL] <- NA
removed_lower <- rowSums(is.na(probes))-initial_NAs
probes[maskU] <- NA
removed_upper <- rowSums(is.na(probes))-removed_lower-initial_NAs
N_for_probe<-rowSums(!is.na(probes))
Log<-data.frame(initial_NAs,removed_lower,removed_upper,N_for_probe)
return(list(probes, Log))
```

Prediction models in Doetinchem Cohort Study

To calculate methylation risk scores based on different p-value thresholds, we first performed leave-one-out meta-analysis without Doetinchem Cohort Study. We then used the beta coefficients of this meta-analysis results as weights in calculating MRS. We included 4 p-value thresholds to investigate the predictive ability of CpG sites identified at less stringent p-value ($1x10^{-7}$, $1x10^{-6}$, $1x10^{-5}$, $1x10^{-4}$). First we compared the model M1 including age, sex, BMI, cell types and batch with model M2 including additionally MRS build on genome-wide significant CpG sites only. Next, we included 3 other MRS in model M2 and compared the AUC between those models.

References

1. Verschuren W, Blokstra A, Picavet H, Smit H (2008) Cohort Profile: The Doetinchem Cohort Study. Int J Epidemiol 37(6):1236–1241. https://doi.org/10.1093/ije/dym292

2. Raum E, Rothenbacher D, Löw M, Stegmaier C, Ziegler H, Brenner H (2007) Changes of cardiovascular risk factors and their implications in subsequent birth cohorts of older adults in Germany: a life course approach. Eur J Cardiovasc Prev Rehabil 14(6):809–814. https://doi.org/10.1097/HJR.0b013e3282eeb308

3. Zhang Y, Wilson R, Heiss J, et al (2017) DNA methylation signatures in peripheral blood strongly predict all-cause mortality. Nat Commun 8:14617. https://doi.org/10.1038/ncomms14617

4. Zhang Y, Saum K-U, Schöttker B, Holleczek B, Brenner H (2018) Methylomic survival predictors, frailty, and mortality. Aging (Albany NY) 10(3):339–357. https://doi.org/10.18632/aging.101392

5. Day N, Oakes S, Luben R, et al (1999) EPIC-Norfolk: study design and characteristics of the cohort. European Prospective Investigation of Cancer. Br J Cancer 80 Suppl 1:95–103

6. Chambers JC, Loh M, Lehne B, et al (2015) Epigenome-wide association of DNA methylation markers in peripheral blood from Indian Asians and Europeans with incident type 2 diabetes: a nested case-control study. Lancet Diabetes Endocrinol 3(7):526–534. https://doi.org/10.1016/S2213-8587(15)00127-8

7. Miller SA, Dykes DD, Polesky HF (1988) A simple salting out procedure for extracting DNA from human nucleated cells. Nucleic Acids Res 16(3):1215

8. Aryee MJ, Jaffe AE, Corrada-Bravo H, et al (2014) Minfi: a flexible and comprehensive Bioconductor package for the analysis of Infinium DNA methylation microarrays. Bioinformatics 30(10):1363–1369. https://doi.org/10.1093/bioinformatics/btu049

9. Ritchie ME, Phipson B, Wu D, et al (2015) limma powers differential expression analyses for RNA-sequencing and microarray studies. Nucleic Acids Res 43(7):e47–e47. https://doi.org/10.1093/nar/gkv007

10. Johnson WE, Li C, Rabinovic A (2007) Adjusting batch effects in microarray expression data using empirical Bayes methods. Biostatistics 8(1):118–127. https://doi.org/10.1093/biostatistics/kxj037

11. Houseman EA, Accomando WP, Koestler DC, et al (2012) DNA methylation arrays as surrogate measures of cell mixture distribution. BMC Bioinformatics 13:86. https://doi.org/10.1186/1471-2105-13-86 [doi]

12. HumanMethylation450 v1.2 Manifest File (https://support.illumina.com/downloads/infinium_humanmethylation450_product_files.html)

13. Lehne B, Drong AW, Loh M, et al (2015) A coherent approach for analysis of the Illumina HumanMethylation450 BeadChip improves data quality and performance in epigenome-wide association studies. Genome Biol 16(1):37. https://doi.org/10.1186/s13059-015-0600-x

14. Cardona A, Day FR, Perry JRB, et al (2019) Epigenome-Wide Association Study of Incident Type 2 Diabetes in a British Population: EPIC-Norfolk Study. Diabetes db180290. https://doi.org/10.2337/db18-0290

15. Sharp GC, Arathimos R, Reese SE, et al (2018) Maternal alcohol consumption and offspring DNA methylation: findings from six general population-based birth cohorts. Epigenomics 10(1):27–42. https://doi.org/10.2217/epi-2017-0095

ESM Table 1. List of significant CpG sites associated with incident T2D after additional adjustment for smoking and follow-up time in five European cohorts (N =3200) with replication results from LOLIPOP cohort (N=2659).

Illumina ID	Gene name	CHR	Position	Effect size	Standard Error	HetISq	HetPVal	P.value	FDR	Direction across studies[a]	Effect size replication	P.value replication
cg19693031	*TXNIP*	1	145441552	-0.0199	0.002	87,8	1,347E-06	**3.89E-23**	1.84E-17	-----	-0.0119	**1.15E-12**
cg06500161	*ABCG1*	21	43656587	0.0109	0.0011	63,2	0,028	**9.26E-22**	2.19E-16	+++++	0.0092	**7.58E-20**
cg11024682*	*SREBF1*	17	17730094	0.0088	0.0011	42,7	0,137	**1.41E-14**	2.23E-09	+++++	0.0075	**1.28E-13**
cg00574958	*CPT1A*	11	68607622	-0.0051	0.0007	80,5	0,000	**6.35E-13**	7.52E-08	-----	-0.0037	**1.99E-07**
cg05778424	*AKAP1*	17	55169508	0.0075	0.0011	65	0,022	**3.56E-11**	3.37E-06	+++++	0.0043	**7.07E-06**
cg14020176*	*SLC9A3R1*	17	72764985	0.0087	0.0014	33,9	0,209	**1.53E-10**	1.21E-05	+?+++	0.0033	**3.63E-04**
cg07504977	*OLMALINC*	10	102131012	0.0112	0.0018	0	0,716	**2.17E-10**	1.47E-05	+++++	0.0062	**1.37E-04**
cg04816311	*C7orf50*	7	1066650	0.0112	0.0018	66,5	0,018	**2.76E-10**	1.6E-05	+++++	0.0059	**1.72E-03**
cg14476101	*PHGDH*	1	120255992	-0.0145	0.0023	59,9	0,041	**3.04E-10**	1.6E-05	-----	-0.0100	**2.15E-06**
cg27243685	*ABCG1*	21	43642366	0.0061	0.001	0	0,801	**4.26E-10**	2.02E-05	+++++	0.0052	**8.45E-13**
cg14870271*	*LGALS3BP*	17	76976010	0.0084	0.0014	39	0,161	**5E-10**	2.15E-05	+++++	0.0056	**4.98E-05**
cg06397161	*SYNGR1*	22	39760059	0.0093	0.0015	59,5	0,060	**6.5E-10**	2.56E-05	?++++	0.0057	**1.01E-04**
cg06940720	*LPCAT1*	5	1526929	0.007	0.0011	0	0,522	**7.58E-10**	2.76E-05	+++++	0.0039	**1.19E-04**
cg26262157	*PFKFB3*	10	6214079	-0.009	0.0015	63,9	0,026	**1.03E-09**	3.47E-05	-----	-0.0054	**1.05E-04**
cg18568872	*ZNF710*	15	90606494	0.006	0.001	0	0,587	**1.22E-09**	3.84E-05	+++++	0.0029	**3.69E-04**
cg01373896	*KLF16*	19	1854724	0.0068	0.0011	50,1	0,091	**1.3E-09**	3.84E-05	+++++	0.0020	5.45E-02
cg08994060	*PFKFB3*	10	6214026	-0.0107	0.0018	62	0,032	**1.66E-09**	4.46E-05	-----	-0.0056	**1.11E-03**
cg11202345	*LGALS3BP*	17	76976057	0.0078	0.0013	0	0,496	**1.7E-09**	4.46E-05	+++++	0.0062	**6.55E-06**
cg21480264	*POLN*	4	2137264	0.0059	0.001	0	0,826	**2.45E-09**	6.12E-05	+++++	0.0025	**2.73E-03**
cg19750657	*UFM1*	13	38935967	0.0089	0.0015	15,7	0,314	**2.66E-09**	6.16E-05	+++++	0.0065	**7.09E-07**
cg06192883	*MYO5C*	15	52554171	0.008	0.0013	80,5	3,95E-04	**2.73E-09**	6.16E-05	+++++	0.0066	**4.53E-06**
cg21234053*	*CFL2*	14	35163420	0.0156	0.0026	38,9	0,195	**2.98E-09**	6.41E-05	??+++	0.0051	**2.27E-03**
cg06378491	*MAP4K2*	11	64564012	0.0044	0.0008	47,1	0,109	**3.45E-09**	7.11E-05	+++++	0.0025	**9.22E-05**

cg12257439	*FER1L5*	2	97360893	0.0053	0.0009	40,2	0,153	**4.07E-09**	7.94E-05	+++++	0.0030	**7.53E-05**
cg11269166	*METTL8*	2	172203847	0.0066	0.0011	80,9	3,28E-04	**4.19E-09**	7.94E-05	+++++	0.0025	**2.31E-02**
cg08788930	*DENND3*	8	142201685	0.0073	0.0013	37,7	0,170	**5.83E-09**	0.000106	+++++	0.0034	**1.52E-03**
cg22650271	*SYNGR1*	22	39760165	0.0057	0.001	72,4	0,006	**6.62E-09**	0.000116	+++++	0.0034	**4.62E-04**
cg03691549	*TENC1*	12	53443911	0.0059	0.001	0	0,674	**9.51E-09**	0.000161	+++++	0.0026	**8.53E-03**
cg09664445	*KIAA0664*	17	2612406	0.0054	0.0009	37,6	0,171	**1.03E-08**	0.000163	+++++	0.0021	**1.48E-02**
cg02711608*	*SLC1A5*	19	47287964	-0.0087	0.0015	0	0,376	**1.03E-08**	0.000163	??---	-0.0035	**1.14E-03**
cg14956201	*TRIO*	5	14358153	0.0077	0.0014	0	0,493	**1.11E-08**	0.00017	+++++	0.0013	3.24E-01
cg17540192	*TECPR1*	7	97875259	0.0048	0.0008	71,4	0,007	**1.47E-08**	0.000218	+++++	0.0027	**1.28E-03**
cg25217710	*BCAN*	1	156609523	0.0052	0.0009	67,6	0,015	**1.52E-08**	0.000218	+++++	0.0027	**2.97E-04**
cg16861241	*FOXJ1*	17	74138396	0.0056	0.001	0	0,782	**1.65E-08**	0.00023	+++++	0.0005	5.84E-01
cg15020801	*PNPO*	17	46022809	0.007	0.0012	40,9	0,149	**1.8E-08**	0.000244	+++++	0.0060	**4.95E-07**
cg02879453*	*ADCY7*	16	50321818	0.0078	0.0014	50,8	0,087	**3.99E-08**	0.000525	+++++	0.0054	**1.17E-05**
cg04682775	*SLC6A9*	1	44495089	0.0066	0.0012	8,3	0,359	**4.17E-08**	0.000534	+++++	0.0011	2.55E-01
cg10639435*	*ZNF250*	8	146104221	0.0077	0.0014	0	0,745	**4.28E-08**	0.000534	+++++	0.0014	3.06E-01
cg04927537	*LGALS3BP*	17	76976091	0.0103	0.0019	0	0,587	**4.87E-08**	0.000592	+++++	0.0063	**4.68E-04**
cg16097041	*FLAD1*	1	154965544	0.0061	0.0011	0	0,509	**5.05E-08**	0.000599	+++++	0.0031	**7.41E-04**
cg25130381	*SLC9A1*	1	27440721	0.0057	0.001	55,7	0,060	**5.81E-08**	0.000671	+++++	0.0049	**6.85E-07**
cg20507228*	*MAN2A2*	15	91460071	0.0124	0.0023	0	0,809	**6.37E-08**	0.000719	+?+++	0.0048	**2.12E-03**
cg05460226	*PIK3R5*	17	8804279	0.0095	0.0018	15,2	0,318	**6.92E-08**	0.000763	+++++	NA	NA
cg01101459	*LINC01132*	1	234871477	0.0065	0.0012	74,5	0,003	**7.45E-08**	0.000802	+++++	0.0086	**1.59E-09**
cg25178683	*LGALS3BP*	17	76976267	0.0083	0.0015	0	0,471	**8.06E-08**	0.000848	+++++	0.0059	**4.22E-05**
cg09072148*	*NRXN2*	11	64491639	0.0037	0.0007	24,1	0,261	**9.11E-08**	0.000939	+++++	0.0004	4.50E-01
cg12322877	*ASPSCR1*	17	79963213	0.0119	0.0022	71,4	0,007	**9.7E-08**	0.000978	-++++	0.0062	**2.97E-03**

*polymorphic probe ; p-values<0.05 in bold; [a] Order of the studies – Doetinchem, ESTHER, KORA1, KORA2, EPIC-Norfolk;

ESM Table 2. List of significant CpG sites associated with incident T2D after adjustment for BMI in five European cohorts (N =3200) with replication results from LOLIPOP cohort (N=2659).

Illumina ID	Gene name	CHR	Position	Effect size	Standard Error	HetISq	HetPVal	P.value	FDR	Direction across studies[a]	Effect size replication	P.value replication
cg19693031	*TXNIP*	1	1.45E+08	-0.0183	0.0021	85.7	1.3E-06	6.1E-19	2.89E-13	-----	-0.0106	**5.78E-10**
cg06500161	*ABCG1*	21	43656587	0.0075	0.0012	55.5	0.061	8.79E-11	2.08E-05	+++++	0.0070	**6.53E-12**
cg21234053	*CFL2*	14	35163420	0.0159	0.0028	30.2	0.239	1.41E-08	0.002225	+?++?	0.0034	**4.86E-02**
cg14956201	*TRIO*	5	14358153	0.0074	0.0014	0	0.733	8.58E-08	0.010158	+++++	0.0005	6.95E-01

p-values replication <0.05 in bold; [a] Order of the studies – Doetinchem, ESTHER, KORA1, KORA2, EPIC-Norfolk;

ESM Table 3. List of significant CpG sites associated with incident T2D after adjustment for BMI, smoking and follow-up time in five European cohorts (N =3200) with replication results from LOLIPOP cohort (N=2659).

Illumina ID	Gene name	CHR	Position	Effect size	Standard Error	HetISq	HetPVal	P.value	FDR	Direction across studies[a]	Effect size replication	P.value replication
cg19693031	*TXNIP*	1	1.45E+08	-0.0185	0.0021	83.4	76.6E-05	1.62E-18	7.69E-13	-----	-0.0105	**1.19E-09**
cg06500161	*ABCG1*	21	43656587	0.0076	0.0012	38.9	0.162	2.41E-10	5.7E-05	+++++	0.0070	**9.56E-12**
cg21234053	*CFL2*	14	35163420	0.0159	0.0029	21.8	0.278	4.06E-08	0.006416	+?++?	0.0032	6.46E-02

p-values replication <0.05 in bold; [a] Order of the studies – Doetinchem, ESTHER, KORA1, KORA2, EPIC-Norfolk;

ESM Table 4. Attenuation of effect sizes (% change) between different models in discovery meta-EWAS and between discovery and replication for 76 significant CpG sites associated with incident T2D.

Illumina ID	Gene name	Effect_M1	Effect_M1.1	Effect_M2	Effect_M2.1	Effect_REP*	M1 M1.1 %	M1 M2%	M1 M2.1 %	M1 DISC REP %
cg19693031	*TXNIP*	-0.0198	-0.0199	-0.0183	-0.0185	-0,0120	1%	-8%	-7%	-39%
cg06500161	*ABCG1*	0.0111	0.0109	0.0075	0.0076	0,0092	-2%	-32%	-32%	-17%
cg11024682	*SREBF1*	0.0094	0.0088	0.0059	0.0053	0,0076	-6%	-37%	-44%	-19%
cg00574958	*CPT1A*	-0.0053	-0.0051	-0.0032	-0.0031	-0,0037	-4%	-40%	-42%	-30%
cg05778424	*AKAP1*	0.008	0.0075	0.0062	0.0056	0,0042	-6%	-23%	-30%	-48%
cg14476101	*PHGDH*	-0.0151	-0.0145	-0.0099	-0.0093	-0,0101	-4%	-34%	-38%	-33%
cg04816311	*C7orf50*	0.0118	0.0112	0.0096	0.0089	0,0058	-5%	-19%	-25%	-51%
cg07504977		0.0114	0.0112	0.0084	0.0083	0,0066	-2%	-26%	-27%	-42%
cg19750657	*UFM1*	0.0096	0.0089	0.006	0.0053	0,0065	-7%	-38%	-45%	-32%
cg06378491	*MAP4K2*	0.0047	0.0044	0.004	0.0036	0,0025	-6%	-15%	-23%	-46%
cg14020176	*SLC9A3R1*	0.0087	0.0087	0.0067	0.0068	0,0033	0%	-23%	-22%	-63%
cg06397161	*SYNGR1*	0.0095	0.0093	0.0074	0.0073	0,0055	-2%	-22%	-23%	-42%
cg06940720		0.0072	0.007	0.0056	0.0052	0,0040	-3%	-22%	-28%	-44%
cg02711608	*SLC1A5*	-0.0094	-0.0087	-0.0068	-0.0059	-0,0037	-7%	-28%	-37%	-61%
cg06192883	*MYO5C*	0.0085	0.008	0.006	0.0059	0,0065	-6%	-29%	-31%	-24%
cg09664445	*KIAA0664*	0.0059	0.0054	0.0043	0.004	0,0020	-8%	-27%	-32%	-67%
cg14870271	*LGALS3BP*	0.0084	0.0084	0.006	0.0058	0,0057	0%	-29%	-31%	-33%
cg18568872	*ZNF710*	0.006	0.006	0.0044	0.0046	0,0029	0%	-27%	-23%	-52%
cg12257439	*FER1L5*	0.0055	0.0053	0.0046	0.0042	0,0032	-4%	-16%	-24%	-43%
cg11269166	*METTL8*	0.0068	0.0066	0.0057	0.0054	0,0026	-3%	-16%	-21%	-62%
cg14956201	*TRIO*	0.0082	0.0077	0.0074	0.0067	0,0014	-6%	-10%	-18%	-83%
cg17540192	*TECPR1*	0.0051	0.0048	0.0043	0.0039	0,0028	-6%	-16%	-24%	-44%
cg27243685	*ABCG1*	0.006	0.0061	0.0038	0.0041	0,0053	2%	-37%	-32%	-12%
cg11202345	*LGALS3BP*	0.0078	0.0078	0.0056	0.0057	0,0063	0%	-28%	-27%	-19%

cg15020801	*PNPO*	0.0073	0.007	0.0058	0.0053	0,0061	-4%	-21%	-27%	-17%
cg21480264	*POLN*	0.0059	0.0059	0.0053	0.0049	0,0026	0%	-10%	-17%	-55%
cg08788930	*DENND3*	0.0074	0.0073	0.0059	0.0056	0,0035	-1%	-20%	-24%	-53%
cg25217710		0.0054	0.0052	0.004	0.0038	0,0026	-4%	-26%	-30%	-52%
cg22650271	*SYNGR1*	0.0056	0.0057	0.0035	0.0037	0,0032	2%	-38%	-34%	-43%
cg10639435	*ZNF250*	0.008	0.0077	0.0074	0.007	0,0016	-4%	-8%	-13%	-80%
cg01101459		0.007	0.0065	0.0058	0.005	0,0087	-7%	-17%	-29%	25%
cg03691549	*TENC1*	0.0059	0.0059	0.0046	0.0047	0,0026	0%	-22%	-20%	-56%
cg26262157	*PFKFB3*	-0.0084	-0.009	-0.0067	-0.0075	-0,0053	7%	-20%	-11%	-36%
cg04927537	*LGALS3BP*	0.0107	0.0103	0.0064	0.0057	0,0064	-4%	-40%	-47%	-40%
cg08994060	*PFKFB3*	-0.0101	-0.0107	-0.0084	-0.0093	-0,0056	6%	-17%	-8%	-44%
cg13059136	*SNORA54*	0.008	0.0074	0.0059	0.0051	0,0049	-8%	-26%	-36%	-39%
cg21234053		0.015	0.0156	0.0159	0.0159	0,0052	4%	6%	6%	-65%
cg08309687		-0.0112	-0.0106	-0.0066	-0.0062	-0,0067	-5%	-41%	-45%	-41%
cg02879453	*ADCY7*	0.008	0.0078	0.0061	0.0062	0,0054	-3%	-24%	-23%	-33%
cg24259291	*ZNFX1*	0.0046	0.0042	0.0041	0.0037	0,0006	-9%	-11%	-20%	-88%
cg26846781	*KCNH6*	0.0045	0.0043	0.0034	0.0032	0,0036	-4%	-24%	-29%	-20%
cg16097041	*FLAD1*	0.0061	0.0061	0.0039	0.004	0,0031	0%	-36%	-34%	-50%
cg01373896	*KLF16*	0.0062	0.0068	0.0047	0.0052	0,0018	10%	-24%	-16%	-71%
cg19169154	*MFAP4*	0.0049	0.0044	0.0038	0.0033	0,0011	-10%	-22%	-33%	-78%
cg13300580	*SLC9A1*	0.0047	0.0044	0.0038	0.0035	0,0047	-6%	-19%	-26%	1%
cg23021329	*TLR9*	0.0051	0.0046	0.0043	0.0038	0,0019	-10%	-16%	-25%	-63%
cg25001190	*NFIA*	-0.01	-0.0094	-0.0087	-0.0082	-0,0043	-6%	-13%	-18%	-57%
cg02050917	*SKI*	0.0069	0.0066	0.0051	0.0048	0,0023	-4%	-26%	-30%	-67%
cg07719604	*ELMO3*	0.0074	0.0069	0.0051	0.0046	0,0044	-7%	-31%	-38%	-41%
cg26663590		0.0083	0.0078	0.0067	0.0061	0,0035	-6%	-19%	-27%	-58%
cg17836612	*LGALS3BP*	0.0063	0.0057	0.0053	0.0047	0,0039	-10%	-16%	-25%	-38%
cg20507228	*MAN2A2*	0.0126	0.0124	0.0103	0.0102	0,0048	-2%	-18%	-19%	-62%
cg04682775	*SLC6A9*	0.0065	0.0066	0.0049	0.0051	0,0011	2%	-25%	-22%	-83%
cg24145109		0.0152	0.0143	0.0127	0.0114	0,0040	-6%	-16%	-25%	-74%

cg10192877	*ABCG1*	0.0038	0.0037	0.0025	0.0024	0,0020	-3%	-34%	-37%	-47%
cg21703988	*EP400*	0.005	0.0047	0.0049	0.0043	0,0021	-6%	-2%	-14%	-59%
cg17901584	*DHCR24*	-0.0093	-0.0087	-0.0066	-0.0058	-0,0096	-6%	-29%	-38%	3%
cg25178683	*LGALS3BP*	0.0084	0.0083	0.0057	0.0054	0,0061	-1%	-32%	-36%	-28%
cg25130381	*SLC9A1*	0.0056	0.0057	0.004	0.0039	0,0050	2%	-29%	-30%	-10%
cg25649826	*USP22*	0.0058	0.0056	0.0039	0.0037	0,0018	-3%	-33%	-36%	-69%
cg20212624	*CNP*	0.0067	0.0061	0.0059	0.0052	0,0030	-9%	-12%	-22%	-55%
cg07567724	*GATAD2B*	0.0076	0.0073	0.0067	0.0063	0,0011	-4%	-12%	-17%	-86%
cg16861241	*FOXJ1*	0.0052	0.0056	0.0034	0.0041	0,0004	8%	-35%	-21%	-93%
cg03819286	*MGRN1*	0.006	0.0054	0.0049	0.0041	0,0031	-10%	-18%	-32%	-49%
cg02079413	*SNORA54*	0.0073	0.0069	0.0056	0.005	0,0059	-5%	-23%	-32%	-19%
cg23722778	*ENPP4*	-0.0086	-0.0083	-0.0088	-0.0086	-0,0009	-3%	2%	0%	-90%
cg11800635	*DOK1*	0.0088	0.0091	0.0063	0.0066	0,0053	3%	-28%	-25%	-40%
cg25316512	*ATN1*	0.0045	0.004	0.004	0.0033	0,0021	-11%	-11%	-27%	-54%
cg09294084	*MCF2L*	0.0107	0.0094	0.0093	0.008	0,0062	-12%	-13%	-25%	-42%
cg20784591	*PILRA*	0.0041	0.0036	0.0035	0.003	0,0025	-12%	-15%	-27%	-38%
cg03497652	*ANKS3*	0.0085	0.0079	0.0071	0.0063	0,0056	-7%	-16%	-26%	-34%
cg24678869	*DENND4B*	0.0042	0.004	0.003	0.0029	0,0029	-5%	-29%	-31%	-32%
cg12322877	*ASPSCR1*	0.0115	0.0119	0.0085	0.0094	0,0057	3%	-26%	-18%	-51%
cg09072148	*NRXN2*	0.0036	0.0037	0.0037	0.0037	0,0005	3%	3%	3%	-87%
cg14524754	*B3GNTL1*	0.0069	0.0065	0.0072	0.0066	0,0025	-6%	4%	-4%	-64%
cg17194270	*SYNGR1*	0.0092	0.009	0.0051	0.0052	0,0062	-2%	-45%	-43%	-33%
**Mean attenuation per model**							-3%	-22%	-26%	-47%

*Effect sizes in replication cohort (LOLIPOP; model 1)

ESM Table 5. List of 76 significant CpG sites associated with incident T2D and overlap with previously published studies on type 2 diabetes, lipids, BMI and blood pressure.

Illumina ID	Gene name	CHR	Position	Incident T2D[a]	Prevalent T2D[b]	Lipids[c]	BMI[d]	Blood Pressure[e]
cg19693031	*TXNIP*	1	145441552	YES	YES	YES		YES
cg06500161	*ABCG1*	21	43656587	YES	YES	YES	YES	YES
cg11024682	*SREBF1*	17	17730094	YES	YES	YES	YES	
cg00574958	*CPT1A*	11	68607622	YES	YES	YES	YES	YES
cg05778424	*AKAP1*	17	55169508	YES			YES	
cg14476101	*PHGDH*	1	120255992	YES			YES	YES
cg04816311	*C7orf50*	7	1066650	YES	YES			
**cg07504977**	***OLMALINC***	**10**	**102131012**				YES	
**cg19750657**	***UFM1***	**13**	**38935967**			YES	YES	
**cg06378491**	***MAP4K2***	**11**	**64564012**					
cg14020176	*SLC9A3R1*	17	72764985	YES			YES	
cg06397161	*SYNGR1*	22	39760059	YES		YES	YES	
**cg06940720**	***LPCAT1***	**5**	**1526929**				YES	
cg02711608	*SLC1A5*	19	47287964	YES	YES		YES	YES
**cg06192883**	***MYO5C***	**15**	**52554171**			YES	YES	
**cg09664445**	***CLUH***	**17**	**2612406**				YES	
**cg14870271**	***LGALS3BP***	**17**	**76976010**				YES	
**cg18568872**	***ZNF710***	**15**	**90606494**				YES	
**cg12257439**	***FER1L5***	**2**	**97360893**					
**cg11269166**	***METTL8***	**2**	**172203847**			YES		
**cg14956201**	***TRIO***	**5**	**14358153**					
**cg17540192**	***TECPR1***	**7**	**97875259**					
**cg27243685**	***ABCG1***	**21**	**43642366**			YES	YES	
**cg11202345**	***LGALS3BP***	**17**	**76976057**				YES	
**cg15020801**	***PNPO***	**17**	**46022809**			YES		

**cg21480264**	***POLN***	**4**	**2137264**					
**cg08788930**	***DENND3***	**8**	**142201685**		YES			
**cg25217710**	***BCAN***	**1**	**156609523**				YES	
**cg22650271**	***SYNGR1***	**22**	**39760165**				YES	
**cg10639435**	***ZNF250***	**8**	**146104221**					
**cg01101459**	***LINC01132***	**1**	**234871477**				YES	
**cg03691549**	***LOC283335***	**12**	**53443911**					
**cg26262157**	***PFKFB3***	**10**	**6214079**		YES			
**cg04927537**	***LGALS3BP***	**17**	**76976091**				YES	
cg08994060	*PFKFB3*	10	6214026	YES	YES			
**cg13059136**	***NAP1L4***	**11**	**2986541**					
**cg21234053**	***CFL2***	**14**	**35163420**					
cg08309687	*LINC00649*	21	35320596	YES			YES	
**cg02879453**	***ADCY7***	**16**	**50321818**					
**cg24259291**	***ZNFX1***	**20**	**47874072**					
**cg26846781**	***KCNH6***	**17**	**61620942**					
**cg16097041**	***FLAD1***	**1**	**154965544**					
**cg01373896**	***KLF16***	**19**	**1854724**					
**cg19169154**	***MFAP4***	**17**	**19287978**					
**cg13300580**	***SLC9A1***	**1**	**27440539**					
**cg23021329**	***TLR9***	**3**	**52256186**					
**cg25001190**	***NFIA***	**1**	**61668835**				YES	
**cg02050917**	***SKI***	**1**	**2173571**				YES	
**cg07719604**	***E2F4***	**16**	**67232460**					
**cg26663590**	***NFATC2IP***	**16**	**28959310**				YES	
**cg17836612**	***LGALS3BP***	**17**	**76976357**				YES	
**cg20507228**	***MAN2A2***	**15**	**91460071**					
**cg04682775**	***SLC6A9***	**1**	**44495089**					
**cg24145109**	***MIR4689***	**1**	**5806951**				YES	

**cg10192877**	***ABCG1***	**21**	**43641690**				YES	
**cg21703988**	***EP400***	**12**	**132549404**					
**cg17901584**	***DHCR24***	**1**	**55353706**			YES	YES	
**cg25178683**	***LGALS3BP***	**17**	**76976267**				YES	
cg25130381	*SLC9A1*	1	27440721	YES				
**cg25649826**	***USP22***	**17**	**20938740**				YES	
**cg20212624**	***CNP***	**17**	**40123227**					
**cg07567724**	***GATAD2B***	**1**	**153777721**			YES		
**cg16861241**	***RNF157-AS1***	**17**	**74138396**					
**cg03819286**	***MGRN1***	**16**	**4673974**					
**cg02079413**	***NAP1L4***	11	**2986505**				YES	
**cg23722778**	***ENPP4***	**6**	**46112967**					
**cg11800635**	***DOK1***	**2**	**74783088**					
**cg25316512**	***ENO2***	**12**	**7032991**					
**cg09294084**	***MCF2L***	**13**	**113646732**					
**cg20784591**	***PILRA***	**7**	**99972461**					
**cg03497652**	***ANKS3***	**16**	**4751569**					
**cg24678869**	***DENND4B***	**1**	**153919638**				YES	
**cg12322877**	***ASPSCR1***	**17**	**79963213**					
**cg09072148**	***NRXN2***	**11**	**64491639**					
**cg14524754**	***B3GNTL1***	**17**	**80925103**					
**cg17194270**	***SYNGR1***	**22**	**39759992**					

[a] Incident T2D: Chambers et al, 2015: Cordona et al, 2019
[b] Prevalent T2D: all studies from review Walaszczyk et al, 2018; Meeks et al, 2019
[c] Lipids: all studies from review Mittelstraß et al, 2018; Tobi et al, 2018
[d] BMI: Wahl et al, 2017; Xu et al, 2018; Demerath et al, 2015; Reed et al, 2020; Wang et al, 2018
[e] Blood pressure: Richard et al, 2017, Huang et al, 2020
Novel CpG sites associated with type 2 diabetes in bold

ESM Table 6. Results of replication and combined discovery and replication cohorts of 76 differentially methylated CpG sites associated with incident type 2 diabetes.

		Discovery results model 1		Replication results model 1		Combined results of discovery and replication cohorts															
**Illumina ID***	**Gene name**	**Effect size discovery**	**P.value discovery**	**Effect size replication**	**P.value replication**	**Effect size model 1**	**P-value model 1**	**I^2 model 1**	**Het. p-value model 1**	**Effect size model 1.1**	**P-value model 1.1**	**I^2 model 1.1**	**Het. p-value model 1.1**	**Effect size model 2**	**P-value model 2**	**I^2 model 2**	**Het. p-value model 2**	**Effect size model 2.1**	**P-value model 2.1**	**I^2 model 2.1**	**Het. p-value model 2.1**
cg19693031	*TXNIP*	-0.020	**4.E-24**	-0.012	**7.E-13**	-0.015	**2.E-32**	39.8	2.E-01	-0.015	**2.E-32**	25.1	2.E-01	-0.014	**5.E-25**	87.5	**5.E-03**	-0.014	**7.E-25**	88.6	**3.E-03**
cg06500161	*ABCG1*	0.011	**7.E-24**	0.009	**7.E-20**	0.010	**4.E-42**	88.9	**3.E-03**	0.010	**2.E-41**	89.5	**2.E-03**	0.007	**1.E-20**	0	8.E-01	0.007	**1.E-20**	0	7.E-01
cg11024682	*SREBF1*	0.009	**5.E-17**	0.008	**6.E-14**	0.008	**8.E-30**	29.9	2.E-01	0.008	**1.E-27**	0	4.E-01	0.006	**7.E-14**	0	1.E+00	0.006	**2.E-12**	0	9.E-01
cg00574958	*CPT1A*	-0.005	**1.E-14**	-0.004	**2.E-07**	-0.005	**2.E-19**	0	6.E-01	-0.004	**9.E-19**	0	5.E-01	-0.003	1.E-07	0	7.E-01	-0.003	2.E-07	0	9.E-01
cg05778424	*AKAP1*	0.008	**4.E-13**	0.004	**1.E-05**	0.006	**6.E-16**	61.8	1.E-01	0.006	**4.E-15**	48.7	2.E-01	0.004	**1.E-07**	0	9.E-01	0.004	3.E-07	0	8.E-01
cg14476101	*PHGDH*	-0.015	**1.E-11**	-0.010	**2.E-06**	-0.013	**2.E-16**	0	1.E+00	-0.012	**9.E-15**	0	8.E-01	-0.009	**8.E-08**	0	5.E-01	-0.008	6.E-07	0	3.E-01
cg04816311	*C7orf50*	0.012	**1.E-11**	0.006	**2.E-03**	0.009	**5.E-13**	0	4.E-01	0.009	**3.E-11**	22.7	3.E-01	0.007	2.E-06	0	6.E-01	0.006	5.E-06	0	8.E-01
**cg07504977**	*OLMALINC*	0.011	**2.E-11**	0.007	**6.E-05**	0.009	**4.E-14**	0	9.E-01	0.008	**3.E-12**	0	7.E-01	0.007	**3.E-08**	0	8.E-01	0.006	3.E-07	50	2.E-01
**cg19750657**	*UFM1*	0.010	**5.E-11**	0.007	**8.E-07**	0.008	**2.E-15**	62.7	1.E-01	0.008	**2.E-14**	80	**3.E-02**	0.005	3.E-07	0	7.E-01	0.005	3.E-06	0	6.E-01
**cg06378491**	*MAP4K2*	0.005	**6.E-11**	0.003	**8.E-05**	0.004	**9.E-14**	85.6	**8.E-03**	0.003	**8.E-11**	52.5	1.E-01	0.003	**4.E-08**	70.8	6.E-02	0.003	3.E-07	0	9.E-01
cg14020176	*SLC9A3R1*	0.009	**7.E-11**	0.003	**4.E-04**	0.005	**1.E-11**	58.5	1.E-01	0.005	**2.E-10**	0	6.E-01	0.004	7.E-06	30.1	2.E-01	0.003	2.E-05	67.2	8.E-02
cg06397161	*SYNGR1*	0.010	**8.E-11**	0.005	**2.E-04**	0.008	**1.E-12**	0	5.E-01	0.008	**1.E-12**	28.3	2.E-01	0.006	5.E-07	73.6	5.E-02	0.006	3.E-07	0	1.E+00
**cg06940720**	*LPCAT1*	0.007	**9.E-11**	0.004	**9.E-05**	0.006	**2.E-13**	8.1	3.E-01	0.005	**9.E-13**	0	5.E-01	0.004	2.E-07	0	9.E-01	0.004	9.E-07	0	6.E-01
cg02711608	*SLC1A5*	-0.009	**1.E-10**	-0.004	**7.E-04**	-0.006	**1.E-10**	0	7.E-01	-0.005	**2.E-09**	0	5.E-01	-0.004	1.E-05	0	7.E-01	-0.004	1.E-04	0	3.E-01
**cg06192883**	*MYO5C*	0.009	**1.E-10**	0.006	**6.E-06**	0.008	**3.E-15**	76.3	**4.E-02**	0.007	**2.E-14**	0	4.E-01	0.005	4.E-07	0	5.E-01	0.005	4.E-07	63.1	1.E-01
**cg09664445**	*CLUH*	0.006	**2.E-10**	0.002	**2.E-02**	0.004	**6.E-10**	51.8	1.E-01	0.004	**4.E-09**	0	6.E-01	0.002	9.E-04	14.6	3.E-01	0.002	1.E-03	43.3	2.E-01
**cg14870271**	*LGALS3BP*	0.008	**2.E-10**	0.006	**4.E-05**	0.007	**5.E-14**	0	4.E-01	0.007	**1.E-12**	76.1	**4.E-02**	0.005	5.E-07	82.3	**2.E-02**	0.005	1.E-06	51.1	2.E-01
**cg18568872**	*ZNF710*	0.006	**2.E-10**	0.003	**5.E-04**	0.004	**2.E-12**	80.7	**2.E-02**	0.004	**5.E-11**	65.4	9.E-02	0.003	3.E-06	81.8	**2.E-02**	0.003	1.E-06	62.1	1.E-01
**cg12257439**	*FER1L5*	0.006	**3.E-10**	0.003	**4.E-05**	0.004	**1.E-12**	0	4.E-01	0.004	**8.E-12**	49.8	2.E-01	0.003	**2.E-08**	19.2	3.E-01	0.003	2.E-07	71.8	6.E-02
**cg11269166**	*METTL8*	0.007	**3.E-10**	0.003	**2.E-02**	0.005	**2.E-09**	77.8	**3.E-02**	0.005	**4.E-09**	77.1	**4.E-02**	0.004	2.E-06	0	6.E-01	0.004	3.E-05	0	4.E-01
**cg14956201**	*TRIO*	0.008	**4.E-10**	0.001	3.E-01	0.005	2.E-07	82.4	**2.E-02**	0.004	8.E-06	37.4	2.E-01	0.004	8.E-05	61.9	1.E-01	0.003	5.E-04	22	3.E-01
**cg17540192**	*TECPR1*	0.005	**4.E-10**	0.003	**8.E-04**	0.004	**4.E-12**	72.7	6.E-02	0.004	**5.E-11**	68.8	7.E-02	0.003	1.E-06	51.6	2.E-01	0.003	1.E-05	0	6.E-01
**cg27243685**	*ABCG1*	0.006	**5.E-10**	0.005	**6.E-13**	0.006	**6.E-21**	74.5	**5.E-02**	0.006	**5.E-21**	72.5	6.E-02	0.004	**1.E-11**	0	4.E-01	0.004	**5.E-12**	53.5	1.E-01

**cg11202345**	*LGALS3BP*	0.008	**8.E-10**	0.006	**5.E-06**	0.007	**6.E-14**	84.9	**1.E-02**	0.007	**8.E-14**	22.4	3.E-01	0.005	**5.E-08**	41.3	2.E-01	0.005	2.E-07	0	4.E-01
**cg15020801**	*PNPO*	0.007	**1.E-09**	0.006	**4.E-07**	0.007	**3.E-15**	49.2	2.E-01	0.007	**2.E-14**	64.4	9.E-02	0.005	**2.E-09**	0	4.E-01	0.005	**2.E-08**	72.1	6.E-02
**cg21480264**	*POLN*	0.006	**1.E-09**	0.003	**2.E-03**	0.004	**6.E-10**	59	1.E-01	0.004	**1.E-09**	0	6.E-01	0.004	**9.E-08**	0	3.E-01	0.003	5.E-06	11.7	3.E-01
**cg08788930**	*DENND3*	0.007	**2.E-09**	0.003	**1.E-03**	0.005	**8.E-11**	0	5.E-01	0.005	**2.E-09**	76.3	**4.E-02**	0.004	3.E-06	61.1	1.E-01	0.004	2.E-05	0	8.E-01
**cg25217710**	*BCAN*	0.005	**2.E-09**	0.003	**4.E-04**	0.004	**6.E-11**	73.6	5.E-02	0.004	**1.E-10**	68.8	7.E-02	0.002	3.E-05	19.9	3.E-01	0.002	2.E-04	0	4.E-01
**cg22650271**	*SYNGR1*	0.006	**3.E-09**	0.003	**9.E-04**	0.004	**3.E-10**	19.5	3.E-01	0.005	**9.E-11**	82.1	**2.E-02**	0.003	8.E-05	18.1	3.E-01	0.003	2.E-05	0	7.E-01
**cg10639435**	*ZNF250*	0.008	**4.E-09**	0.002	3.E-01	0.005	1.E-06	91.5	**6.E-04**	0.005	5.E-06	70.9	6.E-02	0.004	1.E-05	73.7	5.E-02	0.004	2.E-04	76.7	**4.E-02**
**cg01101459**	*LINC01132*	0.007	**4.E-09**	0.009	**1.E-09**	0.008	**5.E-17**	65	9.E-02	0.007	**9.E-16**	64.7	9.E-02	0.006	**6.E-11**	55.6	1.E-01	0.006	**2.E-09**	76.4	**4.E-02**
**cg03691549**	*LOC283335*	0.006	**4.E-09**	0.003	**8.E-03**	0.004	**2.E-09**	66.8	8.E-02	0.004	**2.E-09**	78.3	**3.E-02**	0.003	1.E-05	60.7	1.E-01	0.003	1.E-05	0	4.E-01
**cg26262157**	*PFKFB3*	-0.008	**4.E-09**	-0.005	**1.E-04**	-0.007	**3.E-12**	65.4	9.E-02	-0.007	**5.E-12**	76.1	**4.E-02**	-0.006	**9.E-08**	81.9	**2.E-02**	-0.006	**5.E-08**	68.1	8.E-02
**cg04927537**	*LGALS3BP*	0.011	**5.E-09**	0.006	**4.E-04**	0.009	**2.E-11**	57.8	1.E-01	0.008	**4.E-10**	0.7	3.E-01	0.005	7.E-05	84.4	**1.E-02**	0.005	4.E-04	39.2	2.E-01
cg08994060	*PFKFB3*	-0.010	**5.E-09**	-0.006	**1.E-03**	-0.008	**7.E-11**	60.6	1.E-01	-0.008	**1.E-10**	90.5	**1.E-03**	-0.007	2.E-07	85.8	**8.E-03**	-0.007	1.E-07	67.8	8.E-02
**cg13059136**	*NAP1L4*	0.008	**6.E-09**	0.005	**3.E-04**	0.006	**4.E-11**	82.4	**2.E-02**	0.006	**5.E-10**	44.8	2.E-01	0.005	1.E-05	74.2	**5.E-02**	0.004	7.E-05	93	**2.E-04**
**cg21234053**	*CFL2*	0.015	**7.E-09**	0.005	**2.E-03**	0.008	**9.E-09**	71.1	6.E-02	0.008	**6.E-09**	56.6	1.E-01	0.007	3.E-06	69.4	7.E-02	0.007	1.E-05	82.3	**2.E-02**
cg08309687	*LINC00649*	-0.011	**8.E-09**	-0.007	**4.E-04**	-0.009	**3.E-11**	83.2	**1.E-02**	-0.009	**3.E-10**	10.3	3.E-01	-0.005	2.E-04	75.8	**4.E-02**	-0.005	2.E-04	85.8	**8.E-03**
**cg02879453**	*ADCY7*	0.008	**1.E-08**	0.005	**1.E-05**	0.007	**2.E-12**	36.3	2.E-01	0.007	**3.E-12**	45.2	2.E-01	0.005	7.E-07	93.1	**1.E-04**	0.005	4.E-07	0	5.E-01
**cg24259291**	*ZNFX1*	0.005	**1.E-08**	0.001	5.E-01	0.003	4.E-06	64.7	9.E-02	0.002	3.E-05	29	2.E-01	0.002	1.E-04	79.6	**3.E-02**	0.002	5.E-04	4.3	3.E-01
**cg26846781**	*KCNH6*	0.005	**1.E-08**	0.004	**1.E-06**	0.004	**1.E-13**	37.4	2.E-01	0.004	**2.E-13**	76.8	**4.E-02**	0.003	**1.E-08**	85	**1.E-02**	0.003	**7.E-08**	67.5	8.E-02
**cg16097041**	*FLAD1*	0.006	**1.E-08**	0.003	**1.E-03**	0.004	**1.E-09**	89.5	**2.E-03**	0.004	**8.E-10**	85	**1.E-02**	0.003	7.E-05	86.6	**6.E-03**	0.003	1.E-04	70.5	7.E-02
**cg01373896**	*KLF16*	0.006	**1.E-08**	0.002	8.E-02	0.004	3.E-07	41.7	2.E-01	0.004	**2.E-08**	87.3	**5.E-03**	0.003	2.E-03	0	5.E-01	0.003	4.E-04	19.6	3.E-01
**cg19169154**	*MFAP4*	0.005	**1.E-08**	0.001	5.E-01	0.004	5.E-07	66.4	8.E-02	0.004	5.E-06	80.9	**2.E-02**	0.003	1.E-04	16.7	3.E-01	0.003	6.E-04	80.2	**2.E-02**
**cg13300580**	*SLC9A1*	0.005	**1.E-08**	0.005	**5.E-10**	0.005	**9.E-18**	84	**1.E-02**	0.005	**2.E-16**	82.3	**2.E-02**	0.004	**3.E-11**	38.5	2.E-01	0.004	**4.E-10**	0	4.E-01
**cg23021329**	*TLR9*	0.005	**1.E-08**	0.002	**4.E-02**	0.004	**4.E-08**	90	**2.E-03**	0.003	7.E-07	25.3	2.E-01	0.003	3.E-06	89.2	**2.E-03**	0.003	2.E-04	0	5.E-01
**cg25001190**	*NFIA*	-0.010	**1.E-08**	-0.004	**5.E-03**	-0.007	**9.E-09**	53.5	1.E-01	-0.007	**4.E-08**	86	**8.E-03**	-0.005	3.E-05	15.4	3.E-01	-0.005	8.E-05	80.3	**2.E-02**
**cg02050917**	*SKI*	0.007	**1.E-08**	0.002	7.E-02	0.005	**6.E-08**	77.8	**3.E-02**	0.004	8.E-07	47.1	2.E-01	0.003	3.E-03	77.1	**4.E-02**	0.003	4.E-03	0	3.E-01
**cg07719604**	*E2F4*	0.007	**2.E-08**	0.004	**4.E-04**	0.006	**9.E-11**	82.3	**2.E-02**	0.006	**4.E-09**	85	**1.E-02**	0.004	1.E-05	0	3.E-01	0.004	5.E-05	81.2	**2.E-02**
**cg26663590**	*NFATC2IP*	0.008	**2.E-08**	0.004	**5.E-03**	0.006	**1.E-08**	86.4	**7.E-03**	0.005	**4.E-08**	72.3	6.E-02	0.004	4.E-04	78.2	**3.E-02**	0.003	1.E-03	66.3	9.E-02
**cg17836612**	*LGALS3BP*	0.006	**2.E-08**	0.004	**2.E-04**	0.005	**3.E-11**	61.6	1.E-01	0.005	**5.E-10**	91.2	**7.E-04**	0.004	2.E-06	64.9	9.E-02	0.004	1.E-05	81.4	**2.E-02**
**cg20507228**	*MAN2A2*	0.013	**2.E-08**	0.005	**2.E-03**	0.007	**5.E-09**	75.4	**4.E-02**	0.007	**3.E-08**	67.1	8.E-02	0.006	2.E-05	81.5	**2.E-02**	0.005	5.E-05	0	9.E-01
**cg04682775**	*SLC6A9*	0.007	**2.E-08**	0.001	3.E-01	0.003	2.E-05	82.7	**2.E-02**	0.003	1.E-05	0	3.E-01	0.002	5.E-03	83.6	**1.E-02**	0.002	8.E-03	30	2.E-01

**cg24145109**	*MIR4689*	0.015	**2.E-08**	0.004	1.E-01	0.010	6.E-07	69.8	7.E-02	0.009	5.E-06	59.4	1.E-01	0.007	9.E-04	81.4	**2.E-02**	0.006	4.E-03	70.2	7.E-02
**cg10192877**	*ABCG1*	0.004	**3.E-08**	0.002	**3.E-03**	0.003	**3.E-09**	88	**4.E-03**	0.003	**6.E-09**	90.3	**1.E-03**	0.002	2.E-04	35	2.E-01	0.002	4.E-04	57	1.E-01
**cg21703988**	*EP400*	0.005	**3.E-08**	0.002	**1.E-02**	0.003	**2.E-08**	79.3	**3.E-02**	0.003	2.E-07	86.8	**6.E-03**	0.003	5.E-06	0	4.E-01	0.003	8.E-05	87.8	**4.E-03**
**cg17901584**	*DHCR24*	-0.009	**3.E-08**	-0.010	**6.E-10**	-0.010	**1.E-16**	90	**2.E-03**	-0.009	**9.E-16**	78.5	**3.E-02**	-0.007	**9.E-09**	2.7	3.E-01	-0.007	**5.E-08**	92.8	**2.E-04**
**cg25178683**	*LGALS3BP*	0.008	**3.E-08**	0.006	**3.E-05**	0.007	**5.E-12**	82.5	**2.E-02**	0.007	**1.E-11**	30.9	2.E-01	0.005	7.E-06	92	**4.E-04**	0.005	2.E-05	65.8	9.E-02
cg25130381	*SLC9A1*	0.006	**4.E-08**	0.005	**2.E-07**	0.005	**3.E-14**	83.5	**1.E-02**	0.005	**5.E-14**	79.8	**3.E-02**	0.004	**4.E-08**	0	8.E-01	0.004	2.E-07	73.6	5.E-02
**cg25649826**	*USP22*	0.006	**4.E-08**	0.002	**4.E-02**	0.003	1.E-06	83	**2.E-02**	0.003	1.E-06	73.6	5.E-02	0.002	1.E-02	65.9	9.E-02	0.002	2.E-02	0	5.E-01
**cg20212624**	*CNP*	0.007	**5.E-08**	0.003	**1.E-03**	0.004	**3.E-09**	73	5.E-02	0.004	1.E-07	81.5	**2.E-02**	0.004	4.E-06	77.1	**4.E-02**	0.003	3.E-05	85.5	**9.E-03**
**cg07567724**	*GATAD2B*	0.008	**5.E-08**	0.001	4.E-01	0.004	1.E-05	84.4	**1.E-02**	0.004	3.E-05	80.4	**2.E-02**	0.003	9.E-04	85.8	**8.E-03**	0.003	1.E-03	40.3	2.E-01
**cg16861241**	*RNF157-AS1*	0.005	**5.E-08**	0.000	7.E-01	0.002	2.E-04	85.9	**8.E-03**	0.003	6.E-05	63	1.E-01	0.001	5.E-02	92.6	**2.E-04**	0.002	1.E-02	0	5.E-01
**cg03819286**	*MGRN1*	0.006	**5.E-08**	0.003	**2.E-03**	0.004	**2.E-09**	92.6	**2.E-04**	0.004	**2.E-08**	81.2	**2.E-02**	0.003	1.E-04	45.1	2.E-01	0.003	5.E-04	57.7	1.E-01
**cg02079413**	*NAP1L4*	0.007	**5.E-08**	0.006	**3.E-06**	0.007	**3.E-12**	88.2	**4.E-03**	0.006	**2.E-11**	80.1	**2.E-02**	0.005	2.E-07	0	4.E-01	0.005	2.E-06	90.7	**1.E-03**
**cg23722778**	*ENPP4*	-0.009	**7.E-08**	-0.001	4.E-01	-0.003	3.E-04	83.1	**1.E-02**	-0.003	4.E-04	85.3	**9.E-03**	-0.003	5.E-04	63.6	1.E-01	-0.003	8.E-04	82.4	**2.E-02**
**cg11800635**	*DOK1*	0.009	**7.E-08**	0.005	**3.E-05**	0.007	**2.E-11**	79.5	**3.E-02**	0.007	**2.E-11**	90.3	**1.E-03**	0.005	8.E-06	84.6	**1.E-02**	0.005	2.E-06	48.6	2.E-01
**cg25316512**	*ENO2*	0.005	**7.E-08**	0.002	**7.E-03**	0.003	**5.E-09**	88.7	**3.E-03**	0.003	8.E-07	86.3	**7.E-03**	0.003	1.E-05	93.5	**9.E-05**	0.002	1.E-04	93.3	**1.E-04**
**cg09294084**	*MCF2L*	0.011	**8.E-08**	0.006	**2.E-03**	0.008	**2.E-09**	87.5	**5.E-03**	0.008	**4.E-08**	72.1	6.E-02	0.007	1.E-06	86.3	**7.E-03**	0.006	3.E-05	83.9	**1.E-02**
**cg20784591**	*PILRA*	0.004	**8.E-08**	0.003	**3.E-04**	0.003	**9.E-10**	90.7	**1.E-03**	0.003	**1.E-08**	91.1	**8.E-04**	0.003	3.E-07	89	**3.E-03**	0.003	3.E-06	81.3	**2.E-02**
**cg03497652**	*ANKS3*	0.009	**8.E-08**	0.006	**3.E-04**	0.007	**3.E-10**	91.9	**4.E-04**	0.007	**3.E-09**	91.8	**5.E-04**	0.006	8.E-07	87.4	**5.E-03**	0.005	8.E-06	87.1	**5.E-03**
**cg24678869**	*DENND4B*	0.004	**9.E-08**	0.003	**5.E-05**	0.003	**8.E-11**	91.2	**8.E-04**	0.003	**1.E-10**	90.1	**1.E-03**	0.002	1.E-05	83.2	**1.E-02**	0.002	5.E-05	81.6	**2.E-02**
**cg12322877**	*ASPSCR1*	0.012	**9.E-08**	0.006	**6.E-03**	0.008	**2.E-08**	91.7	**5.E-04**	0.009	**4.E-09**	90.2	**1.E-03**	0.006	3.E-04	85	**1.E-02**	0.006	8.E-05	82.1	**2.E-02**
**cg09072148**	*NRXN2*	0.004	**9.E-08**	0.000	4.E-01	0.002	8.E-05	91.4	**7.E-04**	0.002	6.E-05	93.5	**9.E-05**	0.002	1.E-04	88.5	**3.E-03**	0.002	2.E-04	88.1	**4.E-03**
**cg14524754**	*B3GNTL1*	0.007	**9.E-08**	0.003	**4.E-02**	0.005	3.E-07	92.6	**2.E-04**	0.004	1.E-06	92	**4.E-04**	0.005	2.E-06	84.2	**1.E-02**	0.004	1.E-05	90.2	**1.E-03**
**cg17194270**	*SYNGR1*	0.009	**1.E-07**	0.006	**2.E-04**	0.008	**1.E-10**	93.8	**6.E-05**	0.008	**4.E-10**	93.4	**1.E-04**	0.005	1.E-04	87.1	**5.E-03**	0.005	9.E-05	78.7	**3.E-02**

*novel findings in bold ; P-values <0.05 OR in combined effects <0.00065 in bold

ESM Table 7. KEGG enriched pathways based on meta-analysis results from incident T2D EWAS.

**Description**	**Size**	**Pvalue model 1**	**Padj model 1**	**Pvalue model 2.1**	**Padj model 2.1**
Insulin signaling pathway	138	0.003	0.125	0.293	0.752
Chagas disease (American trypanosomiasis)	104	0.001	0.195	-	-
Toll-like receptor signaling pathway	102	0.002	0.218	0.238	0.752
Natural killer cell mediated cytotoxicity	136	0.020	0.218	0.003	0.124
Chemokine signaling pathway	189	0.033	0.240	0.298	0.752
Cytokine-cytokine receptor interaction	265	0.033	0.240	0.477	0.861
Osteoclast differentiation	128	0.038	0.240	0.131	0.638

ESM Table 8. Reactome enriched pathways based on meta-analysis results from incident T2D EWAS.

ID	Description	Size	Pvalue model 1	Padj model 1	Pvalue model 2.1	Padj model 2.1
R-HSA-1483257	Homo sapiens: Phospholipid metabolism	194	0.0003	0.046	0.004	0.172
R-HSA-8957322	Homo sapiens: Metabolism of steroids	140	0.004	0.233	0.157	0.805
R-HSA-194840	Homo sapiens: Rho GTPase cycle	112	0.004	0.233	0.002	0.164
R-HSA-202733	Homo sapiens: Cell surface interactions at the vascular wall	130	0.007	0.280	0.137	0.767
R-HSA-9007101	Homo sapiens: Rab regulation of trafficking	104	0.011	0.303	0.050	0.591
R-HSA-168898	Homo sapiens: Toll-like Receptor Cascades	141	0.011	0.303	0.124	0.755
R-HSA-425407	Homo sapiens: SLC-mediated transmembrane transport	234	0.020	0.488	1	1
R-HSA-1630316	Homo sapiens: Glycosaminoglycan metabolism	114	0.028	0.516	0.012	0.254
R-HSA-1483206	Homo sapiens: Glycerophospholipid biosynthesis	117	0.030	0.516	0.116	0.755
R-HSA-198933	Homo sapiens: Immunoregulatory interactions between a Lymphoid and a non-Lymphoid cell	119	0.034	0.516	0.251	0.805
R-HSA-9006925	Homo sapiens: Intracellular signaling by second messengers	274	0.036	0.516	0.003	0.164
R-HSA-76002	Homo sapiens: Platelet activation, signaling and aggregation	247	0.037	0.516	0.081	0.753
R-HSA-425393	Homo sapiens: Transport of inorganic cations/anions and amino acids/oligopeptides	102	0.045	0.578	0.774	1.000

ESM Table 9. Enriched GO terms based on meta-analysis results from incident T2D EWAS.

ID	Description	Size	Pvalue model 1	Padj model 1	Pvalue model 2.1	Padj model 2.1
GO:0045834	positive regulation of lipid metabolic process	156	5.12E-10	1.00E-06	9,71E-05	0,119
GO:0071216	cellular response to biotic stimulus	264	1.87E-07	1.40E-04	2,68E-04	0,119
GO:0019216	regulation of lipid metabolic process	469	2.15E-07	1.40E-04	0,017	0,492
GO:0062013	positive regulation of small molecule metabolic process	163	4.86E-07	2.00E-04	0,006	0,361
GO:0016126	sterol biosynthetic process	111	5.26E-07	2.00E-04	-	-
GO:1902653	secondary alcohol biosynthetic process	100	6.12E-07	2.00E-04	-	-
GO:0008203	cholesterol metabolic process	195	8.80E-07	2.32E-04	0,030	0,574
GO:1902652	secondary alcohol metabolic process	201	9.51E-07	2.32E-04	0,031	0,574
GO:0019218	regulation of steroid metabolic process	131	8.81E-06	0.002	0,042	0,631
GO:0016125	sterol metabolic process	229	9.76E-06	0.002	0,075	0,807
GO:0006066	alcohol metabolic process	493	1.11E-05	0.002	0,082	0,839
GO:0062012	regulation of small molecule metabolic process	438	1.35E-05	0.002	-	-
GO:0046890	regulation of lipid biosynthetic process	210	2.02E-05	0.003	0,010	0,446
GO:0001818	negative regulation of cytokine production	298	4.03E-05	0.006	0,049	0,663
GO:0002286	T cell activation involved in immune response	110	4.96E-05	0.006	-	-
GO:1903825	organic acid transmembrane transport	169	8.40E-05	0.009	0,091	0,863
GO:1905039	carboxylic acid transmembrane transport	169	8.40E-05	0.009	0,091	0,863
GO:0009897	external side of plasma membrane	383	8.50E-05	0.009	0,248	1,000
GO:0071219	cellular response to molecule of bacterial origin	238	1.02E-04	0.011	0,040	0,618
GO:0043506	regulation of JUN kinase activity	113	1.37E-04	0.013	-	-
GO:0030258	lipid modification	382	1.53E-04	0.014	0,367	1,000
GO:0019217	regulation of fatty acid metabolic process	103	1.74E-04	0.015	0,322	1,000
GO:0000187	activation of MAPK activity	173	1.98E-04	0.017	0,001	0,227
GO:0071222	cellular response to lipopolysaccharide	219	2.17E-04	0.018	0,046	0,652
GO:0008202	steroid metabolic process	476	2.44E-04	0.019	0,075	0,807

GO:0051235	maintenance of location	409	2.47E-04	0.019	0,003	0,227
GO:0000302	response to reactive oxygen species	247	2.86E-04	0.021	0,013	0,469
GO:0042542	response to hydrogen peroxide	151	3.02E-04	0.021	0,010	0,446
GO:0046165	alcohol biosynthetic process	199	3.06E-04	0.021	0,271	1,000
GO:0010596	negative regulation of endothelial cell migration	111	3.22E-04	0.021	0,438	1,000
GO:0043433	negative regulation of DNA-binding transcription factor activity	180	4.23E-04	0.027	0,183	1,000
GO:0002285	lymphocyte activation involved in immune response	194	4.60E-04	0.028	0,032	0,574
GO:0051250	negative regulation of lymphocyte activation	160	0.001	0.034	0,023	0,501
GO:0002237	response to molecule of bacterial origin	386	0.001	0.036	0,040	0,618
GO:0060090	molecular adaptor activity	213	0.001	0.038	0,001	0,145
GO:0006694	steroid biosynthetic process	274	0.001	0.038	0,320	1,000
GO:0042632	cholesterol homeostasis	116	0.001	0.038	0,125	0,959
GO:0055092	sterol homeostasis	117	0.001	0.038	0,125	0,959
GO:0048017	inositol lipid-mediated signaling	192	0.001	0.038	0,202	1,000
GO:0014065	phosphatidylinositol 3-kinase signaling	154	0.001	0.038	0,233	1,000
GO:0048015	phosphatidylinositol-mediated signaling	189	0.001	0.039	0,218	1,000
GO:1903510	mucopolysaccharide metabolic process	149	0.001	0.041	0,021	0,492
GO:1901617	organic hydroxy compound biosynthetic process	340	0.001	0.044	0,540	1,000
GO:0010565	regulation of cellular ketone metabolic process	151	0.001	0.044	0,114	0,929
GO:0002040	sprouting angiogenesis	225	0.001	0.044	0,057	0,700
GO:0042035	regulation of cytokine biosynthetic process	113	0.001	0.044	0,213	1,000
GO:0008286	insulin receptor signaling pathway	161	0.001	0.045	0,003	0,227
GO:0070555	response to interleukin-1	175	0.001	0.046	0,337	1,000
GO:0010633	negative regulation of epithelial cell migration	129	0.001	0.048	0,233	1,000
GO:0071902	positive regulation of protein serine/threonine kinase activity	402	0.001	0.048	0,002	0,227
GO:0042626	ATPase activity, coupled to transmembrane movement of substances	188	0.001	0.048	-	-
GO:0008194	UDP-glycosyltransferase activity	286	0.001	0.050	3,52E-04	0,119

ESM Table 10. Transcription factors associated with genes annotated from incident T2D meta-EWAS.

Rank	TF	Score	Library	Overlapping_Genes
1	FLYWCH1	6.14E-04	ARCHS4 Coexpression,6.143E-4	SKI,SYNGR1,DENND4B,MGRN1,MCF2L,NRXN2,KLF16,TECPR1
2	MXD4	6.22E-04	GTEx Coexpression,6.223E-4	SKI,SYNGR1,MCF2L,ANKS3,NRXN2,ENO2
3	SREBF1	7.12E-04	Enrichr Queries,7.123E-4	LGALS3BP,SLC9A3R1,CPT1A,PFKFB3,NFIA,TXNIP,PHGDH,DHCR24,SLC1A5,ABCG1
4	MEF2D	0.001229	ARCHS4 Coexpression,0.001229	SKI,CLUH,MAN2A2,DENND4B,MGRN1,DENND3,ZNF710,KLF16
5	ZNF286B	0.001245	GTEx Coexpression,0.001245	SKI,SYNGR1,TRIO,MCF2L,NRXN2,ENO2
6	ZNF692	0.001425	Enrichr Queries,0.001425	LGALS3BP,SREBF1,PFKFB3,DENND4B,NFIA,TXNIP,PHGDH,SLC1A5
7	MECP2	0.001843	ARCHS4 Coexpression,0.001843	SKI,MAN2A2,DENND4B,MGRN1,MCF2L,NRXN2,GATAD2B
8	ZNF732	0.001867	GTEx Coexpression,0.001867	SKI,SYNGR1,MCF2L,ANKS3,NRXN2,ENO2
9	SNAPC4	0.002137	Enrichr Queries,0.002137	TRIO,CLUH,PFKFB3,EP400,TXNIP,PHGDH,SLC1A5
10	CIC	0.002457	ARCHS4 Coexpression,0.002457	SKI,SREBF1,CLUH,DENND4B,MGRN1,KLF16
11	NFIX	0.002489	GTEx Coexpression,0.002489	SKI,SYNGR1,MCF2L,USP22,NRXN2,ENO2
12	ZBTB40	0.002849	Enrichr Queries,0.002849	TRIO,PFKFB3,NFIA,TXNIP,PHGDH,SLC1A5,SLC9A1
13	SOX10	0.003071	ARCHS4 Coexpression,0.003071	LGALS3BP,BCAN,SYNGR1,CNP,MAN2A2,MCF2L
14	ZBTB46	0.003111	GTEx Coexpression,0.003111	SKI,SYNGR1,MCF2L,NRXN2,ENO2
15	RUNX1	0.003367	ReMap ChIP-seq,0.003367	SREBF1,CPT1A,PFKFB3,DENND4B,DENND3,SLC1A5,ENO2,KLF16,SKI,DOK1,SLC9A3R1,MFAP4,MAN2A2,FLAD1,TLR9,E2F4,ABCG1
16	AHDC1	0.003561	Enrichr Queries,0.003561	SREBF1,TRIO,NFIA,EP400,TXNIP,NRXN2,SLC9A1
17	KLF13	0.003686	ARCHS4 Coexpression,0.003686	SKI,DENND4B,TXNIP,DENND3,ZNF710,ADCY7
18	ZNF528	0.003734	GTEx Coexpression,0.003734	SKI,MCF2L,ANKS3,NRXN2,ENO2
19	DDIT3	0.004274	Enrichr Queries,0.004274	LGALS3BP,PFKFB3,SLC6A9,TXNIP,PHGDH,SLC1A5,ENO2
20	MLXIP	0.0043	ARCHS4 Coexpression,0.0043	SKI,CPT1A,CLUH,MAN2A2,DENND4B,MGRN1
21	SKI	0.004356	GTEx Coexpression,0.004356	SYNGR1,NFIA,MCF2L,NRXN2,ENO2
22	ZNF319	0.004914	ARCHS4 Coexpression,0.004914	DENND4B,TXNIP,DENND3,ZNF710,PILRA,KLF16
23	ZIC2	0.004978	GTEx Coexpression,0.004978	SKI,SYNGR1,MCF2L,NRXN2,ENO2
24	ZNF496	0.004986	Enrichr Queries,0.004986	SREBF1,TRIO,PFKFB3,SLC6A9,TXNIP,PHGDH,SLC1A5

25	PRR12	0.005528	ARCHS4 Coexpression,0.005528	SKI,SREBF1,CLUH,DENND4B,MGRN1,KLF16
26	LHX5	0.0056	GTEx Coexpression,0.0056	SYNGR1,MCF2L,USP22,NRXN2,ENO2
27	ZNF213	0.005698	Enrichr Queries,0.005698	SREBF1,PFKFB3,NFIA,MGRN1,SLC1A5,KLF16,SLC9A1
28	TAL1	0.006098	Literature ChIP-seq,0.006098	LGALS3BP,CPT1A,TRIO,LPCAT1,ADCY7,KLF16,SLC9A1,SYNGR1,ZNFX1,NFIA,SLC6A9,MYO5C,PNPO,TXNIP,E2F4,ABCG1
29	ZNF865	0.006143	ARCHS4 Coexpression,0.006143	SKI,SREBF1,CLUH,DENND4B,MGRN1,KLF16
30	BARHL2	0.006223	GTEx Coexpression,0.006223	SKI,SYNGR1,MCF2L,NRXN2,ENO2
31	PRDM14	0.006734	ReMap ChIP-seq,0.006734	LGALS3BP,SREBF1,CPT1A,DENND4B,ASPSCR1,NRXN2,RNF157-AS1,ENO2,SKI,BCAN,MAN2A2,MIR4689,SLC6A9,FLAD1,MCF2L
32	FOXJ2	0.006757	ARCHS4 Coexpression,0.006757	SKI,MAN2A2,DENND4B,TXNIP,DENND3,ADCY7
33	UNCX	0.006845	GTEx Coexpression,0.006845	SKI,SYNGR1,MCF2L,NRXN2,ENO2
34	TFAP4	0.007123	Enrichr Queries,0.007123	SREBF1,PFKFB3,NFIA,TXNIP,PHGDH,SLC1A5,KLF16
35	PPARD	0.007371	ARCHS4 Coexpression,0.007371	SKI,PFKFB3,DENND4B,MGRN1,KLF16,SLC9A1
36	NEUROD1	0.007467	GTEx Coexpression,0.007467	SKI,SYNGR1,MCF2L,NRXN2,ENO2
37	ZFPM1	0.007835	Enrichr Queries,0.007835	LGALS3BP,SREBF1,SLC9A3R1,CPT1A,TRIO,PFKFB3,NFIA
38	STAT5B	0.007985	ARCHS4 Coexpression,0.007985	MAN2A2,DENND4B,TXNIP,DENND3,PILRA,ADCY7
39	POU2F1	0.00809	GTEx Coexpression,0.00809	SKI,MCF2L,NRXN2,ENO2,GATAD2B
40	TFAP2A	0.008475	ENCODE ChIP-seq,0.008475	CPT1A,PFKFB3,DENND3,SLC1A5,KLF16,GATAD2B,AKAP1,NAP1L4,OLMALINC,BCAN,DOK1,ZNFX1,MAN2A2,NFIA,SLC6A9,FLAD1,METTL8
41	ZBTB7B	0.008547	Enrichr Queries,0.008547	LGALS3BP,SREBF1,CPT1A,PFKFB3,NFIA,TXNIP,SLC1A5
42	GFI1	0.0086	ARCHS4 Coexpression,0.0086	CPT1A,MAN2A2,DENND4B,DENND3,ADCY7,KLF16
43	ZNF248	0.008712	GTEx Coexpression,0.008712	SYNGR1,MCF2L,ANKS3,NRXN2,ENO2
44	ZBTB42	0.009214	ARCHS4 Coexpression,0.009214	SREBF1,SLC9A3R1,CPT1A,CLUH,MYO5C,SLC1A5
45	ZNF781	0.009334	GTEx Coexpression,0.009334	SKI,SYNGR1,MCF2L,NRXN2,ENO2
46	DOT1L	0.009828	ARCHS4 Coexpression,0.009828	SKI,CLUH,PFKFB3,DENND4B,MGRN1,KLF16
47	ZNF521	0.009956	GTEx Coexpression,0.009956	SKI,SYNGR1,MCF2L,NRXN2,ENO2
48	ZBTB48	0.009972	Enrichr Queries,0.009972	SREBF1,SLC9A3R1,PFKFB3,ASPSCR1,TXNIP,SLC1A5,SLC9A1

ESM Table 11. GWAS-catalog reported traits associated with genes from annotated CpG sites from meta-analysis on incident type 2 diabetes.

	distance	nearestGeneSymbol	GWAS-catalog reported trait(s) at 5e-8
cg19693031	0	*TXNIP*	/
cg06500161	0	*ABCG1*	Birth weight
cg11024682	0	*SREBF1*	Resting heart rate;Coffee consumption;
cg00574958	0	*CPT1A*	Serum metabolite levels, Serum metabolite levels (CMS); Total body bone mineral density; **Lipid metabolism phenotypes;** Heel bone mineral density; **Lipid traits (pleiotropy) (HIPO component 1);** Urate levels; Appendicular lean mass;
cg05778424	0	*AKAP1*	Male-pattern baldness, Balding type 1; Male-pattern baldness; Freckles; Hair color; Blond vs. brown/black hair color;
cg14476101	0	*PHGDH*	Multiple sclerosis; Glycine levels; Height; Red blood cell count; **Metabolic traits;** Metabolite levels; Blood metabolite levels; Metabolite levels; Breast cancer; **Total cholesterol levels;** Macular telangiectasia type 2; Core binding factor acute myeloid leukemia; Amino acid levels;
cg04816311	0	*C7orf50*	**Cholesterol, total, Total cholesterol levels;** Mean corpuscular hemoglobin; Mean arterial pressure; C-reactive protein levels or total cholesterol levels (pleiotropy); **LDL cholesterol;**
cg07504977	2319	*OLMALINC*	/
cg19750657	0	*UFM1*	/
cg06378491	0	*MAP4K2*	Urate levels
cg14020176	0	*SLC9A3R1*	Monocyte percentage of white cells; Monocyte count;
cg06397161	0	*SYNGR1*	Rheumatoid arthritis, Rheumatoid arthritis (ACPA-positive); Primary biliary cholangitis, Primary biliary cirrhosis; IgG glycosylation; Factor VIII levels; vWF levels; Intelligence; Cholesteryl ester levels; Blond vs. brown/black hair color; Inflammatory bowel disease;
cg06940720	2852	*LPCAT1*	/
cg02711608	0	*SLC1A5*	Height
cg06192883	0	*MYO5C*	/
cg09664445	0	*CLUH*	Height; Red cell distribution width;
cg14870271	0	*LGALS3BP*	Height;
cg18568872	0	*ZNF710*	Height;

cg12257439	0	*FER1L5*	Diastolic blood pressure x smoking status (ever vs never) interaction (2df test), Diastolic blood pressure x smoking status (current vs non-current) interaction (2df test); Diastolic blood pressure x alcohol consumption interaction (2df test); Eosinophil counts;
cg11269166	0	*METTL8*	Appendicular lean mass; Height;
cg14956201	0	*TRIO*	DNA methylation variation (age effect); Lung function (FEV1/FVC); Medication use (adrenergics, inhalants);
cg17540192	0	*TECPR1*	Urate levels; Red blood cell count;
cg27243685	0	*ABCG1*	Birth weight;
cg11202345	0	*LGALS3BP*	Height;
cg15020801	0	*PNPO*	/
cg21480264	0	*POLN*	**Diastolic blood pressure;** Height;
cg08788930	0	*DENND3*	Isovolumetric relaxation time; Response to interferon beta therapy; White blood cell count ；
cg25217710	2215	*BCAN*	Blood protein levels ；
cg22650271	0	*SYNGR1*	Rheumatoid arthritis, Rheumatoid arthritis (ACPA-positive); Primary biliary cholangitis, Primary biliary cirrhosis; IgG glycosylation; Factor VIII levels; vWF levels; Intelligence; **Cholesteryl ester levels;** Blond vs. brown/black hair color; Inflammatory bowel disease;
cg10639435	0	*ZNF250*	Core binding factor acute myeloid leukemia;
cg01101459	4086	*LINC01132*	**LDL cholesterol, LDL cholesterol levels, Low density lipoprotein cholesterol levels; Cholesterol, total, Total cholesterol levels**； White blood cell count (eosinophil); Heel bone mineral density； Medication use (HMG CoA reductase inhibitors)
cg03691549	0	*LOC283335*	/
cg26262157	0	*PFKFB3*	Soluble interleukin-2 receptor subunit alpha; **Latent autoimmune diabetes;** Blood protein levels;
cg04927537	29	*LGALS3BP*	Height;
cg08994060	0	*PFKFB3*	Soluble interleukin-2 receptor subunit alpha; **Latent autoimmune diabetes;** Blood protein levels;
cg13059136	0	*NAP1L4*	**Type 2 diabetes; High density lipoprotein cholesterol levels;** White blood cell count; Eosinophil counts;

cg21234053	16166	*CFL2*	Atrial fibrillation;
cg08309687	0	*LINC00649*	Peak expiratory flow; Estimated glomerular filtration rate; Blood urea nitrogen levels; Lung function (FEV1/FVC); Menarche (age at onset); Urate levels; Hemoglobin concentration; Hematocrit, Red blood cell traits; Red blood cell count;
cg02879453	0	*ADCY7*	Ulcerative colitis; Pediatric autoimmune diseases; Hypothyroidism; Inflammatory bowel disease; Autoimmune traits; Birth weight; Medication use (thyroid preparations);
cg24259291	0	*ZNFX1*	General cognitive ability;<br>Infant length;
cg26846781	0	*KCNH6*	/
cg16097041	0	*FLAD1*	/
cg01373896	0	*KLF16*	**Body mass index**； Mean corpuscular hemoglobin ； Educational attainment (years of education), Educational attainment (MTAG) ； Mean corpuscular volume ； Red blood cell count ； Lung function (FEV1/FVC) ； Cognitive performance (MTAG) ； Highest math class taken (MTAG) ；
cg19169154	0	*MFAP4*	/
cg13300580	0	*SLC9A1*	/
cg23021329	0	*TLR9*	Worry
cg25001190	0	*NFIA*	Electrocardiographic conduction measures, QRS duration; Heel bone mineral density;<br>Celiac disease; Thyroid hormone levels; General risk tolerance (MTAG), Risk-taking tendency (4-domain principal component model); **High density lipoprotein cholesterol levels;** Educational attainment (MTAG), Educational attainment (years of education); Self-reported math ability (MTAG), Highest math class taken (MTAG); QRS duration; Global electrical heterogeneity phenotypes; Height; Number of sexual partners; Blond vs. brown/black hair color; Worry; Myopia (age of diagnosis); Myopia; Feeling nervous; Red blood cell count;Cognitive performance (MTAG); Hair color; Regular attendance at a religious group; Spherical equivalent or myopia (age of diagnosis); QRS complex (Cornell); DNA methylation variation (age effect);
cg02050917	0	*SKI*	Coronary artery disease ； Height ； Platelet distribution width ； Educational attainment (years of education) ； Red cell distribution width ； Lung function (FVC) ； **Systolic blood pressure ；** Sedentary behaviour duration ； PR interval ； Urate levels ；
cg07719604	0	*E2F4*	**HDL cholesterol;** Mean corpuscular hemoglobin;

cg26663590	2816	*NFATC2IP*	General cognitive ability, Intelligence (MTAG)；Cognitive performance (MTAG)；**Body mass index；** Non-albumin protein levels；
cg17836612	295	*LGALS3BP*	Height;
cg20507228	0	*MAN2A2*	/
cg04682775	0	*SLC6A9*	Educational attainment (MTAG), Educational attainment (years of education)；Attention deficit hyperactivity disorder or cannabis use；Mean corpuscular volume；
cg24145109	115779	*MIR4689*	Adolescent idiopathic scoliosis; Prostate cancer;
cg10192877	0	*ABCG1*	Birth weight;
cg21703988	0	*EP400*	Red cell distribution width；Mean corpuscular hemoglobin；Red blood cell count；
cg17901584	784	*DHCR24*	Height
cg25178683	205	*LGALS3BP*	Height
cg25130381	0	*SLC9A1*	/
cg25649826	0	*USP22*	/
cg20212624	0	*CNP*	Blood protein levels
cg07567724	0	*GATAD2B*	Self-reported math ability, Self-reported math ability (MTAG), Highest math class taken (MTAG), Highest math class taken；Intelligence, General cognitive ability; Male-pattern baldness, Balding type 1; Cognitive performance (MTAG), Cognitive performance; Mean corpuscular hemoglobin; Height; C-reactive protein levels; Educational attainment (MTAG);
cg16861241	0	*RNF157-AS1*	/
cg03819286	849	*MGRN1*	/
cg02079413	0	*NAP1L4*	**Type 2 diabetes; High density lipoprotein cholesterol levels;** White blood cell count; Eosinophil counts;
cg23722778	0	*ENPP4*	Blood protein levels;
cg11800635	0	*DOK1*	Height
cg25316512	131	*ENO2*	/
cg09294084	0	*MCF2L*	Factor VII, Factor VII activity, Factor VII activity or levels; **Systolic blood pressure;** End-stage coagulation; Coronary artery disease; Pulse pressure; Prothrombin time; Osteoarthritis; **Cardiovascular disease;**

cg20784591	0	*PILRA*	Alzheimer's disease or family history of Alzheimer's disease ; Heel bone mineral density ; Family history of Alzheimer's disease ; Alzheimer's disease (late onset) ; Hair color ; Advanced age-related macular degeneration ;
cg03497652	0	*ANKS3*	**High density lipoprotein cholesterol levels;** Height;
cg24678869	483	*DENND4B*	/
cg12322877	0	*ASPSCR1*	Lung function (FVC) ; FEV1 ; Birth weight ; Offspring birth weight ; Red blood cell count ; Lung function (FEV1/FVC) ; Alcohol consumption (drinks per week) (MTAG) ; **Waist-hip ratio ;** Smoking initiation (ever regular vs never regular) (MTAG) ;
cg09072148	978	*NRXN2*	**Body mass index;** Gout, Renal underexcretion gout, Renal overload gout; Serum uric acid levels, Uric acid levels; Urate levels; Renal function-related traits (urea); Smoking initiation (ever regular vs never regular); Highest math class taken;
cg14524754	0	*B3GNTL1*	Eosinophil counts ;
cg17194270	0	*SYNGR1*	Rheumatoid arthritis, Rheumatoid arthritis (ACPA-positive); Primary biliary cholangitis, Primary biliary cirrhosis; IgG glycosylation; Factor VIII levels; vWF levels; Intelligence; **Cholesteryl ester levels;** Blond vs. brown/black hair color; Inflammatory bowel disease;

ESM Table 12. EWAS-catalog reported traits associated with CpG sites from meta-analysis results on incident type 2 diabetes.

	distance	Nearest Gene Symbol	EWAS-catalog reported trait(s)
cg19693031	0	*TXNIP*	Alcohol consumption; Alcohol consumption per day; Autoantibody production in systemic lupus erythematosus; **Concentration of small HDL particles; Diastolic Blood Pressure**; **fasting glucose**; Fetal vs adult liver; **Free cholesterol in small HDL**; **Glucose**; **HbA1c**; Hepatic Fat; **Hypertriglyceridemic waist**; Leucine; M35186_1-arachidonoylglycerophosphoethanolamine*; Maternal smoking in pregnancy; Mediterranean diet; Nonalcoholic fatty liver disease; **Phospholipids in small HDL**; Prenatal smoke exposure; Rheumatoid arthritis; **Serum triglycerides**; Sex; **Systolic Blood Pressure**; Tissue; **Total lipids in small HDL**; **Triglycerides**; **Type II diabetes**; **Type II diabetes mellitus**
cg06500161	0	*ABCG1*	**2-hour glucose; 2-hour insulin;** Age 4 vs age 0; Alanine; Android fat free mass; Android fat mass; Android lean mass; Android region fat; Android tissue fat; Android tissue mass; Android total mass; Apolipoprotein B; Arm circumference; Arm fat mass; Arm region fat; Arm tissue fat; Arm total mass; Arms tissue mass; **BMI**; **Body mass index**; **Cholesterol esters in chylomicrons and extremely large VLDL; Cholesterol esters in large HDL; Cholesterol esters in large VLDL; Cholesterol esters in medium VLDL; Cholesterol esters in small VLDL; Cholesterol esters in very large HDL; Cholesterol esters in very large VLDL; Cholesterol esters to total lipids ratio in large HDL; Cholesterol esters to total lipids ratio in very small VLDL; Concentration of chylomicrons and extremely large VLDL particles; Concentration of large HDL particles; Concentration of large VLDL particles; Concentration of medium VLDL particles; Concentration of small VLDL particles; Concentration of very large HDL particles; Concentration of very large VLDL particles; Concentration of very small VLDL particles; C-Reactive Protein; Diastolic Blood Pressure**; **Fasting glucose**; **fasting insulin**; **Free cholesterol in chylomicrons and extremely large VLDL; Free cholesterol in large HDL; Free cholesterol in large VLDL; Free cholesterol in medium VLDL; Free cholesterol in small VLDL; Free cholesterol in very large HDL; Free cholesterol in very large VLDL; Free cholesterol to total lipids ratio in large HDL; Free cholesterol to total lipids ratio in large LDL; Free cholesterol to total lipids ratio in small HDL;** Glycoprotein acetyls; Gynoid bone mass; Gynoid fat free mass; Gynoid fat mass; Gynoid lean mass; Gynoid tissue mass; Gynoid total mass**; HDL cholesterol**; Hepatic Fat; **High-density lipoprotein cholesterol; Hip circumference;** Hip lower neck bone mineral content; Hip lower neck bone mineral density; Hip neck bone mineral content; Hip shaft bone mineral content; Hip shaft bone mineral density; Hip total bone mineral content; Hip total bone mineral density; Hip total T score; Hip Trochanter bone mineral density; **HOMA-IR**; **Hypertriglyceridemic waist**; **Insulin**; Isoleucine; Lactate; Leg bone mass; Leg bone mineral content; Leg fat free mass; Leg fat mass; Leg lean mass; Leg tissue mass; Leg total mass; M32322_glutamate; Mean diameter for HDL particles; Mean diameter for VLDL particles; **Monounsaturated fatty acids 16:1 18:1;** Nonalcoholic fatty liver disease; Pelvis bone mineral content; Pelvis bone mineral density; **Phospholipids in chylomicrons and extremely large VLDL; Phospholipids in large HDL; Phospholipids in large VLDL; Phospholipids in medium VLDL; Phospholipids in small VLDL; Phospholipids in very large HDL; Phospholipids in very large VLDL; Phospholipids to total lipids ratio in large HDL;** Postprandial lipemia; Pyruvate; **Ratio of apolipoprotein B to apolipoprotein A I; Ratio of omega 6 fatty acids to total fatty acids; Ratio of triglycerides to phosphoglycerides**; Ribs bone mineral density; **Saturated fatty acids**; **Serum high-density lipoprotein cholesterol**; **Serum total triglycerides**; **Serum triglycerides**; Sex; Spine area; Spine bone mineral content; Statin use; **Systolic Blood Pressure**; Tissue; Total body bone mass; Total body bone mineral content; Total body mass; **Total cholesterol**; **Total cholesterol in chylomicrons and extremely large VLDL; Total cholesterol in HDL; Total cholesterol in HDL2; Total cholesterol in large HDL; Total cholesterol in large VLDL; Total cholesterol in medium VLDL; Total cholesterol in small VLDL; Total cholesterol in very large HDL; Total cholesterol in very large VLDL; Total cholesterol in VLDL; Total cholesterol to total lipids ratio in large HDL; Total cholesterol to total lipids ratio in medium HDL; Total cholesterol to total lipids ratio in small VLDL; Total cholesterol to total lipids ratio in very small VLDL**;

			Total fat free mass; Total fat mass; **Total fatty acids**; Total lean mass; **Total lipids in chylomicrons and extremely large VLDL**; **Total lipids in large HDL; Total lipids in large VLDL; Total lipids in medium VLDL; Total lipids in small HDL; Total lipids in small VLDL; Total lipids in very large HDL; Total lipids in very large VLDL;** Total region fat; Total tissue fat; Total tissue mass; ; **Triglycerides; Triglycerides in chylomicrons and extremely large VLDL; Triglycerides in large VLDL; Triglycerides in medium HDL; Triglycerides in medium VLDL; Triglycerides in small HDL; Triglycerides in small VLDL; Triglycerides in very large VLDL; Triglycerides in very small VLDL; Triglycerides in VLDL; Triglycerides to total lipids ratio in medium HDL; Triglycerides to total lipids ratio in small HDL; Triglycerides to total lipids ratio in small VLDL; Triglycerides to total lipids ratio in very small VLDL;** Trunk bone mass; Trunk bone mineral content; Trunk bone mineral density; Trunk fat free mass; Trunk fat mass; Trunk lean mass; Trunk region fat; Trunk tissue fat; Trunk tissue mass; Trunk total mass; **Type II diabetes**; **VLDL cholesterol**; **Waist circumference**; Weight
cg11024682	0	*SREBF1*	Age; Age 4 vs age 0; Aging; **BMI**; **Body mass index**; **Cholesterol esters to total lipids ratio in large HDL; Concentration of small HDL particles;** Conjugated linoleic acid; **C-reactive protein**; **fasting glucose**; **fasting insulin**; **Free cholesterol to total lipids ratio in large HDL; Free cholesterol to total lipids ratio in large LDL;** Gamma-glutamyl transferase; **Glucose**; Hepatic Fat**; High-density lipoprotein cholesterol; Hypertriglyceridemic waist**; M35718_dihomo-linolenate_20:3n3_or_n6; **Phospholipids in small HDL; Phospholipids to total lipids ratio in large HDL; Postprandial lipemia; Ratio of 18:2 linoleic acid to total fatty acids; Ratio of omega 6 fatty acids to total fatty acids; Ratio of triglycerides to phosphoglycerides; Serum high-density lipoprotein cholesterol; Serum triglycerides**; Sex; Smoking; Tissue**; Total cholesterol to total lipids ratio in large HDL; Total lipids in small HDL; ; Triglycerides; Triglycerides in medium HDL; Triglycerides in small HDL; Triglycerides to total lipids ratio in medium HDL; Type II diabetes; Waist circumference**
cg00574958	0	*CPT1A*	Age; Alcohol consumption; Alcohol consumption per day; Arm region fat; **BMI**; **Body mass index**; **Body mass index change**; **Cardiovascular risk;** Conjugated linoleic acid; **Diastolic Blood Pressure**; **fasting glucose**; **fasting insulin**; Gamma-glutamyl transferase; Gestational age; Glycoprotein acetyls; Hepatic Fat; **Hypertriglyceridemic waist**; Isoleucine; Leucine; M33871_1-eicosadienoylglycerophosphocholine*; Plasma adiponectin; **Postprandial lipemia**; **Saturated fatty acids; Serum triglycerides**; Smoking; **Systolic Blood Pressure**; Tissue; ; **Triglycerides**; Triglycerides in medium HDL; Type II diabetes; Type II diabetes mellitus; Very low-density lipoprotein cholesterol; Waist circumference
cg05778424	0	*AKAP1*	**BMI**; **Body mass index**; M32388_dodecanedioate; Nitrogen dioxide exposure; Sex; Tissue; ; **Type II diabetes**
cg14476101	0	*PHGDH*	Age 4 vs age 0; Alcohol consumption; Alcohol consumption per day; **BMI**; **Body mass index**; **Cholesterol esters in chylomicrons and extremely large VLDL; Cholesterol esters in large VLDL; Cholesterol esters to total lipids ratio in medium HDL; Concentration of large VLDL particles; Concentration of medium VLDL particles; Concentration of small VLDL particles; C-reactive protein; Diastolic Blood Pressure**; **fasting glucose**; **Free cholesterol in large VLDL; Free cholesterol in medium VLDL; Free cholesterol in small VLDL; Free cholesterol to total lipids ratio in IDL; Free cholesterol to total lipids ratio in large VLDL;** Gamma-glutamyl transferase; Glycoprotein acetyls; Hepatic Fat; M32197_3-4-hydroxyphenyllactate; M37202_4-androsten-3betacomma17beta-diol_disulfate_1*; **Maternal overweight/obesity**; **Mean diameter for VLDL particles; Monounsaturated fatty acids 16:1 18:1**; Mortality; Nonalcoholic fatty liver disease; **Phospholipids in large VLDL; Phospholipids in medium VLDL; Phospholipids in small VLDL; Ratio of 18:2 linoleic acid to total fatty acids; Ratio of monounsaturated fatty acids to total fatty acids; Ratio of omega 6 fatty acids to total fatty acids; Ratio of polyunsaturated fatty acids to total fatty acids; Serum total triglycerides; Serum triglycerides;** Sex; **Systolic Blood Pressure**; Tissue; **Total cholesterol in large VLDL; Total cholesterol in medium VLDL; Total cholesterol to total lipids ratio in medium HDL; Total lipids in large VLDL; Total lipids in medium VLDL; Total lipids in small VLDL; ; Triglycerides; Triglycerides in HDL; Triglycerides in large VLDL; Triglycerides in medium HDL; Triglycerides in medium VLDL; Triglycerides in small HDL; Triglycerides in small VLDL; Triglycerides in VLDL; Triglycerides to total lipids ratio in medium HDL; Triglycerides to total lipids ratio in small VLDL; Type II diabetes; VLDL cholesterol; Waist circumference**

cg04816311	0	*C7orf50*	**BMI**; **Body mass index**; M32388_dodecanedioate; Primary Sjogrens syndrome; Schizophrenia; Tissue; **Type II diabetes**; **Waist circumference**
cg07504977	2319	*OLMALINC*	Age; **Body mass index**; Clear cell renal carcinoma; **Fasting glucose**; **fasting insulin**; Fetal vs adult liver; Gestational age; Particulate matter air pollution; **Serum triglycerides**; Smoking; Tissue; ; **Triglycerides**; **Waist circumference**
cg19750657	0	*UFM1*	Age 4 vs age 0; Birthweight; **BMI**; **Body mass index**; Gestational age; Plasma adiponectin; **Serum high-density lipoprotein cholesterol**; Tissue; ; **Type II diabetes mellitus**
cg06378491	0	*MAP4K2*	Birthweight; Alcohol consumption
cg14020176	0	*SLC9A3R1*	Age; **Body mass index**; **fasting insulin**; Gestational age; Hepatic Fat; M32388_dodecanedioate; Nonalcoholic fatty liver disease; Sex; Tissue; **Type II diabetes**
cg06397161	0	*SYNGR1*	Age 4 vs age 0; **BMI**; **Body mass index**; **fasting insulin**; Fetal vs adult liver; Gestational age; M31548_DSGEGDFXAEGGGVR*; Maternal smoking in pregnancy; Prenatal smoke exposure; **Serum high-density lipoprotein cholesterol**; Sex; Smoking; Tissue; ; **Type II diabetes**
cg06940720	2852	*LPCAT1*	Age 4 vs age 0; Alcohol consumption; **Body mass index**; Gestational age; Hypertensive disorders of pregnancy; Papuan ancestry
cg02711608	0	*SLC1A5*	Alcohol consumption; Alcohol consumption per day; **BMI**; **Body mass index**; **C-reactive protein**; **Diastolic Blood Pressure**; Gamma-glutamyl transferase; Gestational age; Hepatic Fat; **HOMA**-IR; M33364_gamma-glutamylthreonine*; Mortality; **Serum triglycerides**; Smoking; **Systolic Blood Pressure**; Tissue; ; **Type II diabetes**
cg06192883	0	*MYO5C*	Android region fat; Android tissue fat; **BMI**; **body mass index**; Coagulation factor VIII; **C-reactive protein;** Crohn's disease; **fasting insulin**; Fetal vs adult liver; Gestational age; Glycoprotein acetyls; Inflammatory bowel disease; **Insulin**; M32338_glycine; Primary Sjogrens syndrome; **Serum high-density lipoprotein cholesterol**; Tissue; ; **Waist circumference**
cg09664445	0	*CLUH*	Alcohol consumption; **BMI**; **Body mass index**; Fetal vs adult liver; Lung function decline; Major depressive disorder; Smoking; Tissue; ; **Waist circumference**
cg14870271	0	*LGALS3BP*	**BMI**; **Body mass index**; Crohn's disease; Gestational age; HIV infection; Multiple sclerosis; Primary Sjogrens syndrome; Tissue;
cg18568872	0	*ZNF710*	Alcohol consumption; **BMI**; **Body mass index**; **fasting insulin**; Fetal vs adult liver; Gestational age; Sex; Tissue; **Waist** circumference
cg12257439	0	*FER1L5*	Age 4 vs age 0; Crohn's disease; Fetal vs adult liver; HIV infection; Sex; Smoking
cg11269166	0	*METTL8*	Age 4 vs age 0
cg14956201	0	*TRIO*	HIV infection; Smoking
cg17540192	0	*TECPR1*	Chronic kidney disease; Smoking
cg27243685	0	*ABCG1*	**Body mass index**; Cholesterol esters to total lipids ratio in medium HDL; Cholesterol esters to total lipids ratio in very small VLDL; Concentration of small VLDL particles; Crohn's disease; **fasting insulin; Free cholesterol in small VLDL;** Hepatic Fat; **High-density lipoprotein cholesterol; Hypertriglyceridemic waist**; Inflammatory bowel disease; M35718_dihomo-linolenate_20:3n3_or_n6; Maternal smoking in pregnancy; Nonalcoholic fatty liver disease; **Phospholipids in small VLDL; Serum high-density lipoprotein cholesterol; Serum triglycerides; Statin use; Total body mass; Total cholesterol to total lipids ratio in large HDL; Total cholesterol to total lipids ratio in medium HDL; Total cholesterol to total lipids ratio in very small VLDL; Total lipids in small HDL; Total lipids in small VLDL;** Total tissue mass; **Triglycerides; Triglycerides in IDL; Triglycerides in medium HDL; Triglycerides in small HDL; Triglycerides in very small VLDL; Triglycerides to total lipids ratio in medium HDL;** Trunk tissue mass; Trunk total mass; **VLDL cholesterol**; **Waist circumference**
cg11202345	0	*LGALS3BP*	**BMI**; **Body mass index**; **fasting insulin**; Gestational age; Multiple sclerosis; Primary Sjogrens syndrome; Tissue;

cg15020801	0	*PNPO*	Age 4 vs age 0; Alcohol consumption per day; Birthweight; Cognitive abilities: Phonemic Verbal Fluency; **C-reactive protein;** Crohn's disease; **fasting insulin**; Inflammatory bowel disease; **Maternal body mass index**; **Maternal overweight/obesity**; Smoking; Tissue; ; Walking speed
cg21480264	0	*POLN*	Cleft palate vs cleft lip; Gestational age; Sex; Smoking
cg08788930	0	*DENND3*	**BMI**; **High-density lipoprotein cholesterol**; HIV infection; Papuan ancestry; **Serum high-density lipoprotein cholesterol**; Sex; Smoking; Tissue;
cg25217710	2215	*BCAN*	**BMI**; **Body mass index**; **C-reactive protein**; Crohn's disease; **fasting insulin**; **High-density lipoprotein cholesterol**; HIV infection; Inflammatory bowel disease; Perinatally acquired HIV;
cg22650271	0	*SYNGR1*	**BMI**; **Body mass index**; **fasting insulin**; Fetal vs adult liver; Hypertensive disorders of pregnancy; Prenatal smoke exposure; Sex; Smoking; Tissue;
cg10639435	0	*ZNF250*	Alcohol consumption; Clear cell renal carcinoma; Gestational age; Gestational age; **Maternal overweight/obesity**; Tissue
cg01101459	4086	*LINC01132*	**BMI**; **Body mass index**; **C-reactive protein**; Crohn's disease; Gestational age; **High-density lipoprotein cholesterol**; HIV infection; Inflammatory bowel disease; Rheumatoid arthritis; **Serum high-density lipoprotein cholesterol**; Soluble tumor necrosis factor receptor 2; Tissue; Ulcerative colitis
cg03691549	0	*LOC283335*	Age; Age 4 vs age 0; Birthweight; Gestational age; Primary Sjogrens syndrome; Tissue
cg26262157	0	*PFKFB3*	Age; Alcohol consumption; Alcohol consumption per day; Clear cell renal carcinoma; Cleft palate vs cleft lip; Crohn's disease; Gestational age; **HbA1c**; HIV infection; Tissue
cg04927537	29	*LGALS3BP*	Arm total mass; Arms tissue mass; Autoantibody production in systemic lupus erythematosus; **BMI**; **Body mass index**; Crohn's disease; **fasting glucose**; **fasting insulin**; Gestational age; Multiple sclerosis; Pancreatic ductal adenocarcinoma; Tissue; **Waist circumference**
cg08994060	0	*PFKFB3*	Age; Clear cell renal carcinoma; Cleft palate vs cleft lip; Gestational age; HIV infection; Tissue; **Type II diabetes**
cg13059136	0	*NAP1L4*	Alcohol consumption per day; **body mass index**; **body mass index**; **C-reactive protein**; Fetal vs adult liver; HIV infection; M35718_dihomo-linolenate_20:3n3_or_n6; Nitrogen dioxide exposure; Tissue
cg21234053	16166	*CFL2*	Maternal smoking in pregnancy; Smoking
cg08309687	0	*LINC00649*	Age 4 vs age 0; **BMI**; **Body mass index**; **fasting glucose**; **fasting insulin**; Hepatic Fat; **Hypertriglyceridemic waist**; Nonalcoholic fatty liver disease; **Ratio of monounsaturated fatty acids to total fatty acids;** Sex; Smoking; Tissue; **Triglycerides to total lipids ratio in very large HDL; Type II diabetes**; **Waist circumference**
cg02879453	0	*ADCY7*	Crohn's disease; Gestational age; Hypertensive disorders of pregnancy; Inflammatory bowel disease; Preeclampsia; **Serum high-density lipoprotein cholesterol**; Sex; Ulcerative colitis
cg24259291	0	*ZNFX1*	**C-reactive protein**; Crohn's disease; Gestational age; Nitrogen dioxide exposure; Smoking
cg26846781	0	*KCNH6*	Age 4 vs age 0; Alcohol consumption; Alcohol consumption per day; **C-reactive protein**; Crohn's disease; Tissue
cg16097041	0	*FLAD1*	Birthweight; **Body mass index; C-reactive protein;** Crohn's disease; Prenatal smoke exposure; Tissue; **Waist circumference**
cg01373896	0	*KLF16*	Alcohol consumption; Fetal vs adult liver; Gestational age; Smoking; Tissue
cg19169154	0	*MFAP4*	Alcohol consumption; Alcohol consumption per day; Cleft palate vs cleft lip; Fetal intolerance of labor; Gestational age; Hypertensive disorders of pregnancy; Tissue
cg13300580	0	*SLC9A1*	Age; **C-reactive protein;** Gestational age; Primary Sjogrens syndrome; Rheumatoid arthritis; Tissue
cg23021329	0	*TLR9*	Tissue

cg25001190	0	*NFIA*	**BMI**; **Body mass index**; Chronic kidney disease; **fasting insulin**; Fetal vs adult liver; Gestational age; Hypertensive disorders of pregnancy; **Phospholipids to total lipids ratio in large HDL**; Preeclampsia; Tissue
cg02050917	0	*SKI*	**BMI**; **Body mass index**; **C-reactive protein**; Fetal vs adult liver; Gestational age; HIV infection; **Maternal body mass index**; **Maternal overweight/obesity**; Sex; Tissue
cg07719604	0	*E2F4*	Age 4 vs age 0; Gestational age
cg26663590	2816	*NFATC2IP*	**Body mass index**; **C-reactive protein;** Crohn's disease; Gestational age; Inflammatory bowel disease; **Maternal body mass index**; Nitrogen dioxide exposure; Pancreatic ductal adenocarcinoma; Tissue; **Waist circumference**;
cg17836612	295	*LGALS3BP*	**Body mass index**; Gestational age; Multiple sclerosis; Sex; Tissue
cg20507228	0	*MAN2A2*	Birthweight; **Body mass index**; **fasting insulin**; Gestational age; Leukocyte telomere length; M12774_X-03094; Tissue
cg04682775	0	*SLC6A9*	Age 4 vs age 0; sex
cg24145109	115779	*MIR4689*	Age 4 vs age 0; **BMI**; **Body mass index**; Gestational age; M32338_glycine; Sex; Tissue
cg10192877	0	*ABCG1*	**Body mass index**; Gestational age; HIV infection; **Hypertriglyceridemic waist**
cg21703988	0	*EP400*	Birthweight; Gestational age; HIV infection; Smoking; Tissue
cg17901584	784	*DHCR24*	Android fat free mass; Android lean mass; Android tissue mass; Android total mass; Birthweight; **BMI**; **Body mass index**; **Cholesterol esters in large HDL; Cholesterol esters in very large HDL; Cholesterol esters to total lipids ratio in large HDL; Concentration of large HDL particles; Concentration of small VLDL particles; Concentration of very large HDL particles; C-Reactive Protein;** Crohn's disease; **fasting insulin**; Fetal vs adult liver; **Free cholesterol in large HDL; Free cholesterol in very large HDL; Free cholesterol to total lipids ratio in large LDL; Free cholesterol to total lipids ratio in small HDL**; Gestational age; Glycoprotein acetyls; **HDL cholesterol**; Healthy eating; Hepatic Fat; **High-density lipoprotein cholesterol**; Hip shaft bone mineral content; Hip total bone mineral content; Hypertensive disorders of pregnancy; Inflammatory bowel disease; **Insulin**; Isoleucine; M32492_caprylate_8:0; **Mean diameter for HDL particles; Phospholipids in large HDL; Phospholipids in small VLDL; Phospholipids in very large HDL; Phospholipids to total lipids ratio in large HDL; Preeclampsia; Primary Sjogrens syndrome; Ratio of apolipoprotein B to apolipoprotein A I**; Ribs bone mineral density; Schizophrenia; **Serum high-density lipoprotein cholesterol; Serum total cholesterol; Serum triglycerides**; Sex; Statin use; Tissue; Total body mass; **Total cholesterol in HDL; Total cholesterol in HDL2; Total cholesterol in large HDL; Total cholesterol in very large HDL; Total cholesterol to total lipids ratio in large HDL; Total fat free mass; Total lean mass; Total lipids in large HDL; Total lipids in small VLDL; Total lipids in very large HDL;** Total tissue mass; ; **Triglycerides**; **Triglycerides in large HDL; Triglycerides in small VLDL; Triglycerides in VLDL;** Trunk bone mineral density; Trunk tissue mass; Trunk total mass; Ulcerative colitis; **Waist circumference**; Weight
cg25178683	205	*LGALS3BP*	**BMI**; **Body mass index**; Crohn's disease; Gestational age; Multiple sclerosis; Primary Sjogrens syndrome; Tissue; **Waist circumference**
cg25130381	0	*SLC9A1*	Birthweight; **BMI**; Crohn's disease; **fasting glucose**; Gestational age; Leisure time physical activity; Primary Sjogrens syndrome; **Serum high-density lipoprotein cholesterol**; Smoking; Tissue; **Type II diabetes**
cg25649826	0	*USP22*	Alcohol consumption; **BMI**; **Body mass index**; Smoking; Tissue
cg20212624	0	*CNP*	Age 4 vs age 0; Age; Sex; Crohn's disease; Inflammatory bowel disease
cg07567724	0	*GATAD2B*	Age 4 vs age 0; Alcohol consumption; Alcohol consumption per day; **Free cholesterol in large HDL**; Hip upper neck Z score; Hip Ward's Triangle Z score; **Serum high-density lipoprotein cholesterol**; Tissue; **Total cholesterol in large HDL**
cg16861241	0	*RNF157-AS1*	Age 4 vs age 0; Clear cell renal carcinoma; Fetal vs adult liver; Hypertensive disorders of pregnancy; Prenatal smoke exposure; Primary Sjogrens syndrome; Sex; Smoking; Tissue
cg03819286	849	*MGRN1*	Gestational age; Inflammatory bowel disease; Nitrogen dioxide exposure; Perinatally acquired HIV; Preeclampsia; Tissue

cg02079413	0	*NAP1L4*	**BMI**; **Body mass index**; HIV infection; Mediterranean diet; Nitrogen dioxide exposure; Tissue
cg23722778	0	*ENPP4*	Tissue
cg11800635	0	*DOK1*	Alcohol consumption; Clear cell renal carcinoma; Fetal vs adult liver; Gestational age; M32388_dodecanedioate; Smoking
cg25316512	131	*ENO2*	Preeclampsia; Tissue
cg09294084	0	*MCF2L*	Age; Alcohol consumption; Birthweight; Clear cell renal carcinoma; Fetal vs adult liver; Gestational age; M32388_dodecanedioate; **Maternal body mass index**; Papuan ancestry; Tissue
cg20784591	0	*PILRA*	Alcohol consumption; Birthweight; **fasting insulin**; Gestational age; Inflammatory bowel disease; Tissue
cg03497652	0	*ANKS3*	Alcohol consumption per day; Clear cell renal carcinoma; Crohn's disease; Fetal vs adult liver; Gamma-glutamyl transferase; HIV infection; M15749_3-phenylpropionate_hydrocinnamate; Pancreatic ductal adenocarcinoma; Schizophrenia; Smoking; Tissue
cg24678869	483	*DENND4B*	Alcohol consumption; Alcohol consumption per day; **Body mass index**; Crohn's disease; **fasting insulin**; Inflammatory bowel disease; Preeclampsia; **Serum high-density lipoprotein cholesterol**; Sex; Tissue; **Waist circumference**
cg12322877	0	*ASPSCR1*	Age 4 vs age 0; Alcohol consumption; Maternal smoking in pregnancy; Prenatal smoke exposure; Smoking; Tissue
cg09072148	978	*NRXN2*	Melanoma; Tissue
cg14524754	0	*B3GNTL1*	Gestational age
cg17194270	0	*SYNGR1*	**BMI**; **Body mass index**; **fasting insulin**; Gestational age; M32388_dodecanedioate; Prenatal smoke exposure; Sex; Smoking; Tissue

ESM Table 13. List of 76 significant DMS associated with incident T2D and correlation with gene expression levels in blood (data from BIOS consortium).

Illumina ID	Gene name	Expression Probe Name	Expression Gene Name	Included Datasets Correlation Coefficient	Beta (SE)	FDR^
cg19693031 *	*TXNIP*	ENSG00000117289	TXNIP	-0.1220719;-0.1416876;-0.1070264;-0.053735	-0.1220719 (0.03902);-0.1416876 (0.0397879);-0.1070264 (0.0391488);-0.053735 (0.0740178)	9.28E-06
cg06500161 *	*ABCG1*	ENSG00000160179	ABCG1	-0.3216932;-0.2638823;-0.2659009;-0.1624013	-0.3216932 (0.0372243);-0.2638823 (0.0387687);-0.2659009 (0.0379575);-0.1624013 (0.0731409)	0
cg11024682 *	*SREBF1*	ENSG00000072310	SREBF1	-0.1996705;-0.1501603;-0.1822975;-0.0897884	-0.1996705 (0.0385224);-0.1501603 (0.0397377);-0.1822975 (0.0387152);-0.0897884 (0.0738255)	0
cg00574958 *	*CPT1A*	ENSG00000110090	CPT1A	-0.1720518;-0.1980765;-0.2377033;-0.1654026	-0.1720518 (0.0387278);-0.1980765 (0.039397);-0.2377033 (0.0382464);-0.1654026 (0.0731039)	0
cg05778424	*AKAP1*					
cg14476101 *	*PHGDH*	ENSG00000092621	PHGDH	0.3413303;0.3387843;0.3008326;0.396035	0.3413303 (0.036953);0.3387843 (0.0378165);0.3008326 (0.037551);0.396035 (0.0680641)	0
cg04816311 *	*C7orf50*	ENSG00000146540	C7orf50	-0.2535105;-0.1980662;-0.1451129;-0.3229671	-0.2535105 (0.0380298);-0.1980662 (0.0393971);-0.1451129 (0.0389582);-0.3229671 (0.0701526)	0
cg07504977	*OLMALINC*					
cg19750657	*UFM1*					
cg06378491	*MAP4K2*					
cg14020176	*SLC9A3R1*					
cg06397161	*SYNGR1*					
cg06940720	*LPCAT1*					
cg02711608 *	*SLC1A5*	ENSG00000105281	SLC1A5	0.0730841;0.0707482;0.0935482;0.1304718	0.0730841 (0.0392089);0.0707482 (0.0400927);0.0935482 (0.0392023);0.1304718 (0.0734913)	0.0237
cg06192883	*MYO5C*					
cg09664445 *	*CLUH*	ENSG00000132361	CLUH	0.2089625;-0.0162153;0.0719327;0.0367001	0.2089625 (0.0384461);-0.0162153 (0.0401881);0.0719327 (0.039273);0.0367001 (0.074075)	0.0175
cg14870271 *	*LGALS3BP*	ENSG00000108679	LGALS3BP	-0.4217967;-0.5144115;-0.5177105;-0.4579425	-0.4217967 (0.0356457);-0.5144115 (0.0344676);-0.5177105 (0.0336875);-0.4579425 (0.0658957)	0
cg18568872	*ZNF710*					
cg12257439	*FER1L5*					

cg11269166	*METTL8*					
cg14956201	*TRIO*					
cg17540192	*TECPR1*					
cg27243685 *	*ABCG1*	ENSG00000160179	ABCG1	-0.2180342;-0.1906523;-0.218115;-0.1550554	-0.2180342 (0.0383682);-0.1906523 (0.0394562);-0.218115 (0.0384269);-0.1550554 (0.0732284)	0
cg11202345 *	*LGALS3BP*	ENSG00000108679	LGALS3BP	-0.3803724;-0.5366327;-0.4540323;-0.443103	-0.3803724 (0.036359);-0.5366327 (0.0339158);-0.4540323 (0.0350825);-0.443103 (0.0664508)	0
cg15020801	*PNPO*					
cg21480264	*POLN*					
cg08788930 *	*DENND3*	ENSG00000184489	PTP4A3	0.0696183;0.0707496;0.13699;0.167737	0.0696183 (0.0392187);0.0707496 (0.0400927);0.13699 (0.0390038);0.167737 (0.0730747)	0.0015
cg25217710	*BCAN*					
cg22650271 *	*SYNGR1*	ENSG00000100321	SYNGR1	0.0564824;0.1334969;0.1688589;-0.0245851	0.0564824 (0.0392513);0.1334969 (0.0398336);0.1688589 (0.0388095);-0.0245851 (0.0741025)	2.96E-04
cg10639435	*ZNF250*					
cg01101459	*LINC01132*					
cg03691549	*LOC283335*					
cg26262157	*PFKFB3*					
cg04927537 *	*LGALS3BP*	ENSG00000108679	LGALS3BP	-0.4869259;-0.5686569;-0.5395161;-0.5443166	-0.4869259 (0.0343386);-0.5686569 (0.0330621);-0.5395161 (0.0331527);-0.5443166 (0.0621819)	0
cg08994060	*PFKFB3*					
cg13059136	*NAP1L4*					
cg21234053	*CFL2*					
cg08309687 *	*LINC00649*	ENSG00000237945	LINC00649	0.0930589;0.123794;0.0710863;0.2058276	0.0930589 (0.0391435);0.123794 (0.0398842);0.0710863 (0.0392753);0.2058276 (0.0725378)	4.67E-04
cg02879453	*ADCY7*					
cg24259291	*ZNFX1*					
cg26846781	*KCNH6*					
cg16097041	*FLAD1*					
cg01373896	*KLF16*					
cg19169154	*MFAP4*					
cg13300580	*SLC9A1*					
cg23021329	*TLR9*					

cg25001190	*NFIA*					
cg02050917	*SKI*					
cg07719604	*E2F4*					
cg26663590	*NFATC2IP*					
cg17836612 *	*LGALS3BP*	ENSG00000108679	LGALS3BP	-0.4095977;-0.4845898;-0.4882958;-0.4242982	-0.4095977 (0.0358649);-0.4845898 (0.0351588);-0.4882958 (0.0343617);-0.4242982 (0.0671218)	0
cg20507228	*MAN2A2*					
cg04682775	*SLC6A9*					
cg24145109	*MIR4689*					
cg10192877 *	*ABCG1*	ENSG00000160179	ABCG1	-0.1224008;-0.1219994;-0.1529052;-0.0697461	-0.1224008 (0.0390184);-0.1219994 (0.0398932);-0.1529052 (0.0389119);-0.0697461 (0.0739444)	0
cg21703988	*EP400*					
cg17901584	*DHCR24*					
cg25178683 *	*LGALS3BP*	ENSG00000108679	LGALS3BP	-0.4848122;-0.5234105;-0.5122536;-0.5278986	-0.4848122 (0.0343848);-0.5234105 (0.034248);-0.5122536 (0.0338166);-0.5278986 (0.0629549)	0
cg25130381	*SLC9A1*					
cg25649826	*USP22*					
cg20212624	*CNP*					
cg07567724	*GATAD2B*					
cg16861241	*RNF157-AS1*					
cg03819286	*MGRN1*					
cg02079413	*NAP1L4*					
cg23722778	*ENPP4*					
cg11800635 *	*DOK1*	ENSG00000115318	LOXL3	-0.144213;-0.1386601;-0.223081;-0.167773	-0.144213 (0.0389031);-0.1386601 (0.0398051);-0.223081 (0.0383827);-0.167773 (0.0730743)	0
cg11800635 *	*ENO2*	ENSG00000115325	DOK1	-0.1884945;-0.1501819;-0.1240971;-0.2351036	-0.1884945 (0.0386093);-0.1501819 (0.0397375);-0.1240971 (0.0390706);-0.2351036 (0.0720472)	0
cg11800635 *	*MCF2L*	ENSG00000159374	M1AP	-0.0820888;-0.1433118;-0.1149946;-0.1303683	-0.0820888 (0.0391814);-0.1433118 (0.0397785);-0.1149946 (0.0391138);-0.1303683 (0.0734923)	6.16E-05
cg25316512	*PILRA*					
cg09294084	*ANKS3*					
cg20784591	*DENND4B*					
cg03497652 *	*ASPSCR1*	ENSG00000118900	UBN1	0.0639811;0.1064521;0.09854;0.0306685	0.0639811 (0.0392335);0.1064521 (0.039965);0.09854 (0.0391833);0.0306685 (0.0740901)	0.0222

cg24678869	*NRXN2*					
cg12322877	*B3GNTL1*					
cg09072148	*SYNGR1*					
cg14524754	*B3GNTL1*					
cg17194270	*SYNGR1*	ENSG00000100321	SYNGR1	-0.0324386;-0.0843447;-0.1190213;-0.1998127	-0.0324386 (0.0392934);-0.0843447 (0.0400502);-0.1190213 (0.0390951);-0.1998127 (0.0726301)	0.01

*Significant correlation between DNA methylation level and expression level

^FDR=0 is <1x10-7

ESM Table 14. List of significant mQTLs from blood (http://mqtldb.godmc.org.uk/) based on 76 DMS associated with incident T2D.

rsid	a1	a2	name	cpg	beta_a1	se	Sample size	pval	Het isq	direction
rs141889270	A	G	chr19:1870952:SNP	cg01373896	0.1734365	0.0147139	22533	0	40.2	+++++++++++++---++???++??++?++?+++-+
rs9928014	C	T	chr1:2029207:SNP	cg02050917	-0.1493565	0.0098112	20636	0	18.2	+++++++?++++++?+++++++???+??++++++++
rs143827048	A	G	chr11:2957414:SNP	cg02079413	0.3662985	0.0091015	23725	0	58.6	+++++++?+?++++++++++++++?+??++++++++
rs9916764	T	A	chr11:2947903:SNP	cg02079413	-0.3795306	0.0091531	22350	0	45.7	++++++??+?+++++++++++++??+??++++++++
rs192437266	C	T	chr16:50376796:SNP	cg02879453	0.0969301	0.0086706	26179	5,16E-26	32.8	--+---+------------??-?-----------
rs4796807	T	C	chr12:53440779:SNP	cg03691549	0.20622	0.0163288	24606	1,46E-33	37.4	++++++?++++++-+++++++++??-??++++++-+
rs111232194	A	C	chr1:44508975:SNP	cg04682775	-0.3210329	0.0193784	23735	1,22E-58	0	------?-----------?----?-?------?--
rs34733337	G	C	chr7:1154153:SNP	cg04816311	-0.1758843	0.0149446	12425	5,64E-29	58.5	+++?+?+?+?++++?+++???????+??+??++?++
rs6952546	A	G	chr7:988009:SNP	cg04816311	0.6383517	0.0141284	17081	0	77.9	+++?++??++++++?+++??+?+??+??+??++?++
rs381996	T	C	chr17:76802648:SNP	cg04927537	0.1899647	0.0119167	20795	3,28E-54	56.7	++++++?++++-++++++?+++?+?+??+++++?++
rs384490	C	G	chr17:76990346:SNP	cg04927537	-0.4538296	0.0425956	17041	1,66E-23	0	----????-----+??--??-?-??-????---??-
rs2160115	C	T	chr17:55165612:SNP	cg05778424	-0.2333672	0.0094024	24549	5,46E-136	37.5	++++++?+++++++++++++++++?++++++?+?++
rs2007854	A	G	chr15:52528507:SNP	cg06192883	0.2136376	0.0219195	25664	1,91E-22	6.2	++++++?++++-+-+++-++++++?-?+++++++?+
rs630966	C	G	chr11:64524911:SNP	cg06378491	0.2207331	0.0150409	26371	9,25E-46	28.5	++++++?-++++++++++++++++?+++++++++++
rs971964	T	C	chr22:39702473:SNP	cg06397161	-0.2412295	0.0112792	23908	0	62.6	------?----?+?+---------??-------?--
rs7716426	T	C	chr5:1521573:SNP	cg06940720	-0.5375911	0.0132351	27253	0	58.8	----------------------?----------
rs3961637	A	C	chr10:102099802:SNP	cg07504977	-0.0971346	0.0091069	25161	0	31	-------?--------+-------?+??--------
rs6668066	A	G	chr1:154277215:SNP	cg07567724	0.1224955	0.0132325	22857	2,10E-17	39.6	++++-+?++++?++++-+-+++++????+++++?-+
chr12:7001232:I	T	C	chr1:154134022:SNP	cg07567724	0.3354189	0.0150768	17779	1,20E-109	27.4	+++?++??+?+??+++++?+??++????+++++?++
ss1388027821	A	C	chr1:153755451:SNP	cg07567724	0.5026076	0.0125012	13002	0	71.9	?++??+????+??++?++++??++?????++???++
chr16:29279157:I	I	D	chr16:67014421:INDEL	cg07719604	0.0895628	0.0173753	12144	0	50.3	?-+??-????--?-??+---??--?+????-???--

rs181203	G	A	chr16:66999370:SNP	cg07719604	0.079903	0.0122032	24099	0	41.4	--+-+++---+++-?-+--------+??-----?--
rs113924526	T	C	chr21:35260544:SNP	cg08309687	-0.3334434	0.0328509	17253	3,31E-21	34.4	------????--?+-?---??--??-???----??-
rs13044985	T	C	chr21:35303766:SNP	cg08309687	0.2225705	0.0150588	25584	0	25.2	+++++++++++-+++++++?++++?++++++?-+++
rs10087637	D	I	chr8:142317630:INDEL	cg08788930	0.1275342	0.0175767	15666	3,99E-10	32.4	?-+??++???+-?++?-+?-??+-?+???++??+-+
rs58092133	T	C	chr8:142198315:SNP	cg08788930	0.5855469	0.012597	22243	0	65.6	+++?+++?+?+++++?+++???++?++++++++++
rs426961	C	T	chr10:6039371:SNP	cg08994060	-0.1043185	0.0127436	23992	2,70E-13	30	++++-+?-+++++++--+--++++?+??-++++?-+
chr14:35166053:D	G	C	chr10:6201973:SNP	cg08994060	-0.7925128	0.0112976	18296	0	89.3	++++??+?+?++++++++?+++?+?+??+++++?++
rs4986085	T	C	chr13:113613988:SNP	cg09294084	0.8885319	0.0414098	7526	0	69.6	?++?+?+???++???+?+???????+?????+??++
rs2240730	G	A	chr17:2594218:SNP	cg09664445	0.1090665	0.0094613	23362	9,57E-28	4.4	------------+----????-?------?---+-
rs11654894	G	T	chr21:43619665:SNP	cg10192877	0.1086181	0.0106373	26396	1,77E-24	14.7	--+---?------+---++-----?-----------
rs79259094	G	A	chr8:145756170:SNP	cg10639435	-0.1330071	0.0130344	12026	0	50.8	+++?+???+?+++??+++??+????+??+??++?+-
rs11652574	A	G	chr17:17728815:SNP	cg11024682	10.954.911	0.027558	19085	0	66.8	+++++??+++++++?+++??+?+??+???+?+++++
rs4861	T	C	chr17:76802648:SNP	cg11202345	0.1574004	0.0119431	20795	1,16E-36	48.3	++++++?++++-+++-++?+++?+?+??+++++?-+
rs12989804	C	T	chr2:172209562:SNP	cg11269166	0.1569464	0.0091381	27000	4,09E-66	33.9	------------------------------?--++
rs116411618	T	C	chr2:74516738:SNP	cg11800635	0.921861	0.0448917	13604	1,04E-93	44.5	+++?++?+??+?+??+?++++??+?????++???-+
rs11867174	T	C	chr2:75062779:SNP	cg11800635	0.3925895	0.0348823	12652	2,20E-26	33.3	+++?++????+?+?+++++?????????++?++?-+
rs9912553	C	G	chr17:79959703:SNP	cg12322877	-0.3944402	0.0104145	21706	6,85e-314	67.9	--------?---------?---???-??--------
rs12878970	T	C	chr11:2953723:SNP	cg13059136	-0.5609855	0.0103181	22487	0	65.3	-------?-?----------?--??-??--------
rs145391487	A	G	chr11:2936846:SNP	cg13059136	-0.1024537	0.0087642	26395	1,43E-28	0	------------+----??--------------+-
rs2305214	A	G	chr17:72768970:SNP	cg14020176	0.1907205	0.0088198	25693	1,06E-103	26.8	+++++++++++++++++?++?++++??++++++++
rs347903	C	T	chr1:120192431:SNP	cg14476101	-0.2341405	0.0094823	24554	0	59.5	+++++++?+?+++-++++?+++++?+++++++++++
rs608358	A	C	chr1:120289407:SNP	cg14476101	-0.2767749	0.0096758	25566	5,82E-180	58.7	------??----------------?-----------
rs67334210	G	A	chr17:80940371:SNP	cg14524754	-0.1825263	0.0106045	22456	2,16E-63	0	+++++++???++?++++++++?++?++++++?++++
rs78165269	C	G	chr17:80601498:SNP	cg14524754	0.0677154	0.0098966	24169	0	35.4	++++++++?+++?-+++++++?++?-??+++--+++

rs117549034	T	C	chr17:76826416:SNP	cg14870271	-10.139.575	0.0585517	11141	3,48E-64	60.4	----?-?-?-??-?????--???????-?-???-??
rs116519591	C	T	chr5:14358001:SNP	cg14956201	-0.984487	0.0426323	16419	5,50E-118	55.6	+++?+????+?+?++++++++??+?+??+++++?++
rs153836	G	T	chr5:14661908:SNP	cg14956201	-0.1943232	0.0225112	22901	6,01E-15	0	+++++-??++++?+++-+++++++?++?+++++?+-
rs113090925	T	C	chr17:45892783:SNP	cg15020801	-0.3016997	0.0362573	16328	0	6.6	---?-?-?-----+??--????-??-??-??-+?--
rs12949191	A	C	chr17:46259254:SNP	cg15020801	0.1450628	0.0100241	27241	0	5.6	+++++++++++++-+++++++++?++++++++++++
rs137612	A	G	chr22:39698010:SNP	cg17194270	-0.325371	0.0085338	26894	4,54e-318	65.9	-------------------?--?----------
rs5750808	G	A	chr22:39790987:SNP	cg17194270	-0.1890572	0.0093289	26462	2,58E-91	30.2	+++++++++++++++++?++?++?+++++++++++
rs35983874	T	C	chr7:97916461:SNP	cg17540192	0.113544	0.0084708	27726	5,71E-38	5.4	+++++++++++++++++++++++-++++-+++++++
rs582222	T	C	chr17:76986095:SNP	cg17836612	-0.2784471	0.0310188	21434	2,79E-19	0	------??-----+----??-?--?-??-----??-
rs590565	T	C	chr17:76820580:SNP	cg17836612	0.3240853	0.0208211	24666	1,25E-51	18.8	++++++?++++-++++++?+++++?+??++++++-+
chr1:153726432:I	C	T	chr1:55354335:SNP	cg17901584	0.1877915	0.0085531	27749	7,60E-107	32.2	-----------------------------------
rs1256844	G	A	chr15:90478104:SNP	cg18568872	-0.1234484	0.0185027	20481	0	35.6	+++++-??-+++?-?-?+?++++-?+?++++?-?++
rs6496620	T	C	chr15:90617539:SNP	cg18568872	0.1782476	0.0104735	23770	0	31.9	+++++++?+?++++++-+++?++??+++++++++++
rs144026838	T	C	chr17:19181113:SNP	cg19169154	12.891.783	0.0593454	8076	1,24E-104	84.6	+??+????++++?????+???????+????????-+
chr17:20937066:I	T	G	chr15:91455960:SNP	cg20507228	0.3061873	0.0163233	25623	1,68E-75	8.3	++++++++++++++++++++?++?+??++++++++
rs607939	D	I	chr7:100037900:INDEL	cg20784591	-0.1335965	0.0138669	16769	0	66.2	?-+??--???--?+-?---+??--?-?-?--??---
rs58803997	I	D	chr14:35143811:INDEL	cg21234053	-0.2500774	0.0140462	18341	0	48.9	+++?++??+?+?+?++-++++?+?????+++?+?++
rs35257581	C	T	chr14:35505520:SNP	cg21234053	0.1635364	0.0135386	20318	1,36E-30	0	---?--?--?-?-?-------?--????+----?--
rs9993696	T	C	chr4:2189131:SNP	cg21480264	-0.1885304	0.013679	27749	0	39	--+---------+-+--------------+----
rs7215006	T	C	chr12:132414086:SNP	cg21703988	-0.2113252	0.0181217	23279	2,01E-28	13.9	----+---?---?-?-+-------?-??------+-
rs2727236	C	A	chr22:39661032:SNP	cg22650271	-0.2094828	0.0091059	23725	0	38.7	+++++++?+?+++++++++++++?+??++++++++
rs6095197	A	G	chr20:47786190:SNP	cg24259291	-0.3095617	0.0091483	27742	0	50.8	-----------------------------------
rs71351945	A	G	chr20:47302140:SNP	cg24259291	0.1338313	0.0110252	27257	0	21.4	+++++++++++-+++-+++++++++?+++++++++++
rs12028398	T	C	chr1:153883283:SNP	cg24678869	-0.1808338	0.0093201	26888	7,34E-81	41.9	------?------+---------------------

rs147343788	C	T	chr1:61664841:SNP	cg25001190	-0.1744704	0.0116124	20896	0	49.2	+++++++???+++++?++++?++??+??+++?++++
chr1:154016883:I	A	G	chr17:76989782:SNP	cg25178683	-0.3537538	0.0309049	21436	2,45E-27	0	------??--------+-??-?--?-??----+??-
chr1:154115677:I	T	C	chr17:76820580:SNP	cg25178683	0.3341057	0.0208172	24666	5,77E-55	16.7	++++++?++++-++++++?+++++?+??++++++++
rs2264575	G	A	chr12:7015915:SNP	cg25316512	-0.3077312	0.0092511	27750	1,27E-242	79.6	++++++++++++-++++++++++++++++++++++
rs10797049	A	G	chr17:20851985:SNP	cg25649826	-0.5230291	0.011683	23653	0	60.9	-------?-?--------??----?-----------
rs12032791	A	G	chr17:21170907:SNP	cg25649826	-0.0948365	0.0097003	23823	1,42E-19	0	-------?-?------------??--?-------+
chr11:2983340:D	C	T	chr10:6039371:SNP	cg26262157	-0.0906295	0.0127459	23992	1,16E-09	32.2	++++-+?-+++++++--+-+++++?-??-++++?-+
rs2898894	G	C	chr10:6201973:SNP	cg26262157	-0.7925194	0.0113074	18296	0	87.5	++++??+?+?++++++++?+++?+?+??+++++?++
rs14484	G	A	chr16:28340945:SNP	cg26663590	0.1287569	0.0122401	13546	0	51.5	---?-?-?-?----?---???????+??-??---+-
rs2290593	C	T	chr16:29265129:SNP	cg26663590	-0.1067373	0.0126481	13942	0	40.4	+++?+?+?+??+++?+++????+??+??-??++?-+
chr21:43614481:I	I	D	chr21:43614481:INDEL	cg27243685	-0.2591751	0.0198826	16051	0	0	?++??++???++?++?++++??++?+?+??+??+++

ESM Figure 1. QQ plots per cohort from meta-analysis on incident type 2 diabetes. Order of the plots- Doetinchem cohort, ESTHER, KORA1, KORA2, EPIC-Norfolk and total meta-analysis model1; total meta-analysis model 1.1; total meta-analysis model 2, total meta-analysis model 2.1.

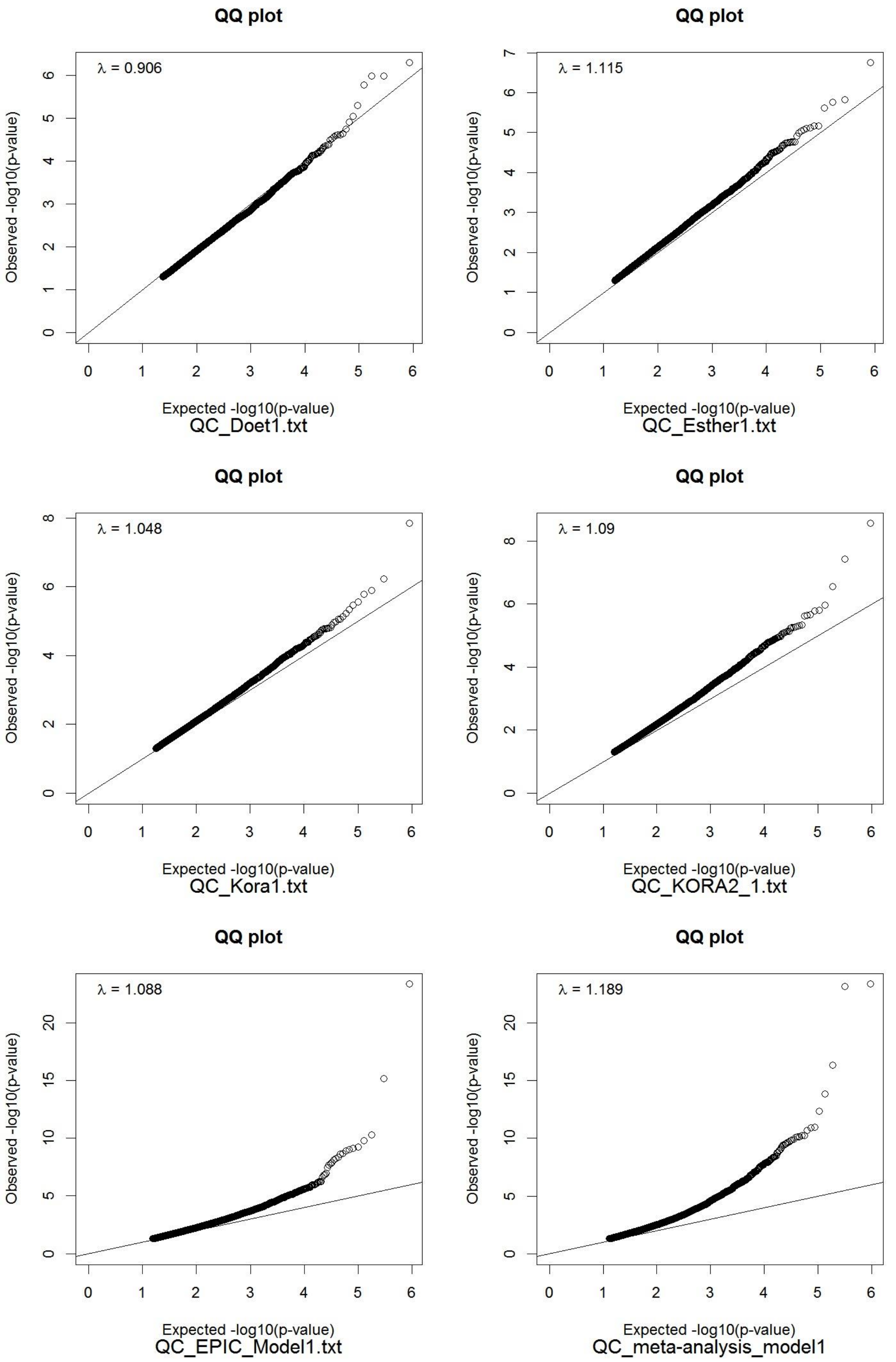

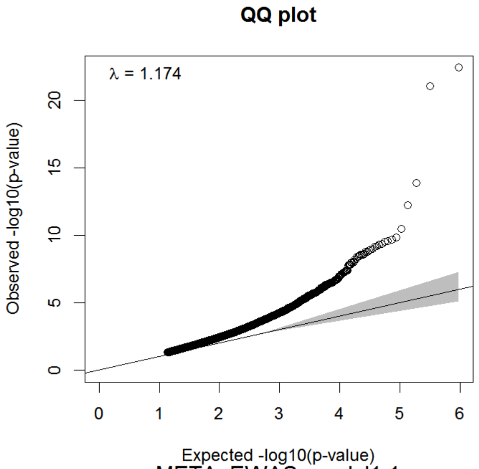

META_EWAS_model1.1

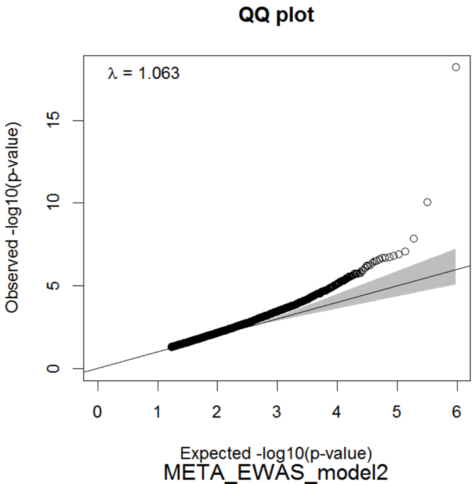

META_EWAS_model2

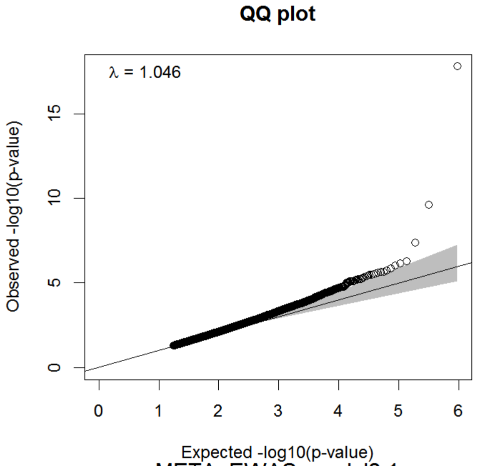

META_EWAS_model2.1

ESM Figure 2. Forest plots for 20 CpG sites showing considerable heterogeneity (I^2>60%; p<0.05) from results of meta-EWAS in incident type 2 diabetes.

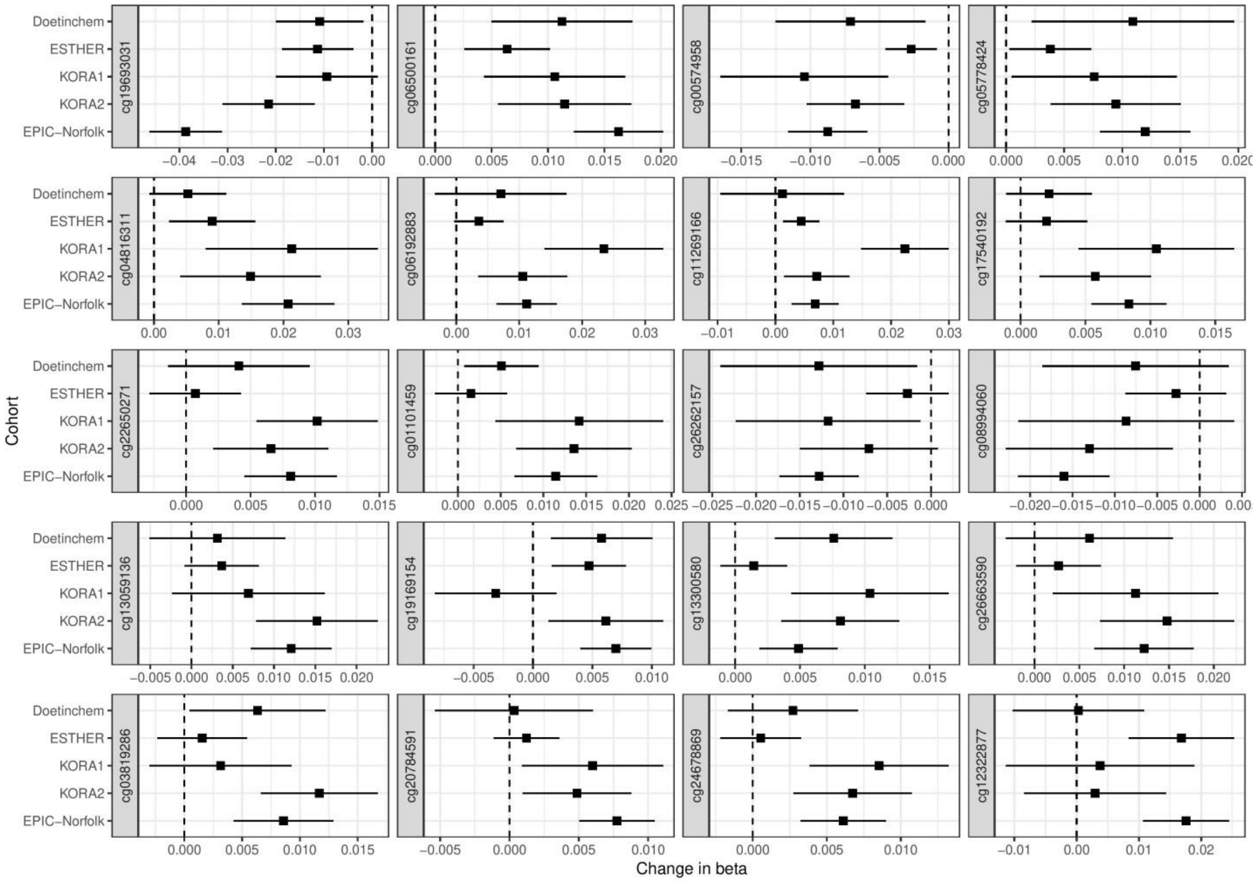

ESM Figure 3. Correlation plots of effect sizes across models in meta-EWAS in incident type 2 diabetes and across discovery and replication stage.

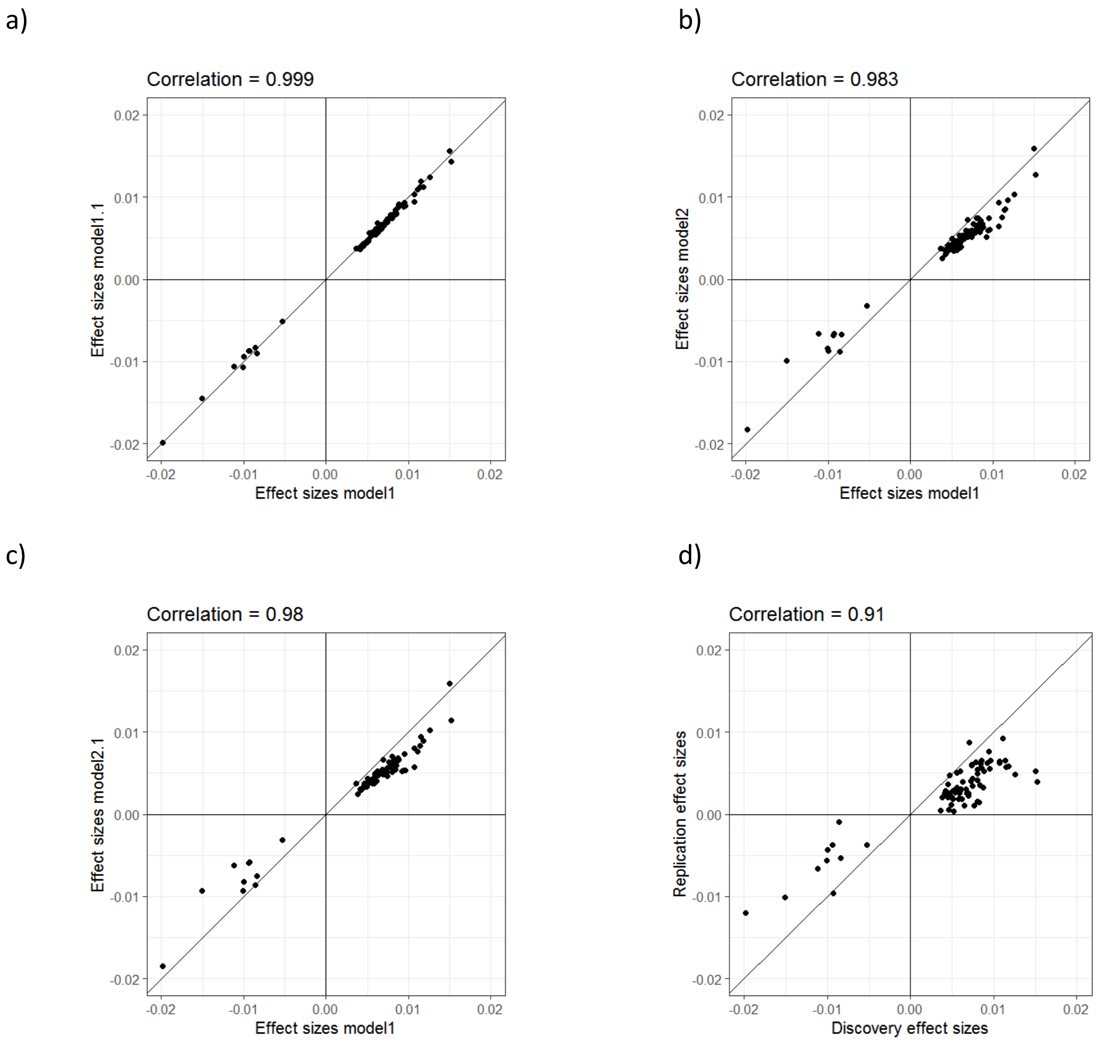

a, b, c: details of the models:
Model 1: Methylation values (normalized betas[0:1])~ Diabetes (0,1)+sex +age +Cell types+ Batches
Model 1.1: Model 1+ time + smoking
Model 2: Model 1 + BMI
Model 2.1: Model 1 + BMI + time + smoking
d: Effect sizes from model 1 from discovery and replication cohorts

ESM Figure 4. Predictive ability of methylation risk score based on 76 genome-wide significant CpG sites for incident T2D in LOLIPOP cohort.

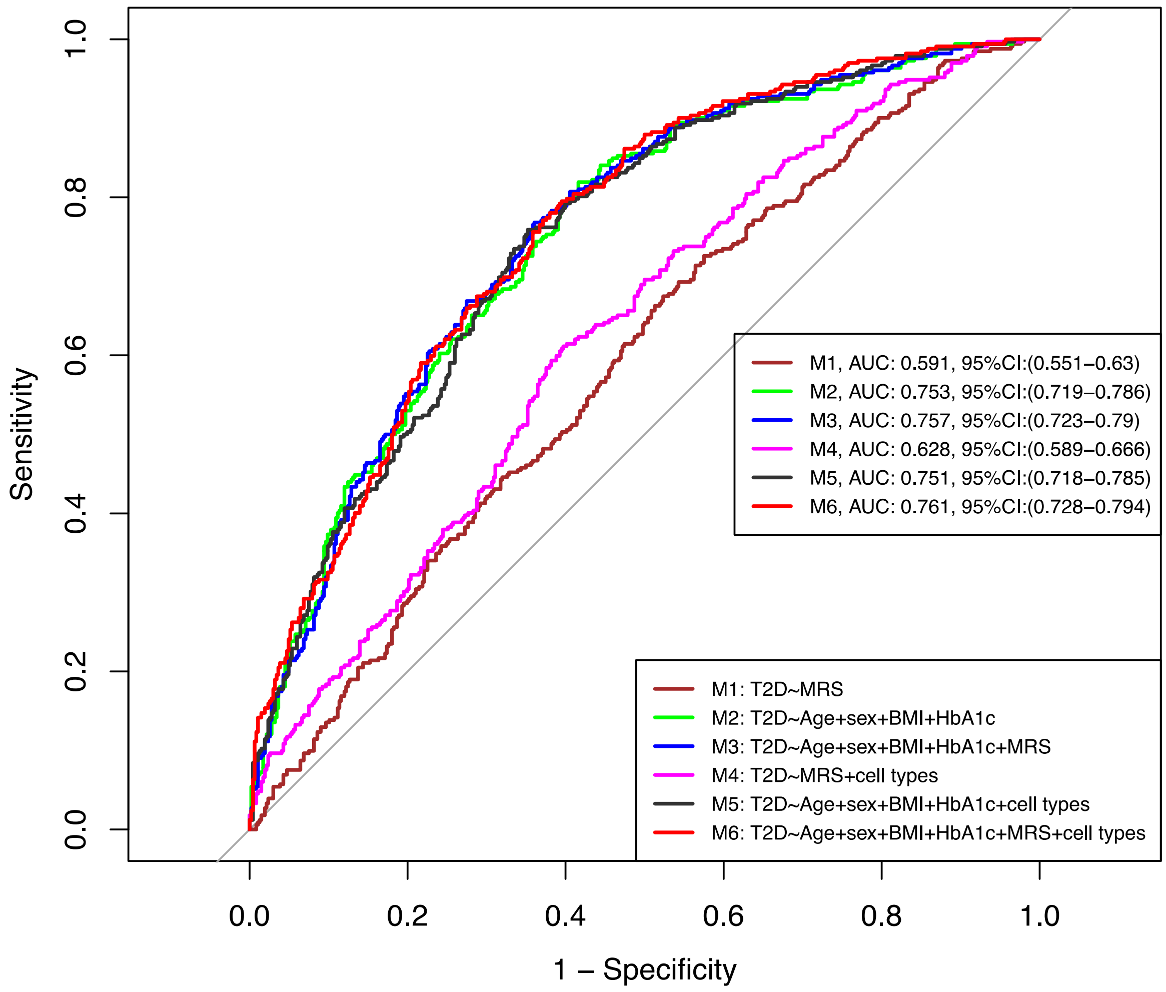

ESM Figure 5. Predictive ability of methylation risk score based on 69 genome-wide significant CpG sites for incident T2D in the Doetinchem cohort.

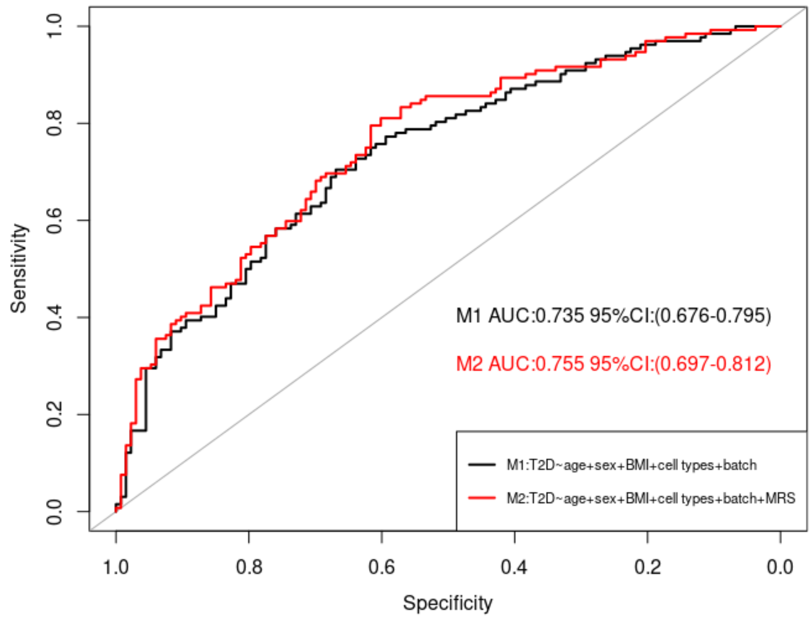

ESM Figure 6. Predictive ability of methylation risk score based on CpG sites at 4 increasingly lenient p-value thresholds for incident T2D in the Doetinchem cohort.

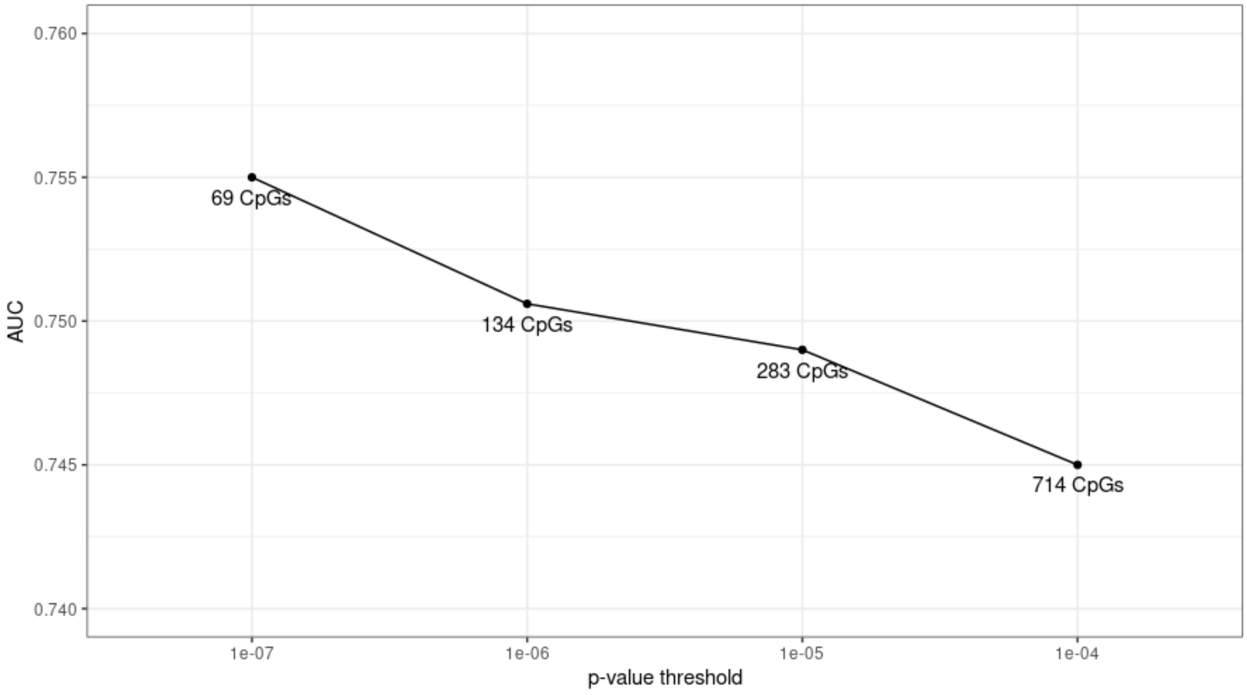

Supplement: Supplementary file 1 — (PDF 4171 kb) [file 125_2022_5652_MOESM1_ESM.pdf]
